# Identification of Indian Spiders through DNA barcoding: Cryptic species and species complex

**DOI:** 10.1038/s41598-019-50510-8

**Published:** 2019-10-01

**Authors:** Kaomud Tyagi, Vikas Kumar, Shantanu Kundu, Avas Pakrashi, Priya Prasad, John T. D. Caleb, Kailash Chandra

**Affiliations:** Centre for DNA Taxonomy, Molecular Systematics Division, Zoological Survey of India, M- Block, New Alipore, Kolkata, 700 053 West Bengal India

**Keywords:** Taxonomy, Entomology

## Abstract

Spiders are mega diverse arthropods and play an important role in the ecosystem. Identification of this group is challenging due to their cryptic behavior, sexual dimorphism, and unavailability of taxonomic keys for juveniles. To overcome these obstacles, DNA barcoding plays a pivotal role in spider identification throughout the globe. This study is the first large scale attempt on DNA barcoding of spiders from India with 101 morphospecies of 72 genera under 21 families, including five endemic species and holotypes of three species. A total of 489 barcodes was generated and analyzed, among them 85 novel barcodes of 22 morphospecies were contributed to the global database. The estimated delimitation threshold of the Indian spiders was 2.6% to 3.7% K2P corrected pairwise distance. The multiple species delimitation methods (BIN, ABGD, GMYC and PTP) revealed a total of 107 molecular operational taxonomic units (MOTUs) for 101 morphospecies. We detected more than one MOTU in 11 morphospecies with discrepancies in genetic distances and tree topologies. Cryptic diversity was detected in *Pardosa pusiola*, *Cyclosa spirifera*, and *Heteropoda venatoria*. The intraspecies distances which were as large as our proposed delimitation threshold were observed in *Pardosa sumatrana*, *Thiania bhamoensis*, and *Cheiracanthium triviale*. Further, shallow genetic distances were detected in *Cyrtophora cicatrosa*, *Hersilia savignyi*, *Argiope versicolor*, *Phintella vittata*, and *Oxyopes birmanicus*. Two morphologically distinguished species (*Plexippus paykulli* and *Plexippus petersi*) showed intra-individual variation within their DNA barcode data. Additionally, we reinstate the original combination for *Linyphia sikkimensis* based on both morphology and DNA barcoding. These data show that DNA barcoding is a valuable tool for specimen identification and species discovery of Indian spiders.

## Introduction

Spiders are an ancient group of arthropods with their fossil records dating back to the Devonian period (~400 million years ago)^1–3^. They are one of the most diverse groups with 48,248 extant species and can be found everywhere except Antarctica^4,5^. Due to their high abundance and rich diversity, spiders have been studied as model organisms for ecological^6^, developmental^7^, evolutionary^8,9^, and behavioral^10^ studies. They are natural predators and play an important role in Integrated Pest Management (IPM) in agriculture and horticulture ecosystems^11,12^. Spiders have been used as bio-control agents for various diseases causing mosquitoes^13^ and act as indicator species for environmental monitoring^14^. The silk produced by spiders has potential applications in biomedical, and defense avenues^15^. As compared with the global diversity, approximately 3.72% (1,799 species) are currently known from India^4^. Taxonomic research on spiders is rapidly progressing with many new species being described every year (on an average ~813 new species have been described each year from 2010 to 2018). However, a recent study predicted that the species richness of spider could be at least 120,000 worldwide^3^; hence, a large proportion of spider diversity remains yet to be discovered or described.

The morphological identification of this group is time consuming and challenging due to (i) sexual dimorphism (ii) polymorphism, and (iii) lack of identification keys for juveniles^16,17^. Considering these obstacles, it is important to use additional tools, which, may help in rapid species identification and resolving the taxonomic ambiguities. DNA barcoding has been widely used in the last decade in biodiversity research for accurate species identification^18^, resolving taxonomic questions^19^, detection of cryptic species and species complexes^20^, population estimation^21^, food adulteration^22^, wildlife forensics^23^, and invasive species detection^24^. The DNA barcode data can also be integrated in the phylogenetic studies^25^. The utility of DNA barcoding is also evidenced in spider identification from all life stages across the globe^26–33^. A recent DNA barcoding study of spiders from India included 17 morphologically identified species^34^. However, the present study is to date the largest barcoding attempt from India, including 101 morphologically identified species. We investigate the efficacy of DNA barcoding in species identification of specimens collected from eight different states of India. This work contributes 85 novel sequences of 22 morphologically identified species and establishes a comprehensive DNA barcode library of spiders from India to enrich the global database for future taxonomic research.

## Materials and Methods

### Sample collection and taxonomic identification

A total of 489 spider specimens were sampled from various locations across India, including eight states; Arunachal Pradesh, Assam, Chhattisgarh, Gujarat, Karnataka, Kerala, Rajasthan, and West Bengal (Fig. S1). Specimens were collected during 2015–2018 by various collection methods like hand picking, sweep netting, vegetation beating, and pitfall traps. The study does not involve any endangered or protected spider species. Thus, no prior permission was required for the collection. Specimens were preserved in molecular grade, 90% ethanol and stored in −20 °C. All the studied voucher specimens have been deposited in the National Zoological Collections (NZC), Zoological Survey of India (ZSI), Kolkata (Fig. S2, Table S1). The enlarged photographs of the genitalia of possible cryptic species and species complexes were also provided in Fig. S3. The morphological examination of all the specimens was done by using a Leica EZ4 HD stereomicroscope. All images were processed with the aid of the LAS core software (LAS EZ 3.0). The specimens were identified through available morphological keys and literatures (Table S2). Further, the genital characters (male pedipalps and the female epigyne) were dissected with the help of sterilized surgical scalpel blades and acquired the photographs by using Leica M205A for further confirmation. Photographs of the voucher specimens, male genitalia (dorsal and ventral view), and female genitalia (external and internal epigyne) used here were taken by the authors (JTDC, PP, AP). The collection locality map was prepared using Natural Earth public domain base maps (http://www.naturalearthdata.com/), with sampling points (yellow-red dots) added manually in Adobe Photoshop CS 8.0. Photographs of the studied specimens are linked with their pruned phylogenetic position by sharing coloured boxes.

### DNA extraction, PCR amplification and sequencing

For genomic DNA extraction, one leg of each specimen was removed and processed by using the QIAamp DNA Investigator Kit (Qiagen, Valencia, CA). The genomic DNA was quantified by using a Qubit fluorometer (Life Technologies, USA) and stored at −20 °C in Centre for DNA Taxonomy, ZSI, Kolkata. DNA barcode region of mitochondrial cytochrome oxidase subunit I (mtCOI) gene was amplified using the primer pairs: LCO1490 and Chelicerate Reverse 1; LCO1490 and Chelicerate Reverse 2^27,35,36^. Total 25 μl reaction volume containing 10 picomoles of each primer, 2.0 mM MgCl_2_, 0.25 mM of each dNTP, and 1U of Taq polymerase (Takara BIO Inc., Japan) with the following thermal profile: 5 min at 95 °C; followed by 5 cycles of 30 s at 95 °C, 40 s at 47 °C, 1 min at 72 °C and 30 cycles of 30 sec at 95 °C, 40 sec at 51 °C and 1 min at 72 °C and final extension for 7 min at 72 °C. The amplified PCR products were checked in 1% agarose gel and purified by using the QIAquick Gel Extraction Kit (Qiagen, Valencia, CA) following the manufacturer’s protocols. For bi-directional sequencing, the cycle sequencing was performed with BigDye®Terminator ver. 3.1 Cycle Sequencing Kit (Applied Biosystems, Inc.) Using 3.2 picomoles of both PCR primer pairs on the ABI Multiplex Thermal Cycler with following thermal profile: 96 °C for 1 min, then followed by 25 cycles of 96 °C for 10 s, 50 °C for 5 s and a final extension at 60 °C for 1 min 15 s. The cycle sequencing products were cleaned by using BigDye X-terminator kit (Applied Biosystems Inc.) and used in-house facilities (48 capillary ABI 3730 Genetic analyzer) for sequencing.

To obtain the consensus sequences, both forward and reverse chromatograms were checked by the SeqScape software version 2.7 (Applied Biosystems Inc.). Further, the sequences were screened through nucleotide BLAST program (https://blast.ncbi.nlm.nih.gov) and ORF finder (https://www.ncbi.nlm.nih.gov/orffinder/) to assure the absence of gaps, indel (insertion/deletions) and stop codons. The generated sequences were submitted to GenBank through Bankit submission tool (https://www.ncbi.nlm.nih.gov/WebSub/?tool=genbank) and Barcode of Life Data Systems (BOLD) under the project ‘DNA barcoding of Spiders of India’ for acquiring the accession numbers and BOLD-IDs. The mite (Arachnida: Acari) sequence (BOLDMSACA57112_OG_Acari) was used as an out-group in the present dataset^31^.

### Genetic distance, haplotyping, and tree analysis

The dataset was aligned using MAFFT algorithm in the CIPRES web portal (http://www.phylo.org/)^37^ and the noisy parts were trimmed from both the ends to make a uniform sequence length. MEGAX^38^ with Kimura-2 parameter (K2P) was used to calculate the pairwise genetic distances within families, genera, and species. The representation of genetic distances of within and between the species was plotted by BoxPlotR (http://shiny.chemgrid.org/boxplotr/). The haplotype data were generated using DnaSP5.10^39^. Two tree building methods, Neighbor-Joining (NJ), and Bayesian analysis (BA) were applied to examine the tree based species identification with reciprocal monophyly criteria. The NJ tree was generated in MEGAX with Kimura-2 parameter (K2P) model and 1000 bootstrap supports. Partition Finder version 1.1.144^40^ and jModel test^41^ were used to choose the best fit model for the dataset. Both computational methods suggested ‘GTR + I + G’ for all three codon positions with the lowest BIC value. The BA tree was constructed in Mr. Bayes 3.1.2^42^ by selecting nst = 6 and four (one cold and three hot) metropolis-coupled Markov Chain Monte Carlo (MCMC), was run for 50,000,000 generations with 25% burn in and trees saving at every 100 generations. The MCMC analysis was used to generate the convergence metrics, till the standard deviation (SD) of split frequencies reached under 0.01 and the potential scale reduction factor (PSRF) for all parameters approached 1.0. To represent the generated tree topologies, the web based iTOL tool (https://itol.embl.de/)^43^ was used.

### Species delimitation and MOTUs estimation

To estimate the Molecular Operational Taxonomic Units (MOTUs), four species delimitation methods: Automatic Barcode Gap Discovery (ABGD)^44^, the General Mixed Yule-coalescent (GMYC)^45^, Poisson-Tree-Processes (PTP)^46^, and Barcode Index Number (BIN)^47^ (http://www.boldsystems.org) were applied. The ABGD analysis was performed on the web server (www.abi.snv.jussieu.fr/public/abgd/) with the Jukes-Cantor (JC69) and p-distance with relative gap width (X = 1.5). For GMYC analysis, the ultrametric tree was generated in BEAST^48^ by using the Yule model, relaxed lognormal clock, GTR + I + G model and run for 50 million generations, with a sampling frequency of every 100 generations. Further, the Tree Annotator^48^ was used for analyzing the output tree with following settings; 10% burn-in, 0.5 posterior probability limits, and the node heights of the target tree. Both single and multiple thresholds of GMYC analysis were carried out in RStudio (https://www.r-project.org/) using packages like ‘ape’^49^ and ‘splits’^50^. For bPTP analysis (http://species.h-its.org/ptp/), ML tree was constructed in RAxML^51^ based on the haplotype data with GTR + I + G. The BINs were estimated on BOLD workbenchv3.6 (http://www.boldsystems.org).

## Results and Discussion

### Morphospecies identification

A total of 489 specimens of 72 genera under 21 families in two suborders (Araneomorphae and Mygalomorphae) was examined in this study. Among them, 468 specimens were represented by 85 morphospecies in 58 genera under 18 families. The remaining 21 specimens were identified only up to genus level representing 14 genera due to the unavailability of morphological keys for juveniles and sub-adults (Fig. S2). Therefore, the present study generated the DNA barcode data of 101 morphospecies for further analysis. This data also include three recently described species (*Epocilla sirohi* Caleb, Chatterjee, Tyagi, *et al*.; *Mogrus rajasthanensis* Caleb, Chatterjee, Tyagi, *et al*.; *Pseudopoda cheppe* Caleb)^52,53^ and two published new records (*Psechrus inflatus* Bayer and *Menemerus nigli* Wesołowska & Freudenschuss)^54,55^ from India.

### Delimitation threshold analysis and haplotyping

**Figure 1:**
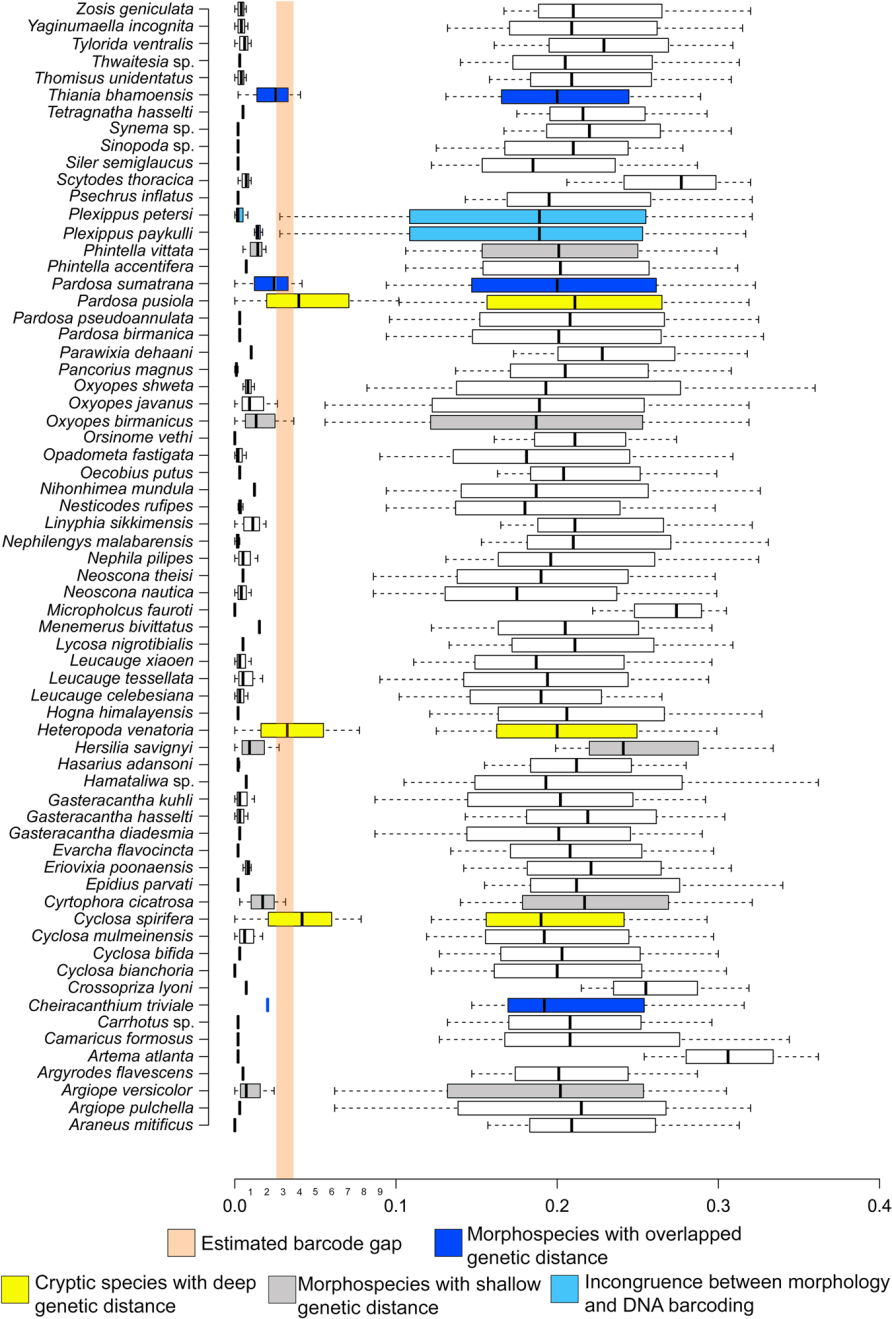
DNA barcoding gaps (marked by peach color) for Spider, based on the intra- and interspecies K2P genetic distances. Center lines show the medians; whiskers extend to the minimum and maximum values. Cryptic species and morphospecies with incongruent genetic distances showed in different colors. Singleton species are excluded from the data analysis. The representation of genetic distances of within and between the species was plotted by BoxPlotR (http://shiny.chemgrid.org/boxplotr/) and edited manually in Adobe Photoshop CS 8.0.

We generated and analyzed 489 barcode sequences in the current study. Among them, 85 sequences of 22 morphospecies were a new contribution to the global databases (GenBank and BOLD). The genetic distance between the two suborder Araneomorphae and Mygalomorphae was 28.4%. The intergeneric genetic distances were ranged from 8.69% to 28.59%. The interspecific genetic distances were ranged from 2.11% (*Plexippus*) to 22.30% (*Scytodes*) with a mean of 9.32%. The intraspecific genetic distances were ranged from 0% to 10.09% with mean 1.04% (Table 1). The intraspecies genetic distance was high due to the possible cryptic diversity in *Pardosa pusiola* (10.09%), *Cyclosa spirifera* (7.7%), and *Heteropoda venatoria* (7.6%). Excluding these species, the maximum intraspecies genetic distance of the dataset drops down to 2.6%. Previously, the DNA delimitation threshold of arachnids was estimated from 2% to 3.6%^26,27,31,33,34^, however, in the current study, we propose a delimitation threshold between ‘2.6% to 3.7%’ (lowest interspecies > highest intraspecies genetic distance) (Fig. 1). Although several studies discussed the delimitation threshold of the spider fauna across the globe, the recent estimation would be helpful to detect distinct spider species from India. The haplotype analysis revealed a total of 247 unique haplotypes for the dataset (Table S1).

**Table 1:** Genetic distance (K2P) of the 101 studied spider species in the present study. The genetic distance marked with star showed incongruence.

	Comparisons	Min Distance (%)	Mean Distance (%)	Max Distance (%)
Within Species	3438	0.00	1.04	10.09*
Within Genus	2906	2.11*	9.32	22.30
Within Family	13892	8.69	16.26	28.59

### Tree analysis and MOTU estimation

**Figure 2:**
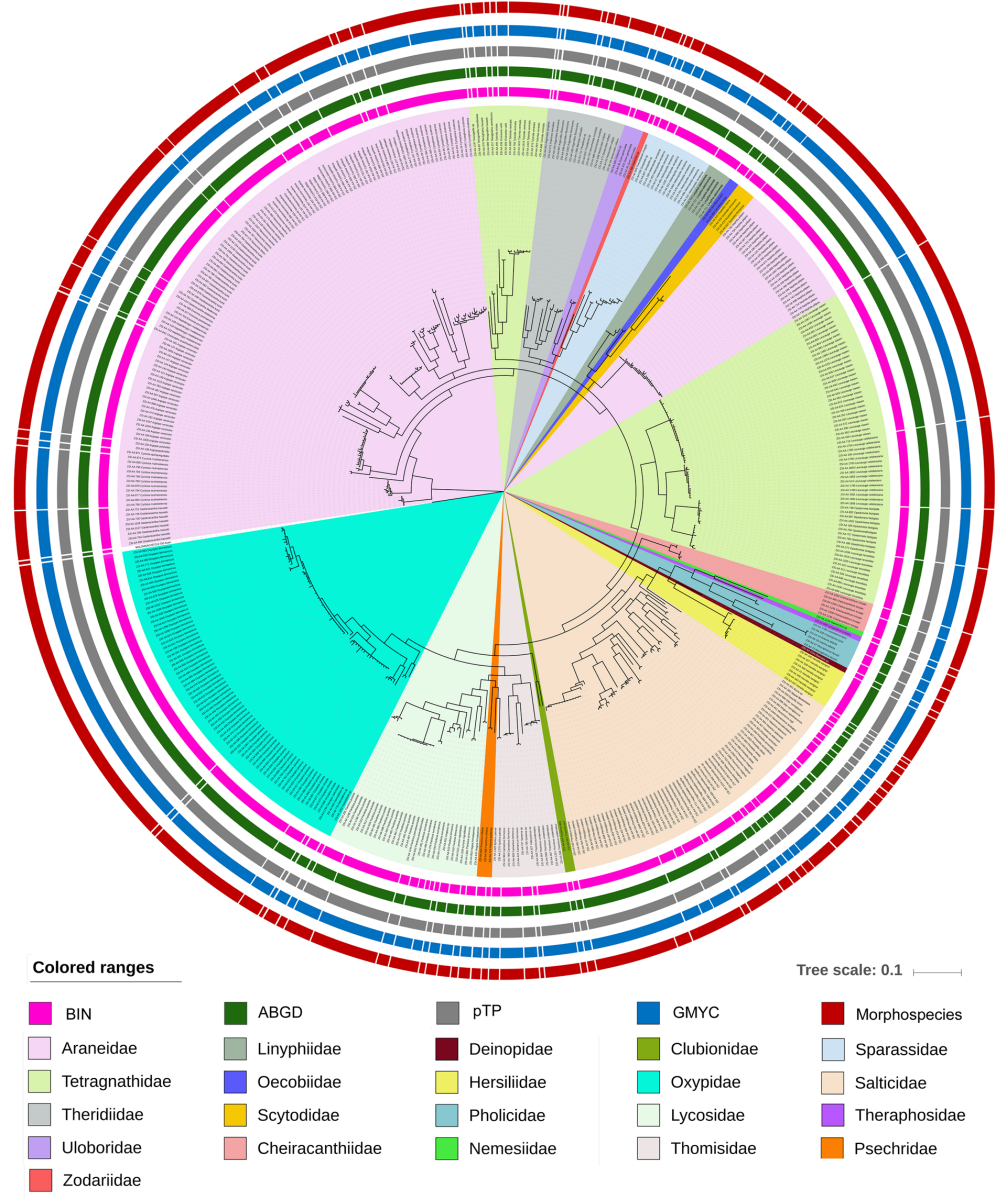
Bayesian inference tree with delineated MOTUs of the studied 101 spider species. The 21 families were highlighted by color corresponding to the clade. Color bars indicate delineated MOTUs by different methods (Morphospecies, ABGD, GMYC, BIN, and PTP). The tree topologies were plotted by web based iTOL tool (https://itol.embl.de/itol.cgi) and edited manually in Adobe Photoshop CS 8.0. The iTOL web server was used with proper guidelines of the European Molecular Biology Laboratory (EMBL) under the following attribution: https://www.embl.de/aboutus/privacy_policy/index.html.

Both BA and NJ tree building methods yielded similar topologies. The BA and NJ trees showed cohesive clustering for 88 morphospecies with high posterior probabilities and bootstrap supports (Figs 2 and S4). The remaining 13 morphospecies showed topological discrepancies in both NJ and BA. To estimate the genetic diversity by using single mitochondrial gene sequences, we used multiple species delimitation methods. The taxon below the species level is indicated by the Molecular Operational Taxonomic Units (MOTUs)^20^. Four species delimitation methods: BIN, ABGD, GMYC, and PTP yielded almost similar results and identified 111, 103, 120, and 106 MOTUs respectively (Fig. S5, Tables S1, S3 and S4). Therefore, by superimposing the multiple delimitation methods, and reciprocal monophyly criteria, a total 107 MOTUs were detected in the studied dataset.

### Cryptic species with deep genetic distance

**Figure 3:**
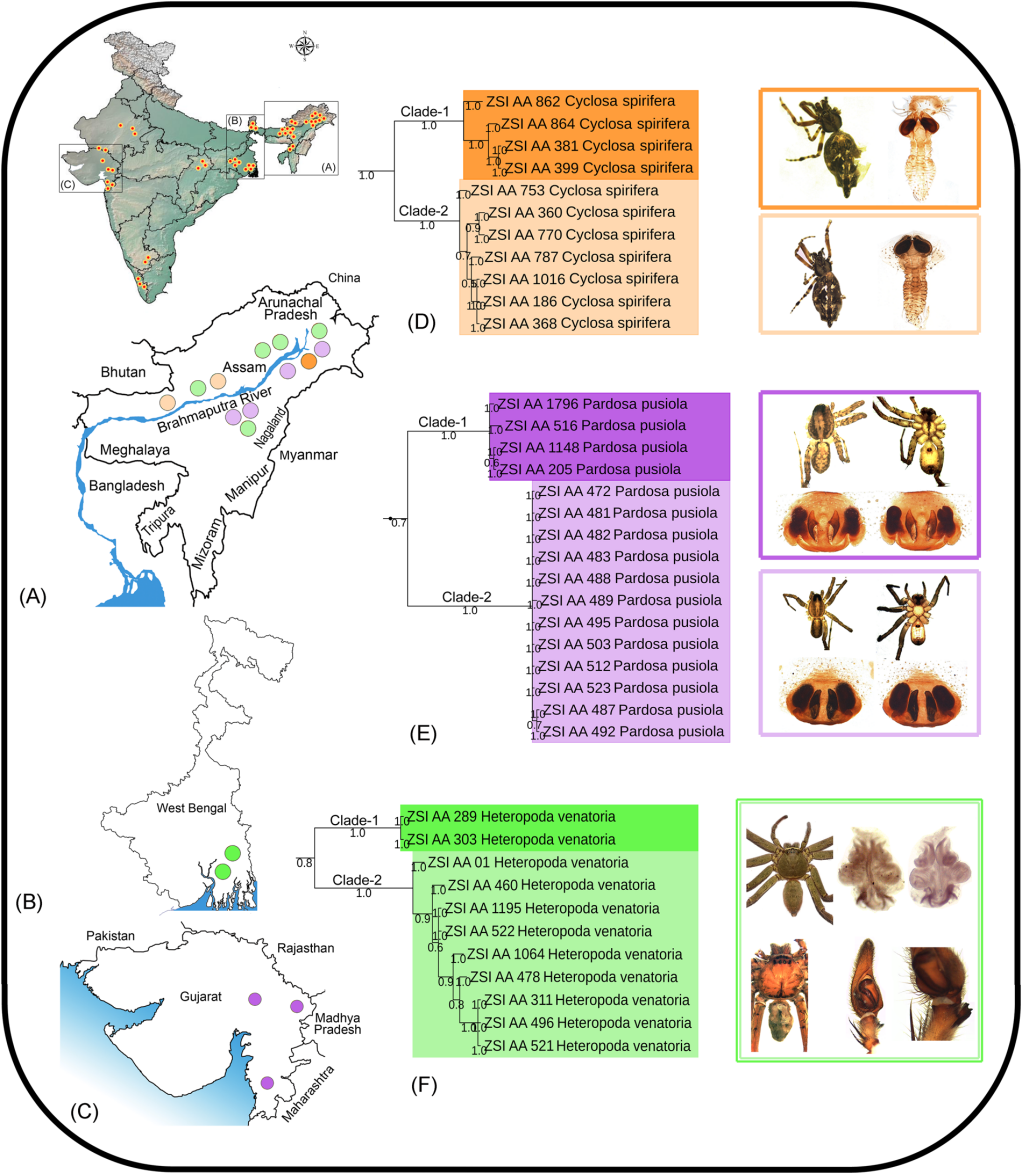
**(A)** The collection localities of the studied spiders in northeastern region, (**B**) West Bengal, (**C**) Gujarat state in India. (**D**) Pruned BA tree showing cryptic diversity of *Cyclosa spirifera*, (**E**) *Pardosa pusiola*, and (**F**) *Heteropoda venatoria*.

Recent molecular studies on spiders have revealed cryptic speciation from different geographical regions^26,31,34^. In the present study, we detected three species with high genetic distances and more than one clade in both NJ and BA trees, which speculates a possibility for cryptic diversity (Table 2). *Cyclosa spirifera* is endemic to India, which was originally described by Simon^56^ from the Uttarakhand state of the western Himalayas. After a long gap of 118 years, the species was reported from the other parts of central (Madhya Pradesh, Maharashtra) and eastern (West Bengal) India^4^. We examined 11 specimens from Assam state of northeast India showed two distinct clades (Clade-1 and Clade-2) corresponding to two separate MOTUs with 7.4% genetic distance. All the species delimitation methods showed concordant results with the tree based analysis and correspond with their collection localities in opposite banks of the Brahmaputra River. Morphologically, *C*. *spirifera* shows variation in the length of the caudal protrusion (longer in Clade-1 and shorter in Clade-2), but we could not find any other morphological difference between the specimens of these two clades. (Fig. 3A,D).

**Table 2:** Number of Molecular Operational Taxonomic Units (MOTUs) in 11 morphospecies detected by multiple species delimitation methods.

Sl No.	Species	BIN	ABGD	GMYC	PTP
1	*Cyclosa spirifera*	2	2	2	2
2	*Pardosa pusiola*	2	2	2	2
3	*Heteropoda venatoria*	2	2	3	2
4	*Pardosa sumatrana*	4	1	2	1
5	*Cheiracanthium triviale*	2	2	2	2
6	*Thiania bhamoensis*	3	1	3	2
7	*Oxyopes birmanicus*	4	1	3	1
8	*Hersilia savigyni*	1	1	2	2
9	*Phintella vittata*	1	1	1	2
10	*Argiope versicolor*	1	1	2	1
11	*Cyrtophora cicatrosa*	1	1	2	1

The lycosid species, *Pardosa pusiola* was described by Thorell^57^ from Sumatra in Southeast Asia. The species is widely distributed in the Oriental region from India to China, Java, and Sumatra^4^. A total of 16 specimens of *P*. *pusiola* were collected from two distant geographical regions in India, among them four from Gujarat state (western region) and 12 from Assam state (northeastern region). The studied specimens of *P*. *pusiola* showed two distinct clades (Clade-1 and Clade-2) in both NJ and BA trees with 9.8% genetic distance. The four species delimitation methods revealed two MOTUs in concordance with the tree based analysis. Previous morphological studies observed few minor variations in the internal structure of female genitalia in *P*. *pusiola*^58^. We also studied the similar morphological variations in the Indian specimens. The specimens of Clade-1 (Gujarat) and Clade-2 (Assam) can be distinguished by the following morphological characters: body is highly pigmented in Clade-1 (brown in Clade-2); sternum with the median longitudinal band in Clade-1 (absent in Clade-2); epigynal pockets are diverging anteriorly in Clade-1 (parallel in Clade-2) (Fig. 3A,C,E).

The cosmopolitan species, *Heteropoda venatoria* (Sparassidae) was originally described as *Aranea venatoria* from Asia by Linneaus^59^. Later on, this species was described under various names by several authors^4^. We examined nine specimens from Assam and two from West Bengal. The NJ and BA tree showed two clades (Clade-1 and Clade-2) with 7.4% genetic distance. The ABGD, BIN and PTP analysis indicate two MOTUs which are concordant to the tree topologies, while GMYC showed three MOTUs. The external and internal morphological examination of genitalia (illustrations provided by Jäger^60^) was found to be similar for all specimens under two clades (Fig. 3A,B,F). The similar morphology of the studied specimens with high genetic distance revealed two distinct populations of *H*. *venatoria* from two distant localities of Assam and West Bengal state.

The observed genetic diversity in *C*. *spirifera*, *P*. *pusiola*, and *H*. *venatoria* is worthy of future investigation to evaluate the presence of cryptic species within these taxa. This research will require the comparison of specimens of the distinct clades with the type specimens for the species and synonyms and molecular data from specimens collected from type localities, in order to reveal which clades represent the nominal taxa.

### Morphospecies with higher intraspecies genetic distance

A previously defined genetic distance can be used as a delimitation threshold for species discrimination^27,61^. This study detected three morphospecies, *Thiania bhamoensis*, *Pardosa sumatrana*, and *Cheiracanthium triviale*, which had intraspecific genetic distances which were around our proposed delimitation threshold (2.6% to 3.7%).

**Figure 4:**
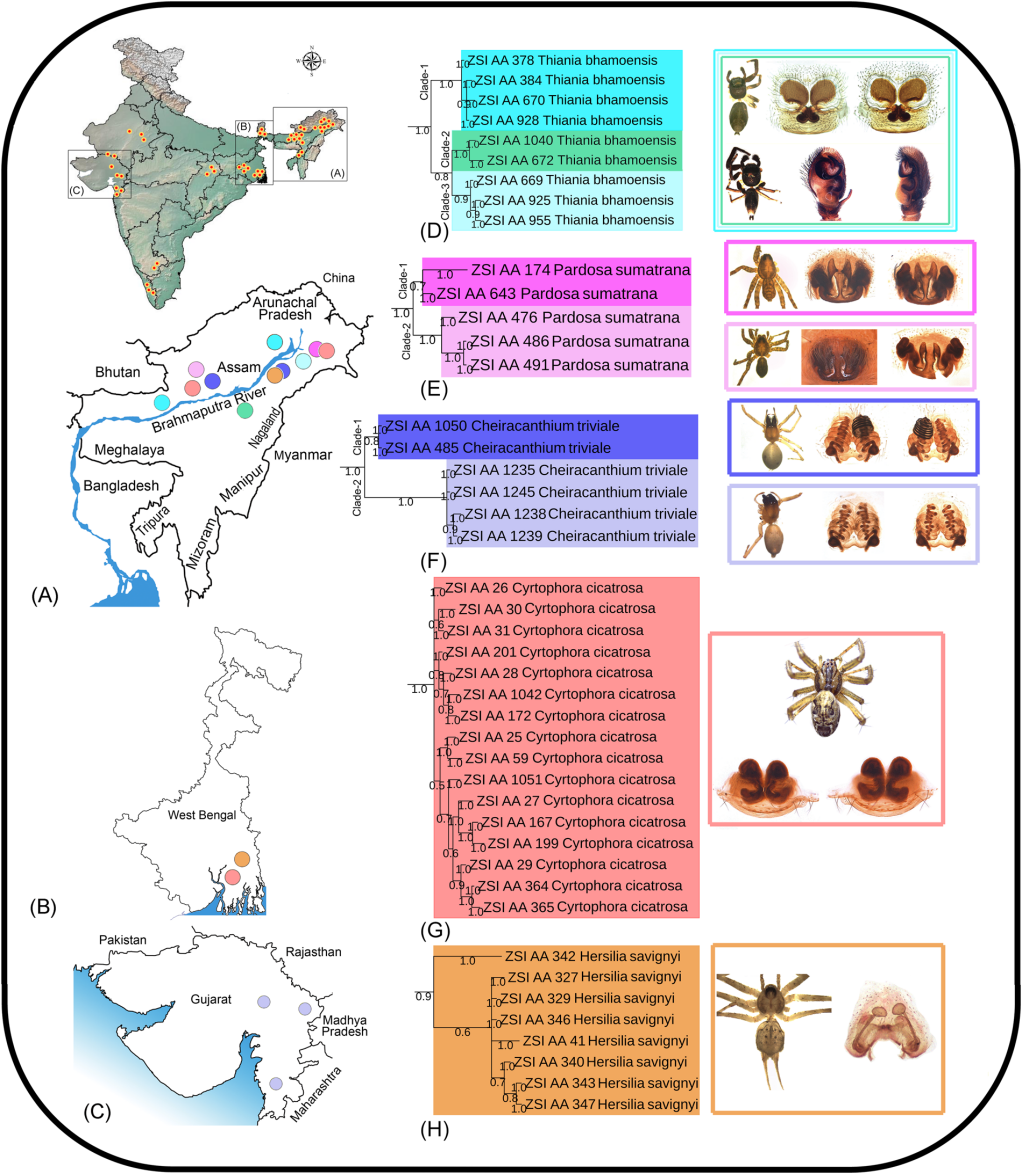
**(A)** The collection localities of the studied spiders in northeastern region, (**B**) West Bengal, (**C**) Gujarat state in India. (**D**) Pruned BA topologies of *Thiania bhamoensis*, (**E**) *Pardosa sumatrana*, and (**F**) *Cheiracanthium triviale* with higher intraspecies genetic distance. (**G**) Pruned BA topologies of *Cyrtophora cicatrosa*, and (**H**) *Hersilia savignyi* with shallow genetic distance.

The salticid species, *T*. *bhamoensis* was first described by Thorell^62^ from Bhamo, Myanmar. This species is widely distributed from India to Myanmar, China, Thailand, Laos and up to Indonesia^4^. A total of nine specimens were collected from the opposite banks of the Brahmaputra River in Assam. Both NJ and BA trees infer well-defined clades (Clade-1, Clade-2, and Clade-3). Clade-1 showed 2.2% and 3.6% genetic distance between Clade-2 and Clade-3 respectively, while 3.5% genetic distance was detected between Clade- 2 and Clade-3 (Fig. 4A,D). Two species delimitation methods (BIN and GMYC) showed three MOTUs, which was concordant with the tree topologies, however ABGD and PTP showed one and two MOTUs respectively.

The lycosid species, *P*. *sumatrana* was described by Thorell^63^ from Sumatra in Southeast Asia. The species is widely distributed in the Oriental region from India to China, Java, and Sumatra. Five specimens of *P*. *sumatrana* were collected from the narrow geographical locations of Assam. All the studied specimens showed two clades (Clade-1 and Clade-2) in both NJ and BA trees with 3.2% genetic distance. Two species delimitation methods (ABGD and PTP) revealed one MOTUs which is concordant, while BIN and GMYC showed four and two MOTUs respectively, which is discordant to the tree analysis results and morphology (Fig. 4A,E). Further, the morphological characters of all the specimens were similar and resemble with ‘Type IV’ genital morph as suggested earlier^64^.

The cheiracanthiid species, *C*. *triviale* was originally described by Thorell^65^ as *Eutittha trivialis* from Burma based on the female. Later on, several authors studied the types as well as Indian collections and confirmed the distribution of *C*. *triviale* across India (Madhya Pradesh, Maharashtra, Manipur, Tamil Nadu, West Bengal)^66–68^. In the present study, out of six *C*. *triviale* specimens, four were collected from Gujarat and two from Assam. Both NJ and BA trees showed two distinct clades (Clade-1 and Clade-2) with 3.6% genetic distance. All species delimitation methods showed two MOTUs which are concordant to tree based analysis. We observed variation in the posterior margin of epigyne, which has a deep cleft in Clade-1 (Gujarat specimens) and shallow in Clade-2 (Assam specimens) (Fig. 4A,C,F).

Considering the observed higher intraspecies genetic distance within these three morphospecies, we assumed that the populations might incline to acquire genetic modifications rather than morphological variation. These genetic alterations may trigger to the morphology and evolution of possible new or cryptic species in near future.

### Morphospecies including possibly cryptic species

**Figure 5:**
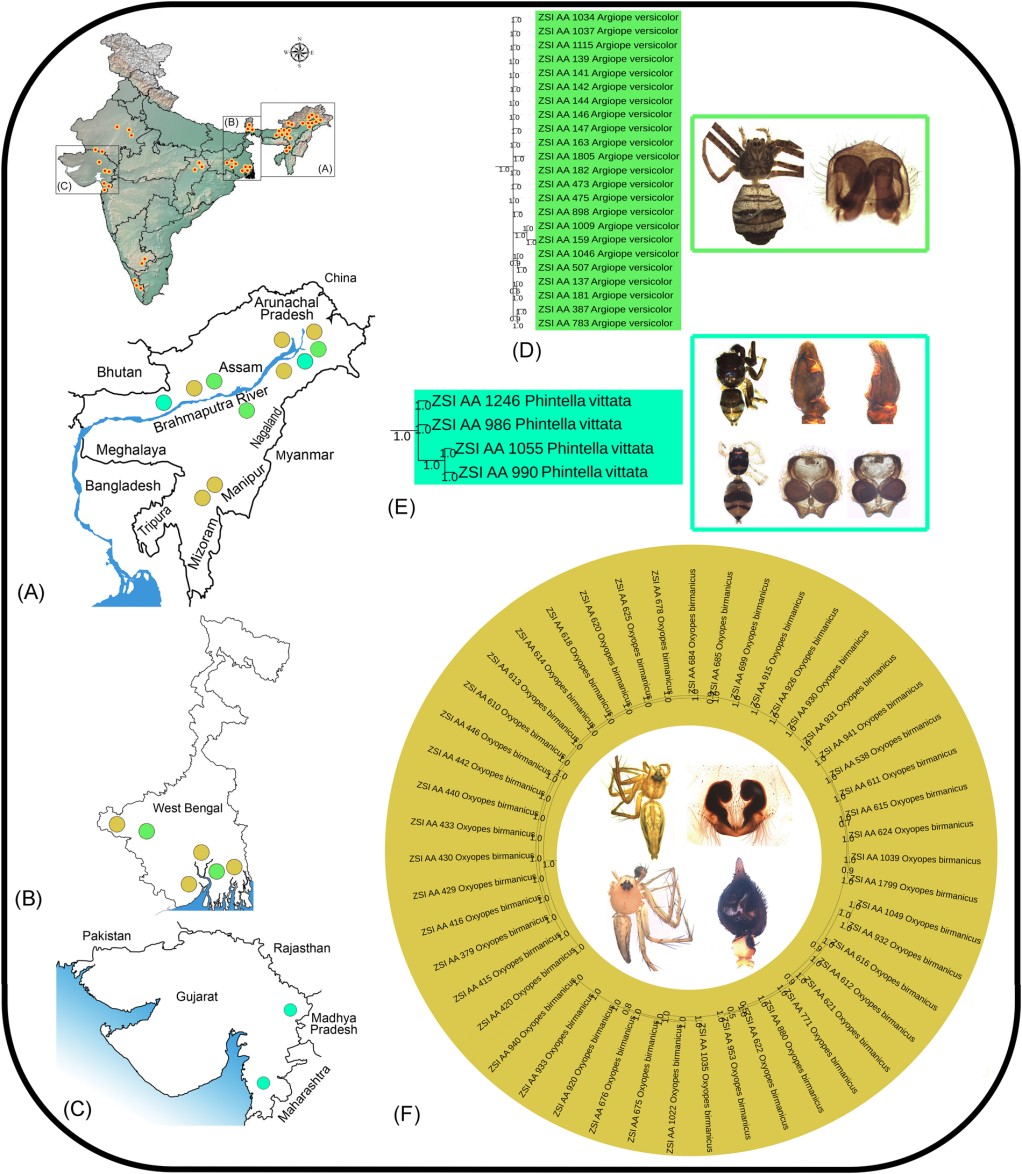
(**A**) The collection localities of the studied spiders in northeastern region, (**B**) West Bengal, (**C**) Gujarat state in India. (**D**) Pruned BA topologies of *Argiope versicolor*, (**E**) *Phintella vittata*, and (**F**) *Oxyopes birmanicus* with shallow genetic distance.

In the present study, five morphospecies (*Cyrtophora cicatrosa*, *Hersilia savignyi*, *Argiope versicolor*, *Phintella vittata* and *Oxyopes birmanicus*) showed shallow genetic distance with more than one MOTU by at least one species delimitation method. Both NJ and BA topologies were consistent to morphology based identification. Further, no significant morphological variations were observed in these species. A total of 16 specimens of *C*. *cicatrosa* were collected from Assam and West Bengal showed two MOTUs by GMYC method with 2% genetic distance (Fig. 4A,B,G). Eight specimens of *H*. *savignyi* were collected from Assam and West Bengal showed two MOTUs (GMYC and PTP) with 2.3% genetic distance between them (Fig. 4A,B,H). A total of 23 specimens of *A*. *versicolor* were collected from Assam and West Bengal showed two MOTUs (GMYC) with 1.6% genetic distance (Fig. 5A,B,D). Two specimens of *P*. *vittata* each from Assam and Gujarat revealed two MOTUs (PTP) with 1.8% genetic distance (Fig. 5A,C,E). A total of 47 specimens of *O*. *birmanicus* were collected from Assam and West Bengal showed three and four MOTUs with 1.8% to 3.4% and 2% to 2.9% genetic distance in BIN and GMYC methods respectively (Fig. 5A,B,F). The presence of multiple MOTUs with shallow genetic distance in these morphospecies demand more samples from wide geographical regions to conclude the possibility of cryptic species.

### Incongruence between morphology and DNA barcoding

**Figure 6:**
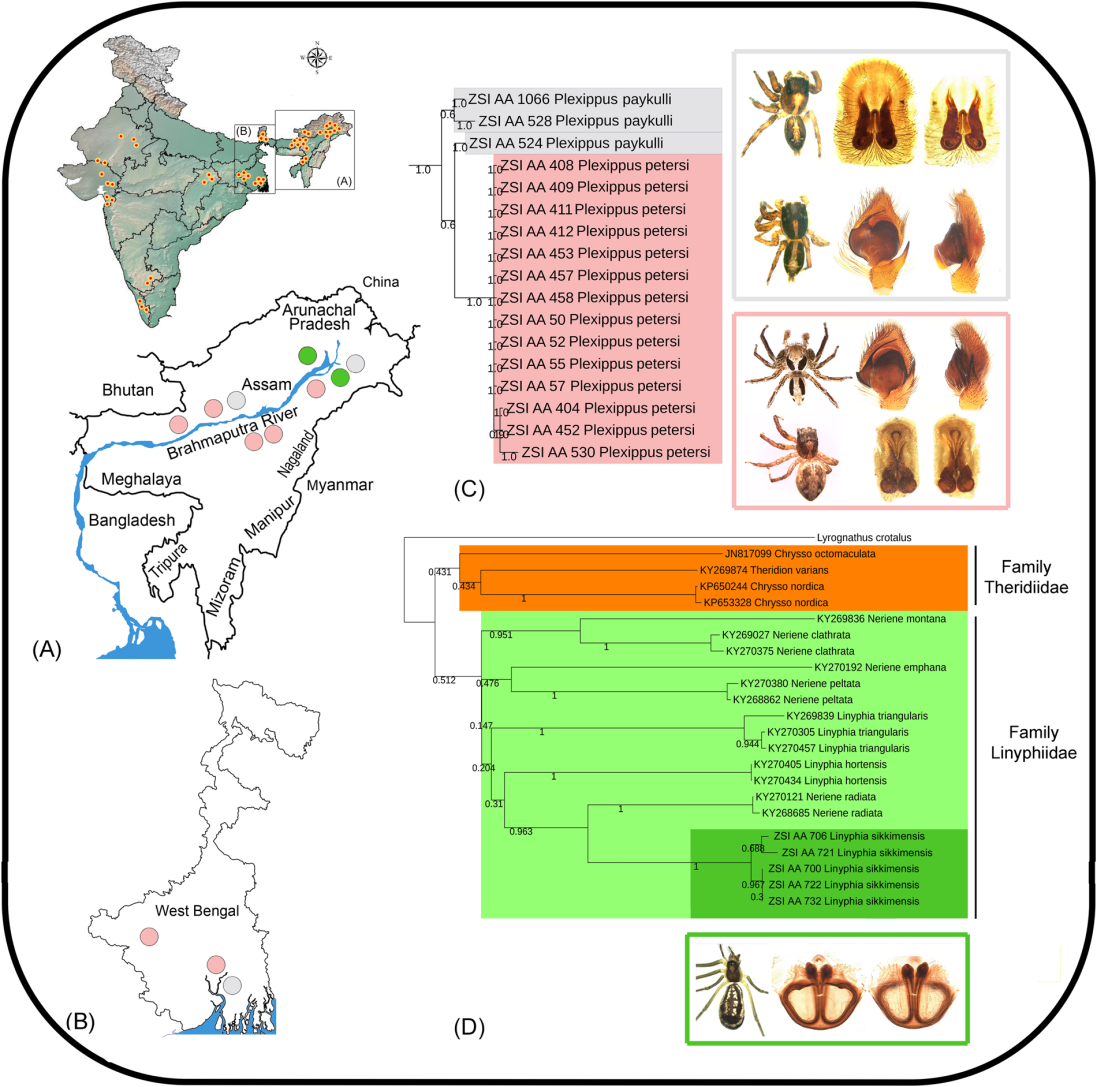
(**A**) The collection localities of the studied spiders in northeastern region, and (**B**) West Bengal in India. (**C**) Pruned BA topologies of *Plexippus paykulli*, and *Plexippus petersi* showed incongruence between morphology and DNA data (**D**) NJ tree showing the relationship of *Linyphia sikkimensis* with other Theridiids and Linyphiids species.

Two salticid species, *Plexippus paykulli* (Audouin)^69^ and *Plexippus petersi* (Karsch)^70^ were originally described from Africa and have wide distribution in the tropics. A total of three specimens of *P*. *paykulli* and 14 specimens of *P*. *petersi* were collected from West Bengal and Assam. The intraspecies genetic distances of morphologically identified *P*. *paykulli* and *P*. *petersi* were 0% to 1.2% and 0% to 0.8% respectively. The species were separated by a mean genetic distance of 2.6% (range = 2.1% to 3.6%), which is below the estimated delimitation threshold. Both NJ and BA tree had low bootstrap support for these clades (Fig. 6A–C). All four species delimitation methods showed these two morphospecies into a single MOTU. Thus, the DNA barcode data cannot clearly distinguish these two distinct morphospecies. However, these two morphospecies can be distinguished by their color pattern, and genital structures. The following distinguishable characters of both sexes are as follows, Male: Cephalothorax with median longitudinal white stripe in *paykulli* (absent in *petersi*), broad white band present in lateral side of clypeus or in front of anterior lateral eyes in *paykulli* (narrow in *petersi*), tibial apophysis shorter and reaching up to the half of the tegulum height in *paykulli* (longer and reaching up to the ¾ of the tegulum height in *petersi*), and embolus shorter in *paykulli* (longer in *petersi*). Female: epigyne wider in *paykulli* (narrower in *petersi*), copulatory opening crevices U-shaped in *paykulli* (V-shaped in *petersi*), insemination ducts long in *paykulli* (short in *petersi*), and central pocket small and located posteriorly to the copulatory openings in *paykulli* (large and located anteriorly in *petersi*)^71^. This kind of ambiguities between DNA barcoding and morphology has also been observed in other insect groups^20,72^. The possible reasons for this incongruency may be due to the introgression of mitochondrial DNA through interspecific hybridization^73^ and incomplete lineage sorting of ancestral mitochondrial DNA polymorphisms^74^.

### *Linyphia sikkimensis* Tikader, 1970 comb. rev

*Linyphia sikkimensis* was originally described in the Linyphiidae by Tikader^75^ based on 13 females and three males collected from the Sikkim state of India. Later on, Breitling^76^ transferred *L*. *sikkimensis* under the genus *Chrysso* based on distribution patterns and behaviour without any morphological evidences. Simultaneously, he also clearly stated that the generic as well as family level replacements of ‘*sikkimensis*’ from *Linyphia* (Linyphiidae) to *Chrysso* (Theridiidae) are tentative and doubtful. The morphological examination of five female specimens showed the resemblance towards the family Linyphiidae, which can be distinguished from Theridiidae by the following characters: tarsi IV without a comb, rebordered labium, and chelicerae usually with stridulating file. Further, the specimens were examined by the available generic keys provided by Helsdingen^77^ and fit to the couplet 1 (*Neriene*) with the distinguishing characters; large and conspicuous opening of epigyne, spirally coiled groove in the atria, absence of spiral tubes, and simple scape. However, the genus *Neriene* was earlier considered to be a synonym of *Linyphia* until it was reassessed thoroughly (van Helsdingen, 1969). Later on, some recent authors placed the genus *Neriene* as a subgenus of *Linyphia*^4^. A total of five specimens of *Linyphia sikkimensis* were collected from Assam state (four from Dehing-Patkai Wildlife Sanctuary and one from Dibru-Saikhowa National Park) with 0% to 1.9% intraspecies genetic distance. The haplotype analysis also revealed four distinct haplotypes within these two clades. All species delimitation methods showed two MOTUs, concordant to tree topologies. To test the generic placement of *L*. *sikkimensis*, 24 DNA barcode sequences (five generated sequences of *L*. *sikkimensis*, 10 GenBank sequences of five *Neriene* species, six GenBank sequence of two *Linyphia* species, three GenBank sequences of two *Chrysso* species) were further analysed (Table S5). The genetic distance of *Neriene* was 15.4% and 16.5% as compared with *Linyphia* and *Chrysso* respectively. The genera *Linyphia* and *Chrysso* showed 18.1% genetic distance from each other. Further, the generated sequences of *L*. *sikkimensis* showed 15.6% and 15.7% genetic distances with the congeners of *Neriene* and *Linyphia* respectively. The congeners of *Neriene* and *Linyphia* also maintained 15.8% genetic distance with each other. The BA tree showed distinct clade of *Chrysso* as compared with both sister genera *Linyphia* and *Neriene*. However, the studied species *L*. *sikkimensis* closely clustered with *Linyphia* + *Neriene* clade (Fig. 6A,D). On the basis of above mentioned morphological conflicting facts, high genetic dissimilarities, and low posterior probability support in BA tree, we proposed the generic replacement of ‘*sikkimensis*’ from *Chrysso* to the original genus in which it was described. We recommend to generate the sequences of more mitochondrial and nuclear markers with large scale sampling effort to determine the actual relationship and systematic position of *L*. *sikkimensis*.

In the recent past, DNA barcoding evidenced as an effective supplementary tool for accurate species identification and biodiversity research. However, this emerging technique often shows defects to resolve several biological questions due to the lack of appropriate experimental design^78^. To overcome the methodological deficiencies, we sampled the broader geographical regions in India, and accurately identified the specimens through taxonomic characters. The generated DNA barcode data were further analysed by multiple species delimitation methods, and revisited the genetic distance and topology for estimating the reliable and accurate outcomes of species delimitation threshold. Large scale barcode reference library of spiders can provide an open access gateway for researchers from different arenas of biodiversity studies such as taxonomy, ecology, and behaviour etc. Hence, this integrated approach by both morphology and DNA barcoding, evidenced as a useful approach for spider identification, detection of cryptic species, identify the species complexes, and description of the reinstatement of original combination. The current study also enriches the global database with DNA barcode data of Indian spiders and valuable information on the genetic diversity and delimitation threshold. The present effort also provides new insights to the taxonomic research on spiders from India by contributing novel barcodes to the global database, cryptic species detection indicating possible new species, and reinstates the original combination of *Linyphia sikkimensis*. The aimed study with the contemporary methodological approach is not only help to the arachnological community, but also useful to the broader readers associated with the biodiversity and systematics research.

## Supplementary information

SUPPLEMENTARY INFO
