# Supplementary material for: Identification of Indian Spiders through DNA barcoding: Cryptic species and species complex
Source: Sci Rep. 2019 Oct 1;9:14033. doi: 10.1038/s41598-019-50510-8 (PMC6773733; doi:10.1038/s41598-019-50510-8)
Supplement: Supplementary file 1 — SUPPLEMENTARY INFO [file 41598_2019_50510_MOESM1_ESM.docx]

**Identification of Indian Spiders through DNA barcoding: Cryptic species and species complex**

Kaomud Tyagi, Vikas Kumar*, Shantanu Kundu, Avas Pakrashi, Priya Prasad, John TD Caleb, Kailash Chandra

Centre for DNA Taxonomy, Molecular Systematics Division, Zoological Survey of India, M- Block, New Alipore, Kolkata, 700 053, West Bengal, India.

***Corresponding author: Vikas Kumar (email:vikaszsi77@gmail.com)Fig S1: Sampling sites of the studied spider species in six different states of India. Map not to scale.**


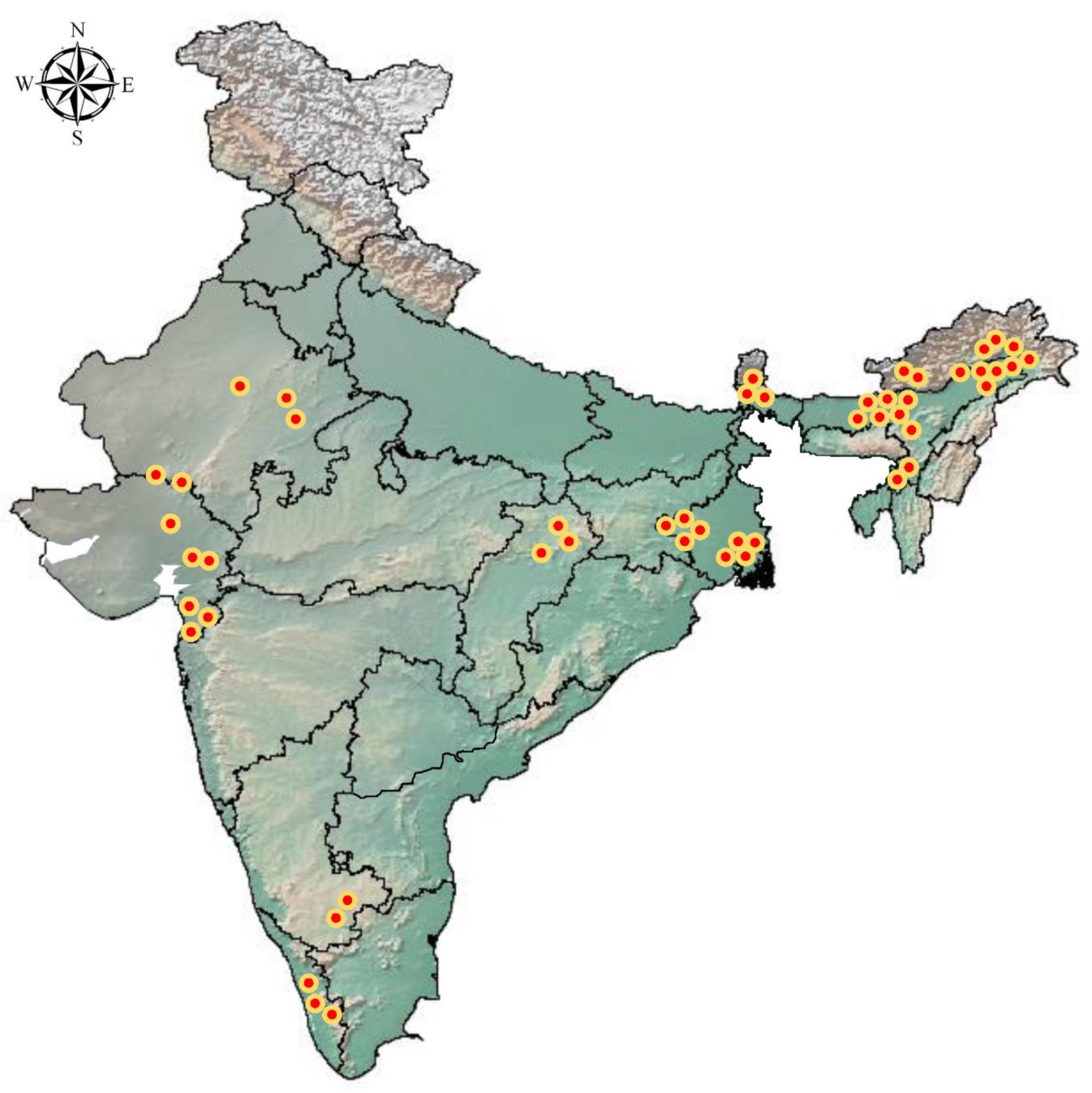


**Fig S2: Dorsal view of all the voucher specimens including identified species and unidentified species up to genus level.** Name of the species is given against the Arabic numerical. The specimens were collected by K.T., S.K., A.P., K.C. and the photographs were taken by J.T.D.C., P.P., A.P. Species names are: (1) *Araneus mitificus*; (2) *Argiope versicolor*; (3) *Argiope pulchella*; (4) *Cyclosa bianchoria*; (5) *Cyclosa mulmeinensis*; (6) *Cyclosa bifida*; (7) *Cyclosa spirifera*; (8) *Cyrtophora cicatrosa;* (9) *Chorizopes quadrituberculata*; (10) *Eriovixia poonaensis*; (11) *Eriovixia excelsa*; (12) *Gasteracantha diadesmia*; (13)*Gasteracantha hasselti*; (14) *Gasteracantha kuhli*; (15) *Neoscona nautica*; (16) *Neoscona theisi*; (17) *Nephila pilipes*; (18) *Nephilengys malabarensis*; (19) *Parawixia dehaani*; (20) *Clubiona* sp.1; (21) *Deinopis* sp.; (22) *Cheiracanthium triviale*; (23) *Hersilia savignyi*; (24) *Linyphia sikkimensis* comb. rev.; (25) *Draposa* sp.; (26) *Hogna himalayensis*; (27) *Lycosa nigrotibialis*; (28) *Pardosa birmanica*; (29) *Pardosa pusiola*; (30) *Pardosa sumatrana*; (31) *Damarchus* sp.; (32) *Oecobius putus*; (33) *Hamataliwa* sp1.; (34) *Oxyopes birmanicus*; (35) *Oxyopes shweta*; (36) *Oxyopes javanus*; (37) *Oxyopes hindostanicus*; (38) *Artema atlanta*; (39) *Crossopriza lyoni*; (40) *Micropholcus fauroti*; (41) *Fecenia protensa*; (42) *Psechrus inflatus*; (43) *Bavia* sp.; (44) *Carrhotus* sp.; (45) *Evarcha flavocincta*; (46) *Epocilla sirohi*; (47) *Hasarius adansoni*; (48) *Menemerus nigli*; (49) *Menemerus bivittatus*; (50) *Mogrus rajasthanensis*; (51) *Myrmarachne kiboschensis*; (52) *Pancorius magnus*; (53) *Phintella accentifera*; (54) *Phintella vittata*; (55) *Phintella versicolor*; (56) *Plexippus petersi*; (57) *Plexippus paykulli*; (58) *Rhene flavicomans*; (59) *Siler semiglaucus*; (60) *Thiania bhamoensis*; (61) *Telamonia dimidiata*; (62) *Yaginumaella incognita*; (63) *Heteropoda venatoria*; (64) *Pseudopoda cheppe*; (65) *Sinopoda* sp.; (66) *Scytodes fusca*; (67) *Guizygiella* sp.; (68) *Leucauge xiaoen*; (69) *Tylorida ventralis*; (70) *Leucauge tessellata*; (71) *Opadometa fastigata*; (72) *Orsinome vethi;* (73) *Tetragnatha mandibulata*; (74) *Tetragnatha hasselti*; (75) *Tetragnatha vermiformis*; (76) *Leucauge celebesiana*; (77) *Tylorida striata*; (78) *Lyrognathus crotalus*; (79) *Argyrodes flavescens*; (80) *Argyrodes projeles*; (81) *Ariamnes simulans*; (82) *Thwaitesia* sp.; (83) *Pardosa procurva* (84) *Nesticodes rufipes*; (85) *Nihonhimea mundula*; (86) *Thomisus unidentatus*; (87) *Indoxysticus* sp.; (88) *Synema* sp.; (89) *Camaricus formosus;* (90) *Uloborus* sp.; (91) *Zosis geniculata*; (92) *Mallinella* sp; (93) *Cyclosa quinqueguttata*; (94) *Chalcotropis pennata*; (95) *Hamataliwa* sp2.; (96) *Clubiona* sp2. (97) *Oxyopes sakuntalae*; (98) *Meotipa sahyadri*; (99) *Scytodes thoracica*; (100) *Pardosa pseudoannulata*; (101) *Epidius parvati*.

**
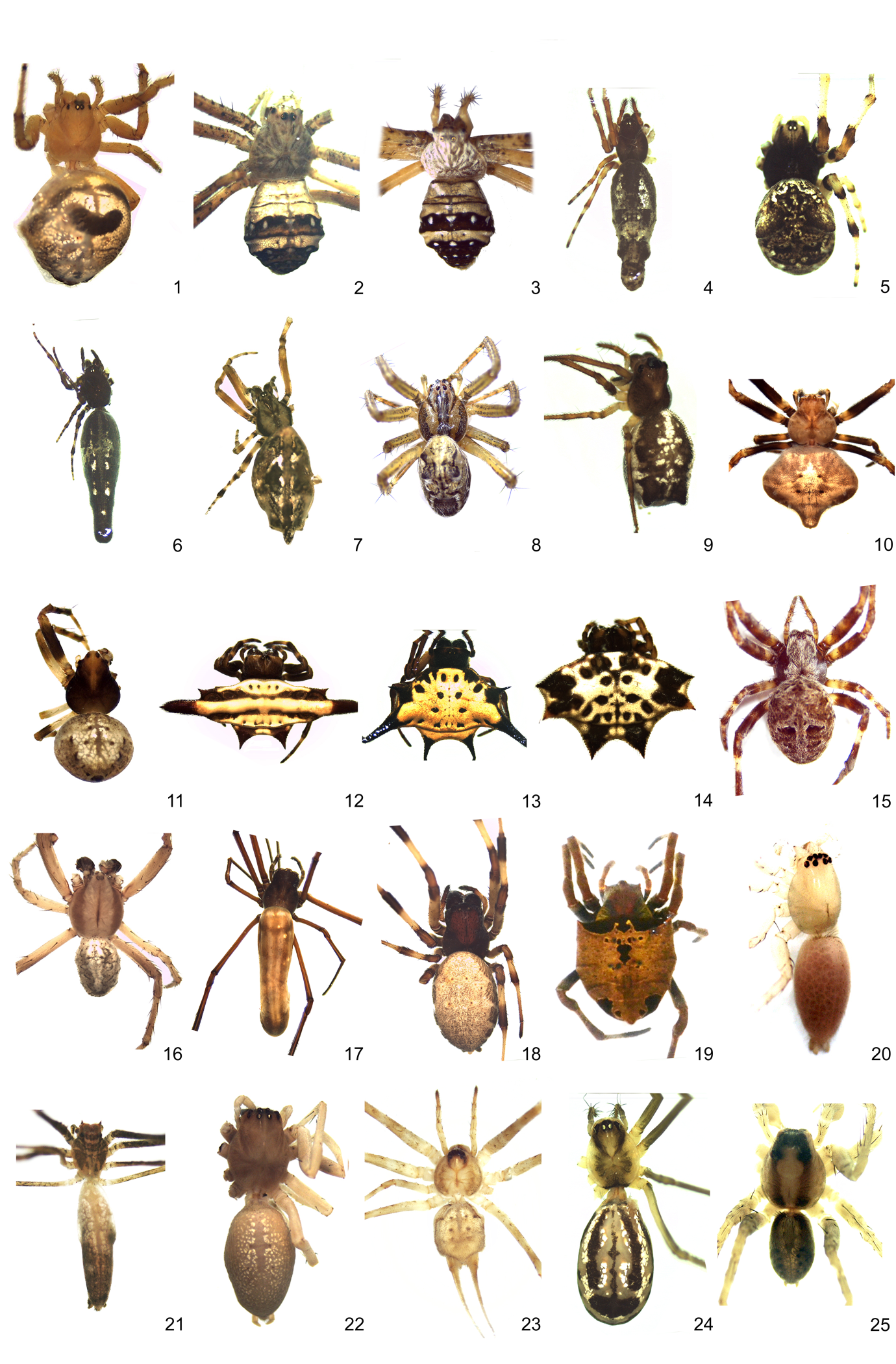
**

**
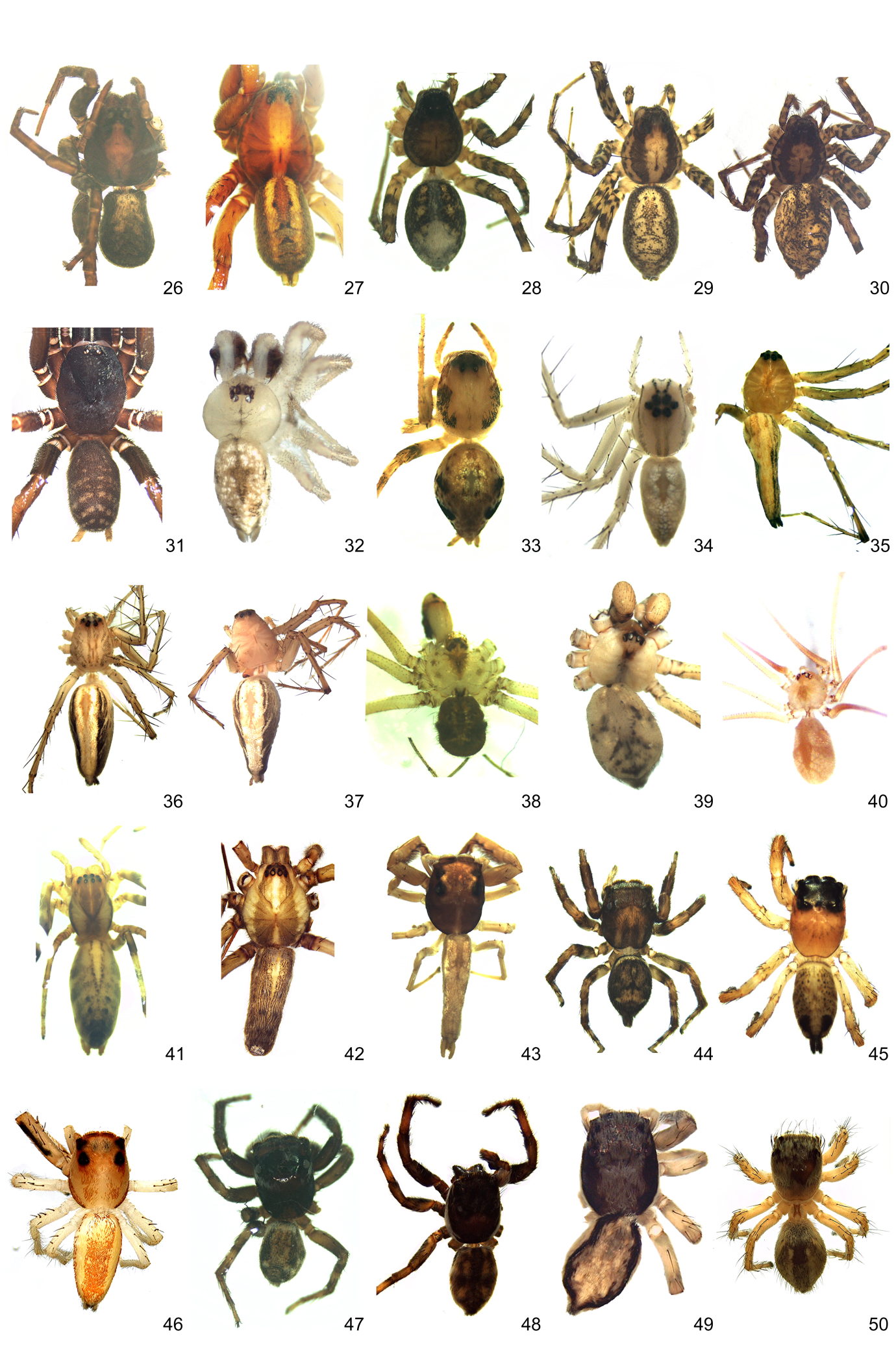
** **
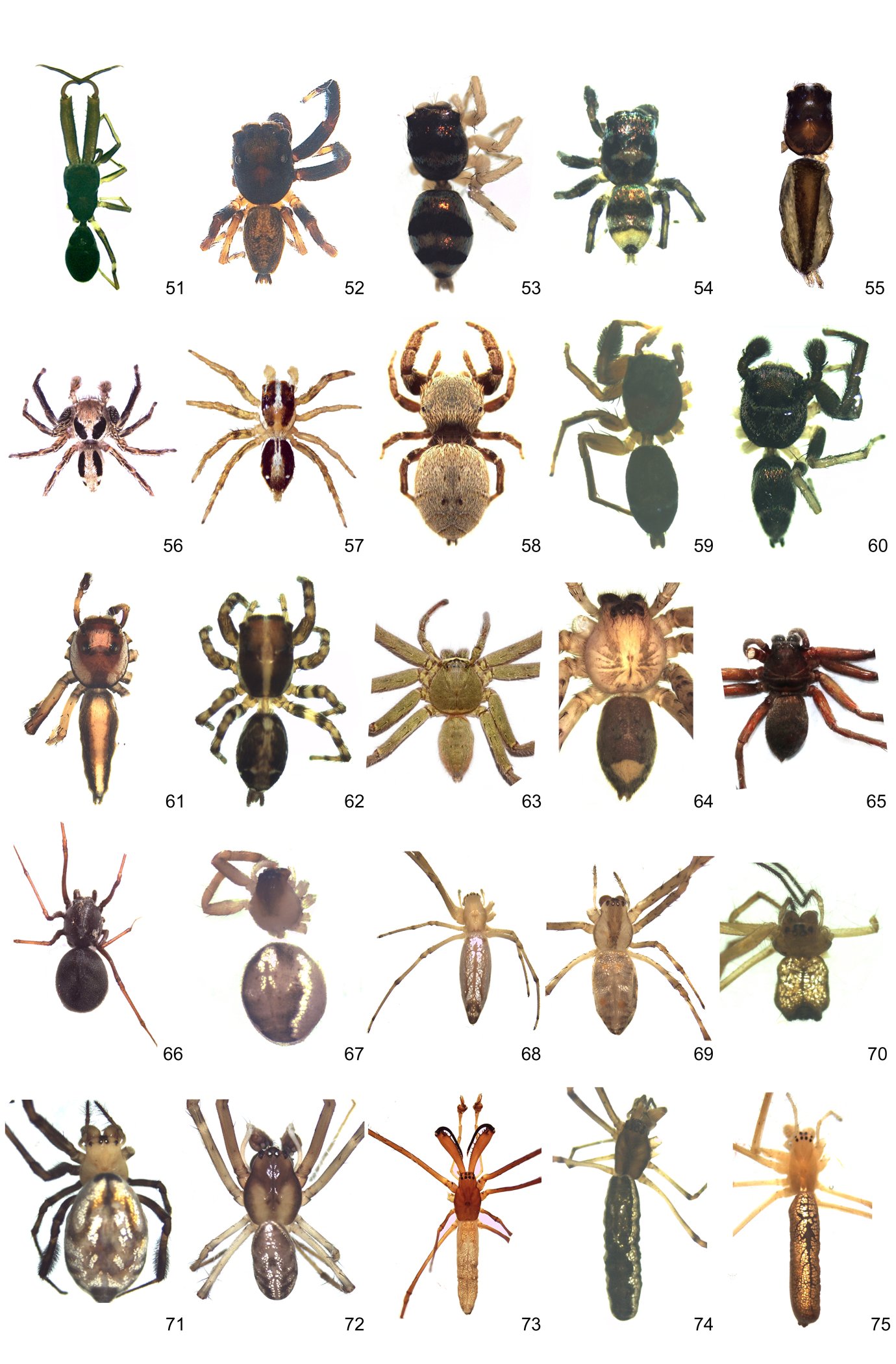
**

**
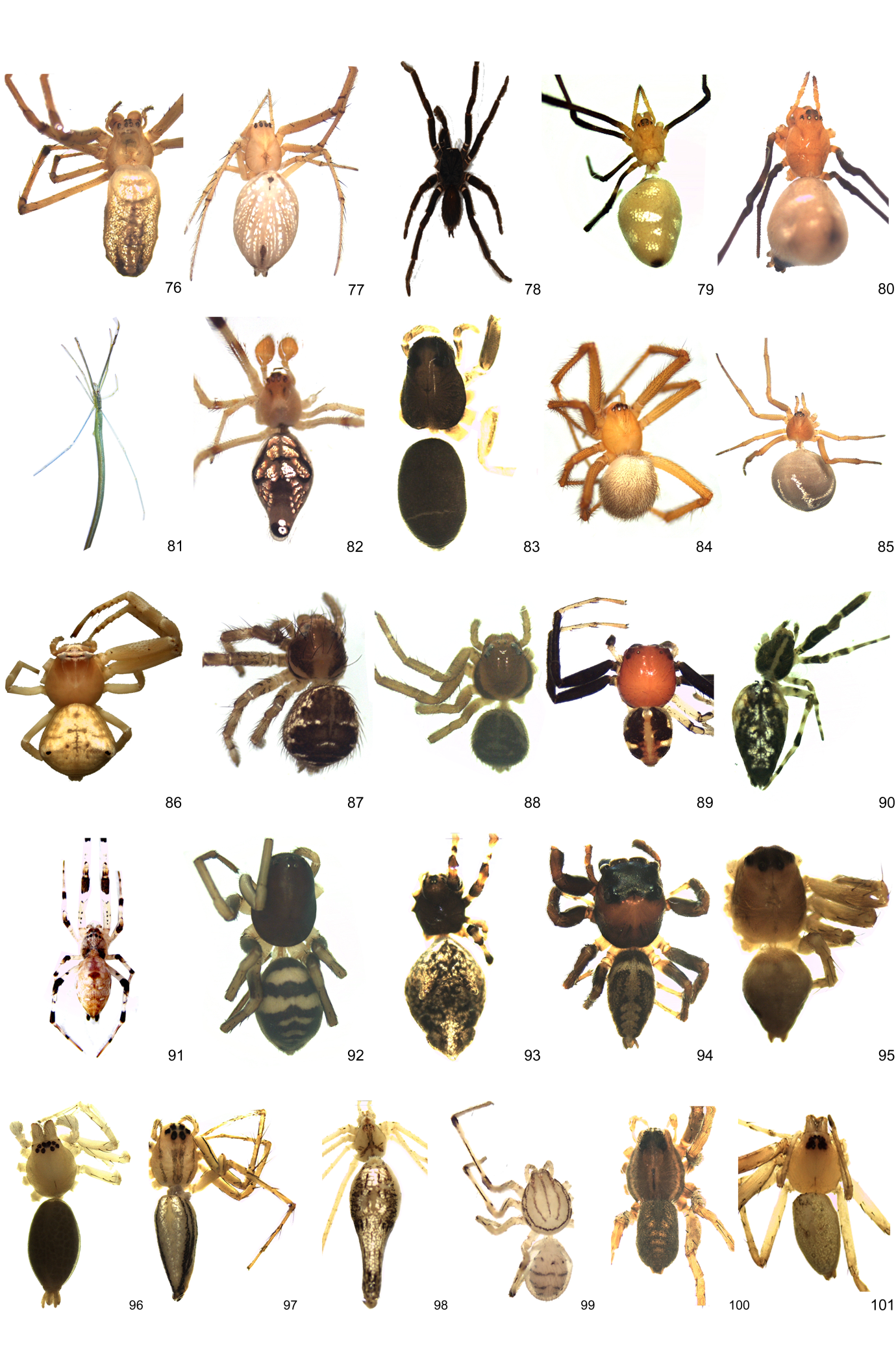
**

**Fig S3: The enlarged photographs of the genitalia morphology of the species represented in each respective clades of the pruned BA trees (Figures 3-6**). Name of the species is given against the Arabic numerical. The photographs were taken by J.T.D.C., P.P., A.P. (1) *Cyclosa spirifera* epigynal scape dorsal view, (2) *Cyclosa spirifera* epigynal scape dorsal view, (3) *Pardosa pusiola* epigyne ventral view, (4) *Pardosa pusiola* epigyne dorsal view, (5) *Pardosa pusiola* epigyne ventral view, (6) *Pardosa pusiola* epigyne dorsal view, (7) *Heteropoda venatoria* epigyne ventral view, (8) *Heteropoda venatoria* epigyne dorsal view, (9) *Heteropoda venatoria* male palp ventral view, (10) *Heteropoda venatoria* male palp lateral view, (11) *Thiania bhamoensis* epigyne ventral view, (12) *Thiania bhamoensis* epigyne dorsal view, (13) *Thiania bhamoensis* male palp ventral view, (14) *Thiania bhamoensis* male palp lateral view, (15) *Pardosa sumatrana* epigyne ventral view, (16) *Pardosa sumatrana* epigyne dorsal view, (17) *Pardosa sumatrana* epigyne ventral view, (18) *Pardosa sumatrana* epigyne dorsal view, (19) *Cheiracanthium triviale* epigyne ventral view, (20) *Cheiracanthium triviale* epigyne dorsal view, (21) *Cheiracanthium triviale* epigyne ventral view, (22) *Cheiracanthium triviale* epigyne dorsal view, (23) *Cyrtophora cicatrosa* epigyne ventral view, (24) *Cyrtophora cicatrosa* epigyne dorsal view, (25) *Hersilia savignyi* epigyne ventral view, (26) *Argiope versicolor* epigyne ventral view, (27) *Phintella vittata* male palp ventral view, (28) *Phintella vittata* male palp lateral view, (29) *Phintella vittata* epigyne ventral view, (30) *Phintella vittata* epigyne dorsal view, (31) *Oxyopes birmanicus* epigyne ventral view, (32) *Oxyopes birmanicus* male palp ventral view, (33) *Plexippus paykulli* epigyne ventral view, (34) *Plexippus paykulli* epigyne dorsal view, (35) *Plexippus paykulli* male palp ventral view, (36) *Plexippus paykulli* male palp lateral view, (37) *Plexippus petersi* male palp ventral view, (38) *Plexippus petersi* male palp lateral view, (39) *Plexippus petersi* epigyne ventral view, (40) *Plexippus petersi* epigyne dorsal view, (41) *Linyphia sikkimensis* epigyne ventral view, (42) *Linyphia sikkimensis* epigyne dorsal view.

**
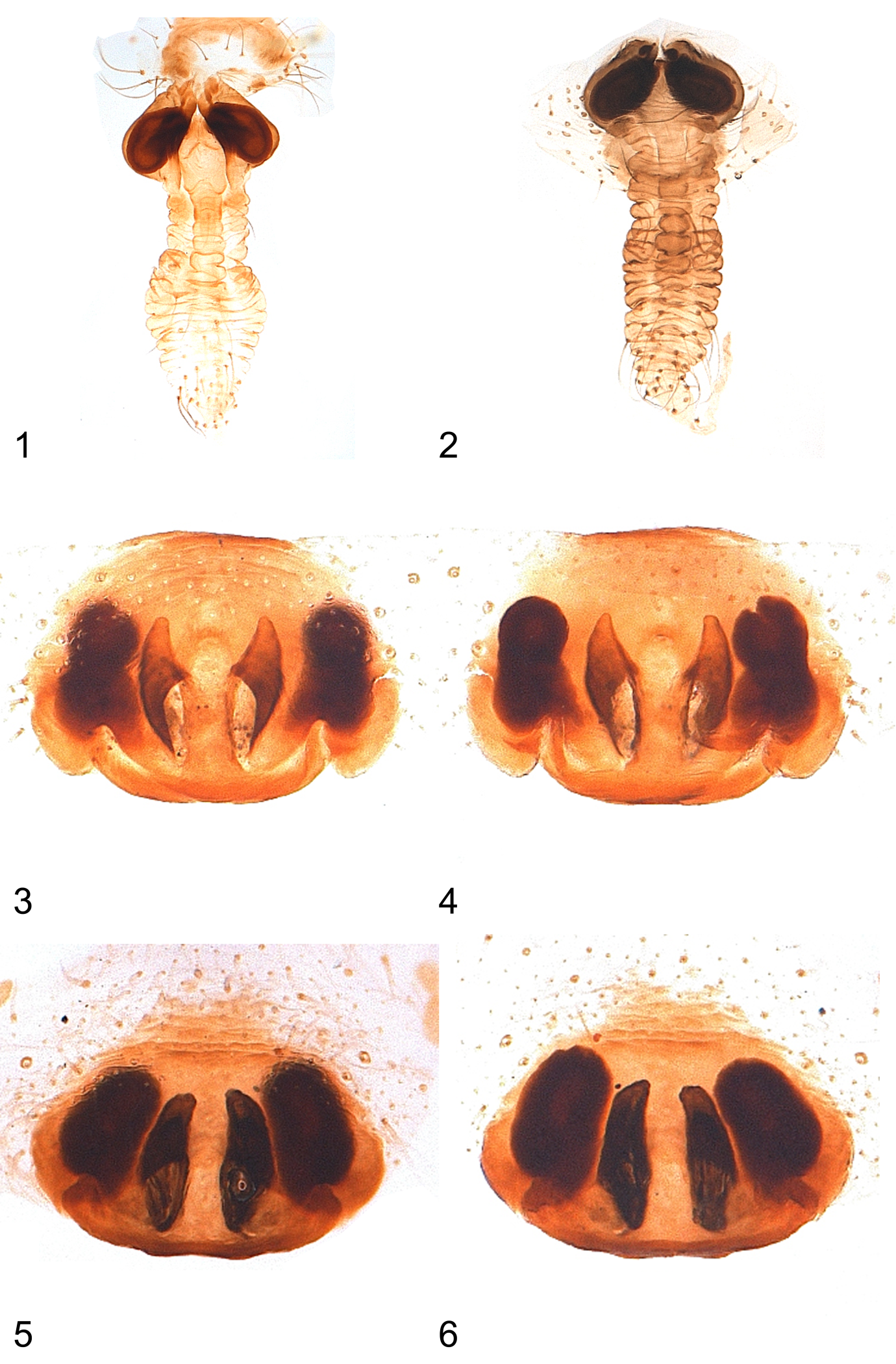
**

**
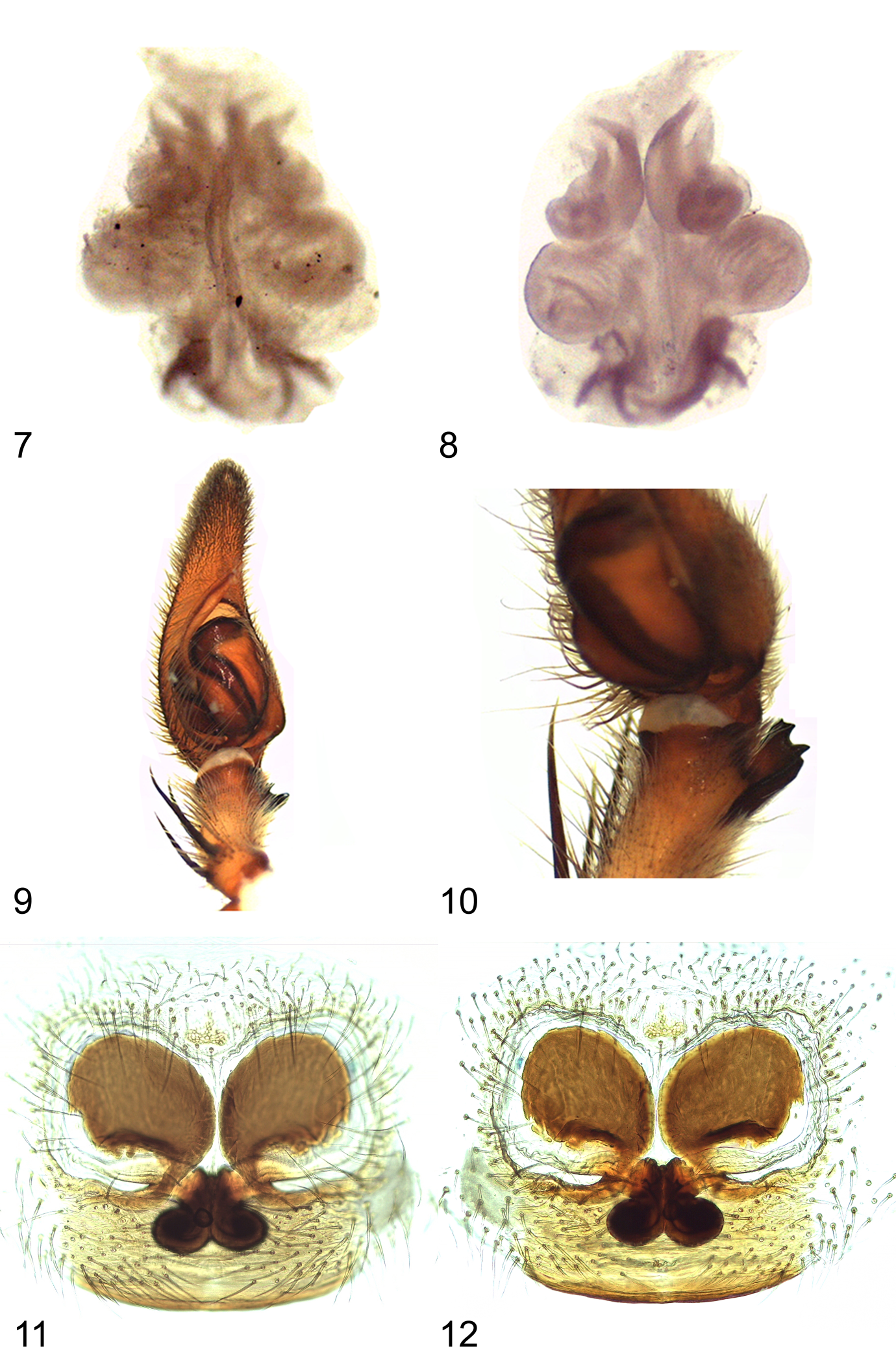
** **
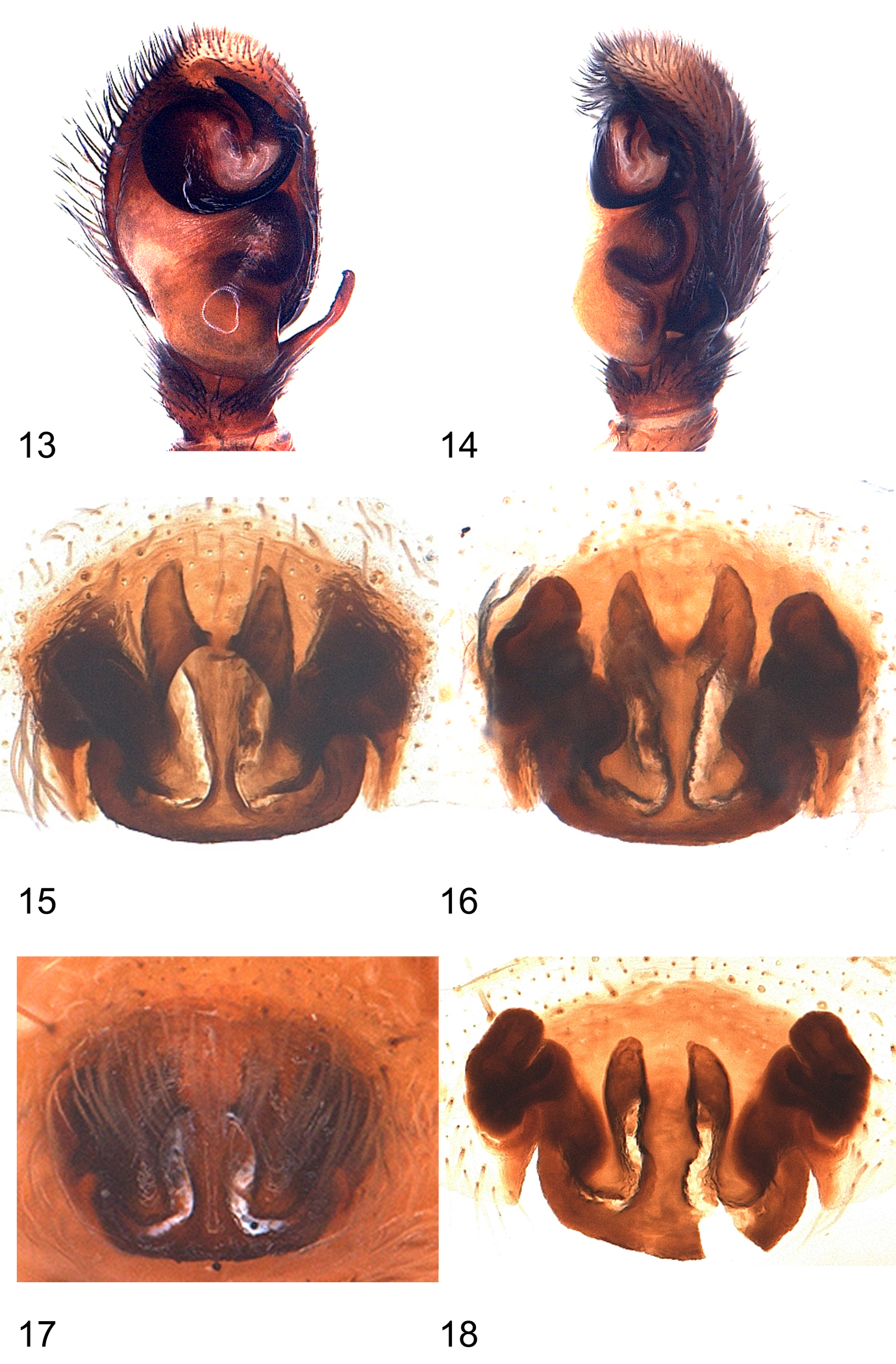

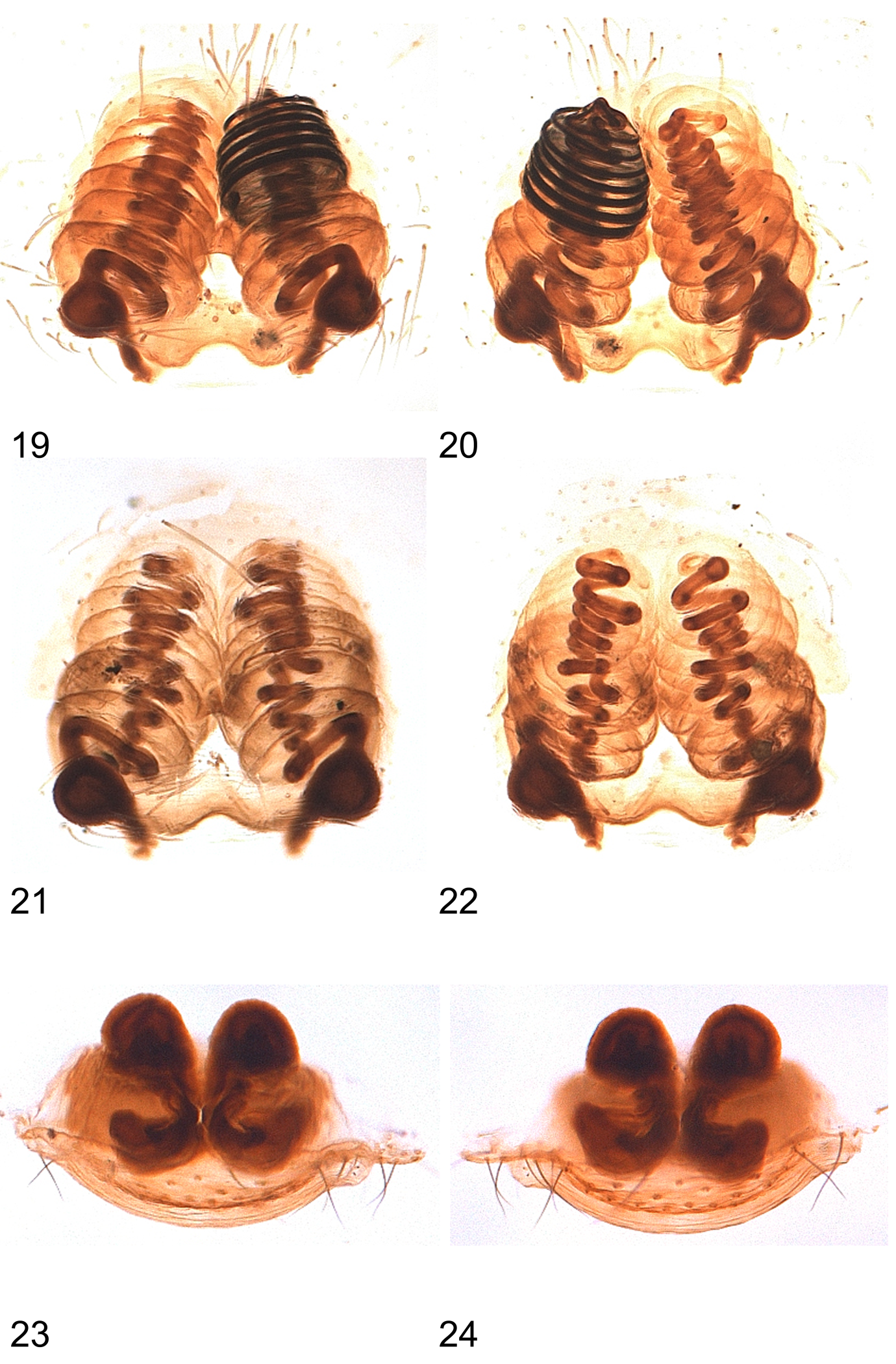
**

**
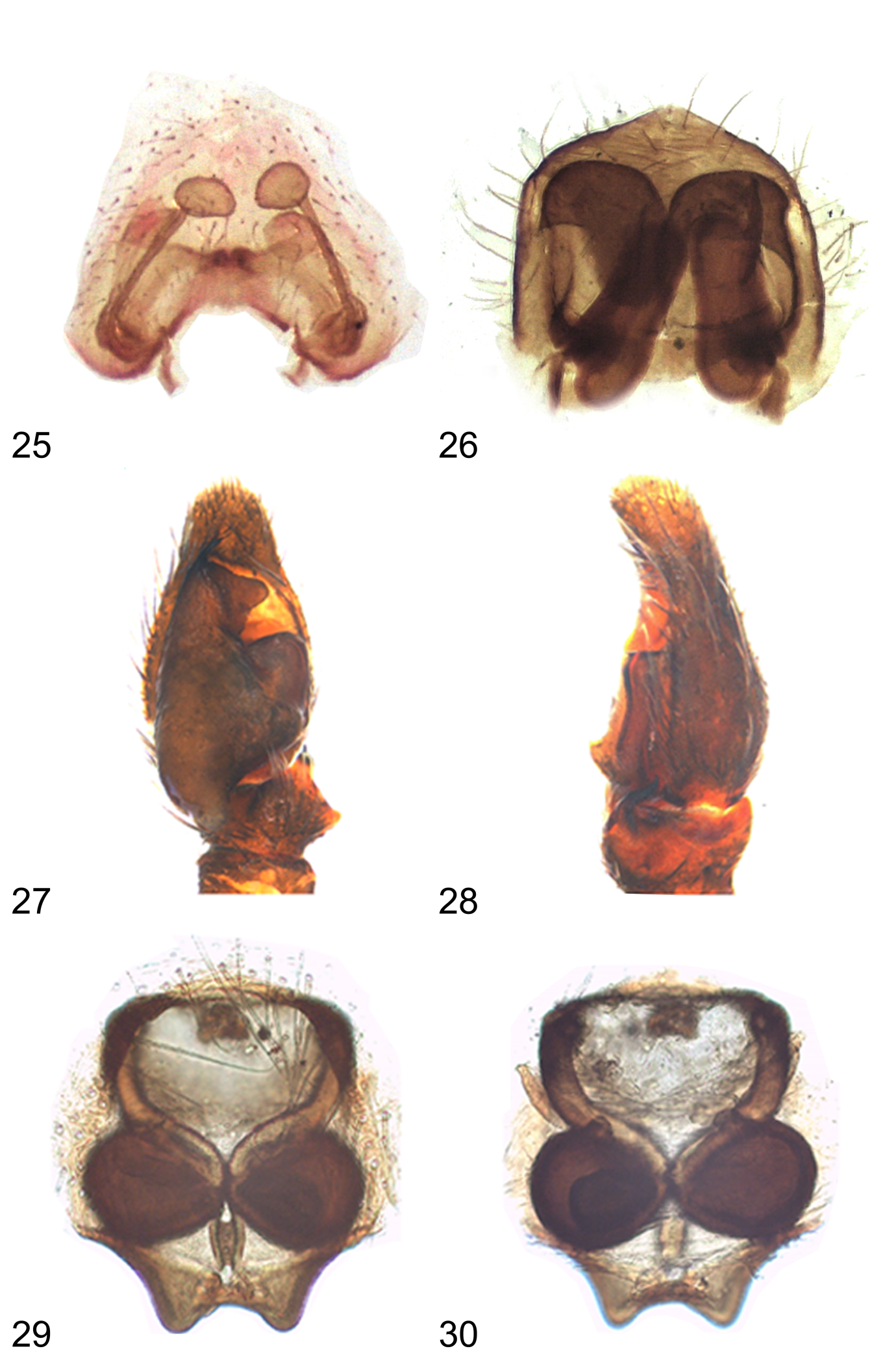
**

**
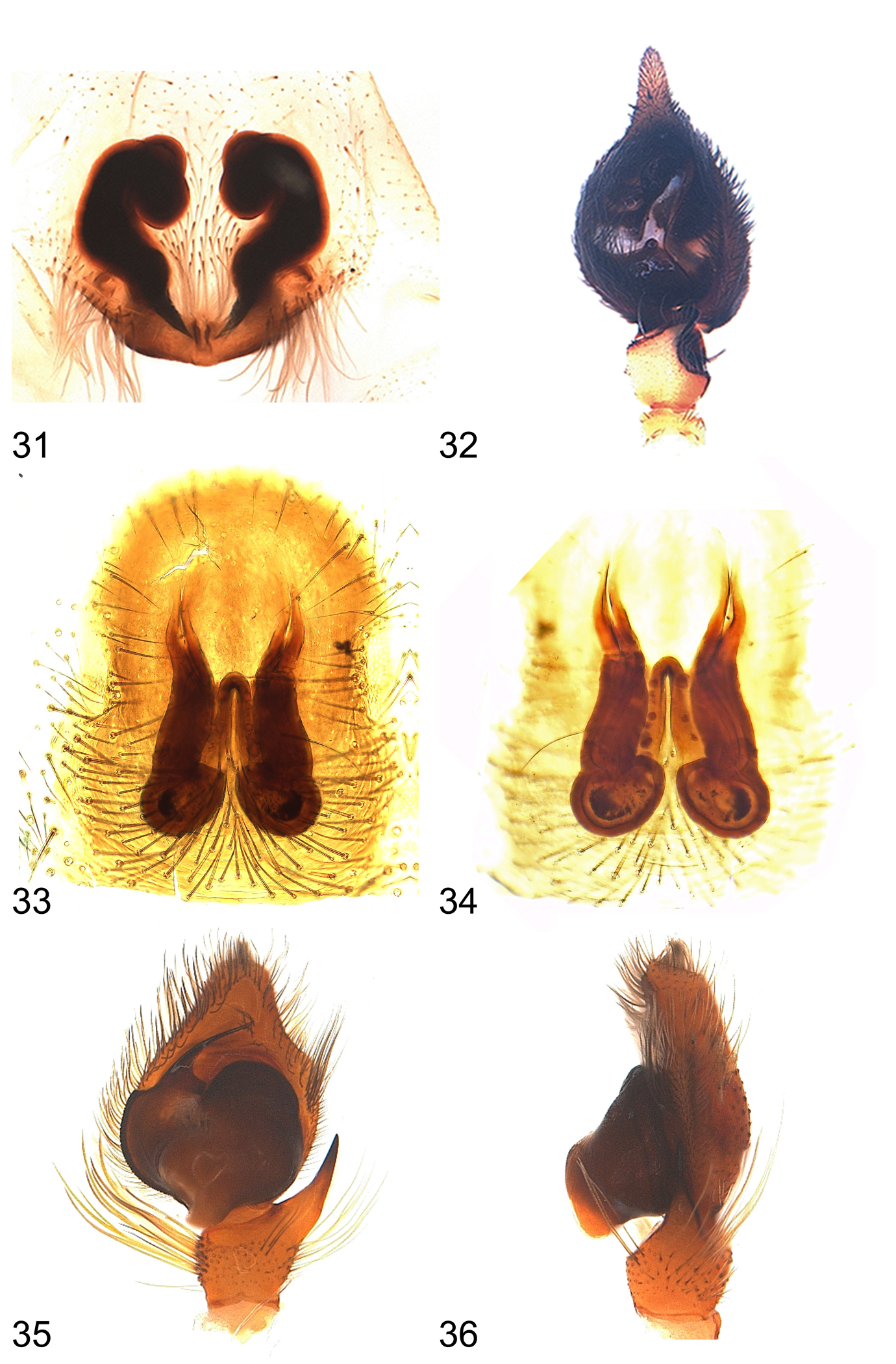
** **
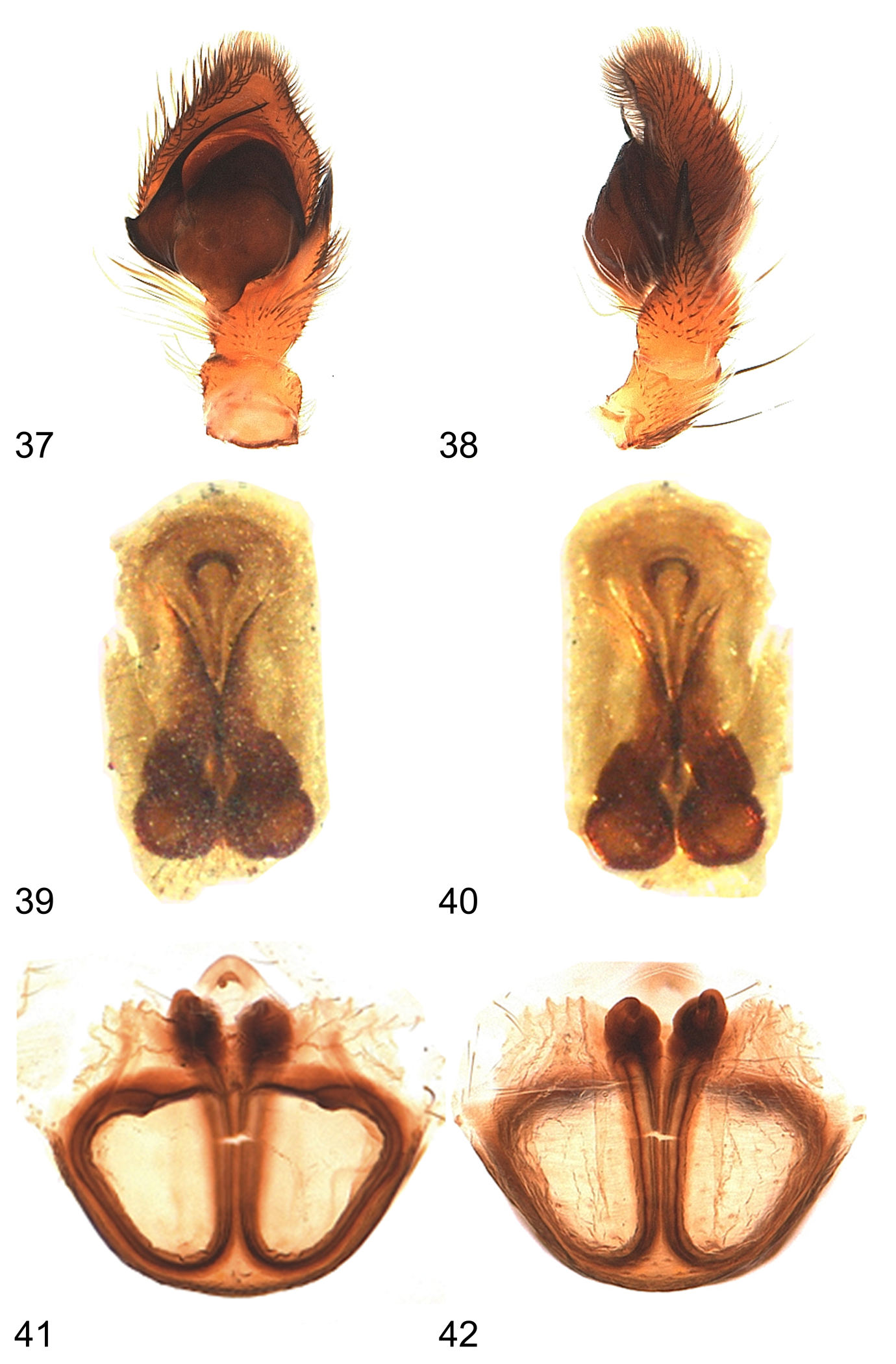
**

**Fig S4: Neighbor-Joining tree of the 489 studied specimens with 1000 bootstrap support.**

**
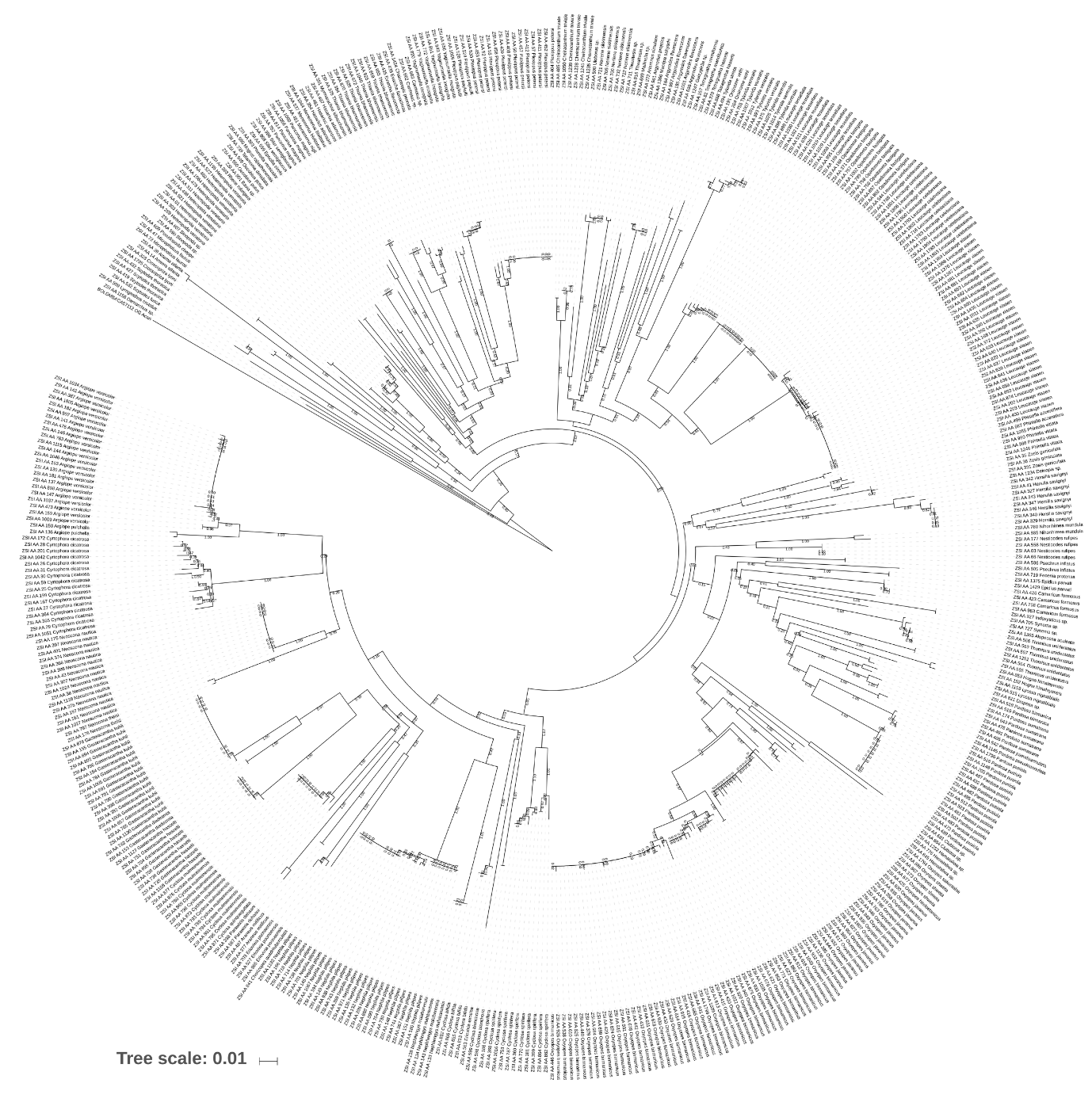
**


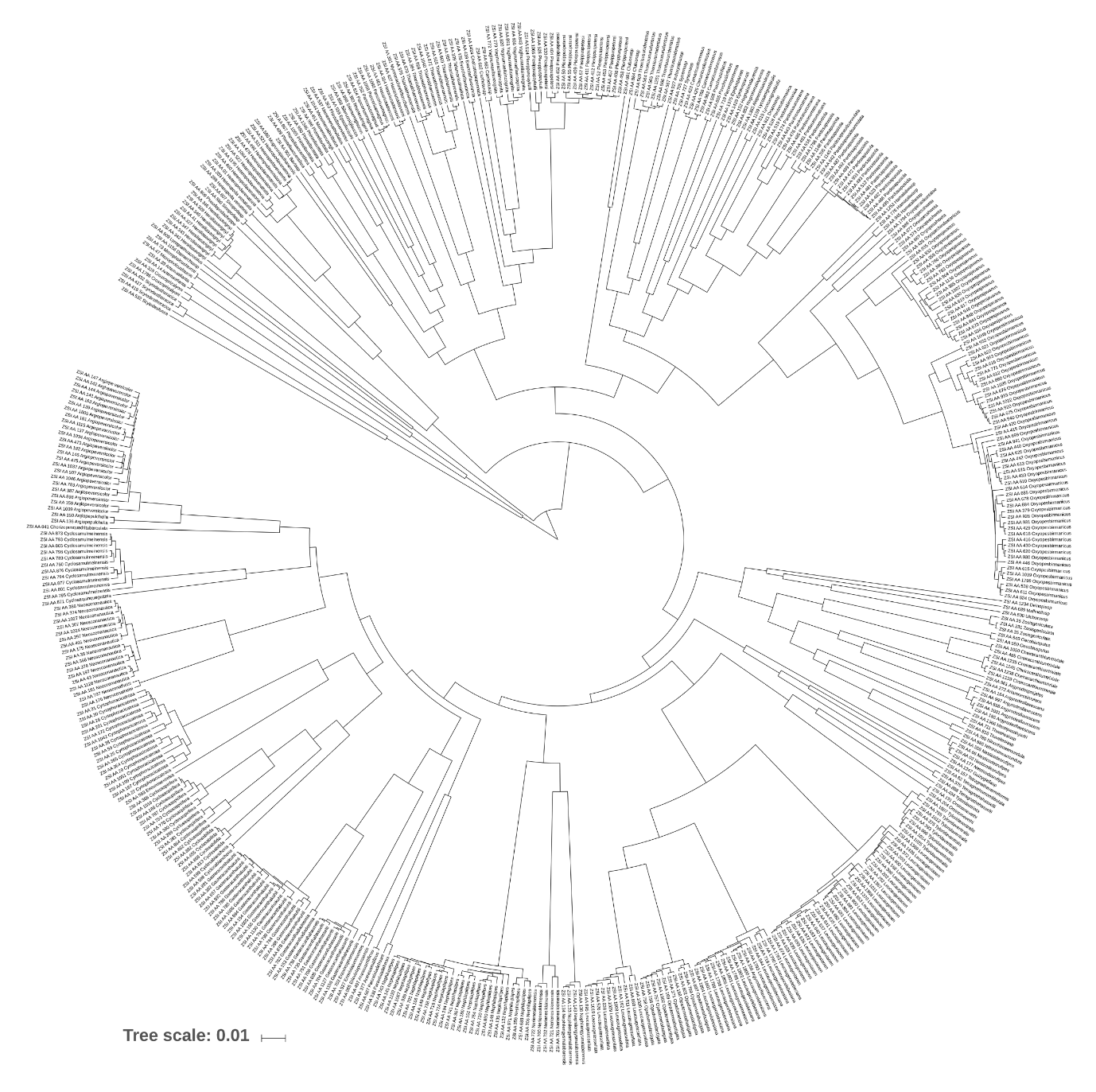
**Fig S5: Ultra-metric maximum likelihood (ML) tree generated in BEAST with generated 489 DNA barcodes for GMYC analysis.**

**Table S1: Sample ID, Voucher ID, Family, Species, Collection coordinates, BOLD Process ID, and GenBank accession no. of the studied specimens.**

| **SL. No.** | **Sample ID** | **Voucher ID** | **Family** | **Species** | **Lat_Lon** | **BOLD Process ID** | **GenBank Accession No.** | **Haplotype** |
| --- | --- | --- | --- | --- | --- | --- | --- | --- |
| 1 | AA-377 | ZSI_AA-377 | Araneidae | *Araneus mitificus* | 26.67 N 92.85 E | BOLD:ADF2585 | MK392634 | Hap_1 |
| 2 | AA-497 | ZSI_AA-497 | Araneidae | *Araneus mitificus* | 26.20 N 92.93 E | BOLD:ADF2585 | MK392635 | Hap_1 |
| 3 | AA-147 | ZSI_AA-147 | Araneidae | *Argiope versicolor* | 26.71 N 92.81 E | BOLD:ACG0576 | MK392636 | Hap_2 |
| 4 | AA-137 | ZSI_AA-137 | Araneidae | *Argiope versicolor* | 26.20 N 92.93 E | BOLD:ACG0576 | MK392637 | Hap_3 |
| 5 | AA-181 | ZSI_AA-181 | Araneidae | *Argiope versicolor* | 26.71 N 92.81 E | BOLD:ACG0576 | MK392638 | Hap_3 |
| 6 | AA-139 | ZSI_AA-139 | Araneidae | *Argiope versicolor* | 26.66 N 92.86 E | BOLD:ACG0576 | MK392639 | Hap_3 |
| 7 | AA-163 | ZSI_AA-163 | Araneidae | *Argiope versicolor* | 26.71 N 92.81 E | BOLD:ACG0576 | MK392640 | Hap_3 |
| 8 | AA-1046 | ZSI_AA-1046 | Araneidae | *Argiope versicolor* | 24.86 N 92.56 E | BOLD:ACG0576 | MK392641 | Hap_3 |
| 9 | AA-144 | ZSI_AA-144 | Araneidae | *Argiope versicolor* | 26.66 N 92.86 E | BOLD:ACG0576 | MK392642 | Hap_3 |
| 10 | AA-1115 | ZSI_AA-1115 | Araneidae | *Argiope versicolor* | 21.00 N 72.85 E | BOLD:ACG0576 | MK392643 | Hap_3 |
| 11 | AA-783 | ZSI_AA-783 | Araneidae | *Argiope versicolor* | 27.66 N 95.36 E | BOLD:ACG0576 | MK392644 | Hap_3 |
| 12 | AA-142 | ZSI_AA-142 | Araneidae | *Argiope versicolor* | 26.66 N 92.86 E | BOLD:ACG0576 | MK392645 | Hap_4 |
| 13 | AA-1034 | ZSI_AA-1034 | Araneidae | *Argiope versicolor* | 24.86 N 92.56 E | BOLD:ACG0576 | MK392646 | Hap_5 |
| 14 | AA-1009 | ZSI_AA-1009 | Araneidae | *Argiope versicolor* | 27.57 N 95.39 E | BOLD:ACG0576 | MK392647 | Hap_6 |
| 15 | AA-146 | ZSI_AA-146 | Araneidae | *Argiope versicolor* | 26.71 N 92.81 E | BOLD:ACG0576 | MK392648 | Hap_3 |
| 16 | AA-475 | ZSI_AA-475 | Araneidae | *Argiope versicolor* | 26.20 N 92.93 E | BOLD:ACG0576 | MK392649 | Hap_3 |
| 17 | AA-141 | ZSI_AA-141 | Araneidae | *Argiope versicolor* | 26.66 N 92.86 E | BOLD:ACG0576 | MK392650 | Hap_3 |
| 18 | AA-387 | ZSI_AA-387 | Araneidae | *Argiope versicolor* | 26.67 N 92.85 E | BOLD:ACG0576 | MK392651 | Hap_7 |
| 19 | AA-1037 | ZSI_AA-1037 | Araneidae | *Argiope versicolor* | 24.86 N 92.56 E | BOLD:ACG0576 | MK392652 | Hap_8 |
| 20 | AA-507 | ZSI_AA-507 | Araneidae | *Argiope versicolor* | 26.20 N 92.93 E | BOLD:ACG0576 | MK392653 | Hap_3 |
| 21 | AA-473 | ZSI_AA-473 | Araneidae | *Argiope versicolor* | 26.20 N 92.93 E | BOLD:ACG0576 | MK392654 | Hap_9 |
| 22 | AA-898 | ZSI_AA-898 | Araneidae | *Argiope versicolor* | 26.81 N 93.31 E | BOLD:ACG0576 | MK392655 | Hap_10 |
| 23 | AA-182 | ZSI_AA-182 | Araneidae | *Argiope versicolor* | 26.71 N 92.81 E | BOLD:ACG0576 | MK392656 | Hap_3 |
| 24 | AA-159 | ZSI_AA-159 | Araneidae | *Argiope versicolor* | 24.86 N 92.56 E | BOLD:ACG0576 | MK392657 | Hap_6 |
| 25 | AA-1805 | ZSI_AA-774 | Araneidae | *Argiope versicolor* | 23.22 N 72.67 E | BOLD:ACG0576 | MK392658 | Hap_11 |
| 26 | AA-136 | ZSI_AA-136 | Araneidae | *Argiope pulchella* | 22.16 N 88.82 E | BOLD:ADA1817 | MK392659 | Hap_12 |
| 27 | AA-150 | ZSI_AA-150 | Araneidae | *Argiope pulchella* | 22.55 N 88.29 E | BOLD:ADA1817 | MK392660 | Hap_13 |
| 28 | AA-655 | ZSI_AA-655 | Araneidae | *Cyclosa bifida* | 27.52 N 95.36 E | BOLD:ADI4161 | MK392661 | Hap_14 |
| 29 | AA-866 | ZSI_AA-866 | Araneidae | *Cyclosa bifida* | 27.57 N 95.33 E | BOLD:ADI4161 | MK392662 | Hap_14 |
| 30 | AA-813 | ZSI_AA-813 | Araneidae | *Cyclosa bifida* | 27.66 N 95.36 E | BOLD:ADI4161 | MK392663 | Hap_15 |
| 31 | AA-882 | ZSI_AA-882 | Araneidae | *Cyclosa bifida* | 27.57 N 95.33 E | BOLD:ADI4161 | MK392664 | Hap_14 |
| 32 | AA-598 | ZSI_AA-598 | Araneidae | *Cyclosa bianchoria* | 28.60 N 95.49 E | BOLD:ADG9174 | MK392665 | Hap_16 |
| 33 | AA-599 | ZSI_AA-599 | Araneidae | *Cyclosa bianchoria* | 28.60 N 95.49 E | BOLD:ADG9174 | MK392666 | Hap_16 |
| 34 | AA-1016 | ZSI_AA-1016 | Araneidae | *Cyclosa spirifera* | 27.57 N 95.39 E | BOLD:ADF5040 | MK392667 | Hap_17 |
| 35 | AA-753 | ZSI_AA-753 | Araneidae | *Cyclosa spirifera* | 27.57 N 95.39 E | BOLD:ADF5040 | MK392668 | Hap_18 |
| 36 | AA-770 | ZSI_AA-770 | Araneidae | *Cyclosa spirifera* | 27.57 N 95.39 E | BOLD:ADF5040 | MK392669 | Hap_19 |
| 37 | AA-399 | ZSI_AA-399 | Araneidae | *Cyclosa spirifera* | 26.67 N 92.85 E | BOLD:ADF5039 | MK392670 | Hap_20 |
| 38 | AA-381 | ZSI_AA-381 | Araneidae | *Cyclosa spirifera* | 26.67 N 92.85 E | BOLD:ADF5039 | MK392671 | Hap_20 |
| 39 | AA-864 | ZSI_AA-864 | Araneidae | *Cyclosa spirifera* | 26.67 N 92.85 E | BOLD:ADF5039 | MK392672 | Hap_21 |
| 40 | AA-368 | ZSI_AA-368 | Araneidae | *Cyclosa spirifera* | 27.47 N 94.91 E | BOLD:ADF5040 | MK392673 | Hap_17 |
| 41 | AA-862 | ZSI_AA-862 | Araneidae | *Cyclosa spirifera* | 26.67 N 92.85 E | BOLD:ADF5039 | MK392674 | Hap_22 |
| 42 | AA-186 | ZSI_AA-186 | Araneidae | *Cyclosa spirifera* | 27.57 N 95.39 E | BOLD:ADF5040 | MK392675 | Hap_17 |
| 43 | AA-787 | ZSI_AA-787 | Araneidae | *Cyclosa spirifera* | 27.47 N 94.91 E | BOLD:ADF5040 | MK392676 | Hap_23 |
| 44 | AA-360 | ZSI_AA-360 | Araneidae | *Cyclosa spirifera* | 27.47 N 94.91 E | BOLD:ADF5040 | MK392677 | Hap_24 |
| 45 | AA-1027 | ZSI_AA-1027 | Araneidae | *Neoscona nautica* | 27.57 N 95.39 E | BOLD:ACI2573 | MK392678 | Hap_25 |
| 46 | AA-161 | ZSI_AA-161 | Araneidae | *Neoscona nautica* | 22.37 N 73.17 E | BOLD:ACI2573 | MK392679 | Hap_25 |
| 47 | AA-197 | ZSI_AA-197 | Araneidae | *Neoscona nautica* | 22.39 N 86.94 E | BOLD:ACI2573 | MK392680 | Hap_25 |
| 48 | AA-376 | ZSI_AA-376 | Araneidae | *Neoscona nautica* | 26.67 N 92.85 E | BOLD:ACI2573 | MK392681 | Hap_25 |
| 49 | AA-1128 | ZSI_AA-1128 | Araneidae | *Neoscona nautica* | 20.76 N 73.37 E | BOLD:ADL9949 | MK392682 | Hap_25 |
| 50 | AA-38 | ZSI_AA-38 | Araneidae | *Neoscona nautica* | 23.41 N 87.11 E | BOLD:ACI2573 | MK392683 | Hap_25 |
| 51 | AA-1024 | ZSI_AA-1024 | Araneidae | *Neoscona nautica* | 27.57 N 95.39 E | BOLD:ACI2573 | MK392684 | Hap_25 |
| 52 | AA-307 | ZSI_AA-307 | Araneidae | *Neoscona nautica* | 22.12 N 88.73 E | BOLD:ACI2573 | MK392685 | Hap_25 |
| 53 | AA-43 | ZSI_AA-43 | Araneidae | *Neoscona nautica* | 23.41 N 87.11 E | BOLD:ACI2573 | MK392686 | Hap_25 |
| 54 | AA-386 | ZSI_AA-386 | Araneidae | *Neoscona nautica* | 26.67 N 92.85 E | BOLD:ACI2573 | MK392687 | Hap_25 |
| 55 | AA-366 | ZSI_AA-366 | Araneidae | *Neoscona nautica* | 27.47 N 94.91 E | BOLD:ACI2573 | MK392688 | Hap_25 |
| 56 | AA-374 | ZSI_AA-374 | Araneidae | *Neoscona nautica* | 26.67 N 92.85 E | BOLD:ACI2573 | MK392689 | Hap_25 |
| 57 | AA-401 | ZSI_AA-401 | Araneidae | *Neoscona nautica* | 26.67 N 92.85 E | BOLD:ACI2573 | MK392690 | Hap_25 |
| 58 | AA-397 | ZSI_AA-397 | Araneidae | *Neoscona nautica* | 26.67 N 92.85 E | BOLD:ACI2573 | MK392691 | Hap_25 |
| 59 | AA-175 | ZSI_AA-175 | Araneidae | *Neoscona nautica* | 22.39 N 86.94 E | BOLD:ACI2573 | MK392692 | Hap_25 |
| 60 | AA-176 | ZSI_AA-176 | Araneidae | *Neoscona theisi* | 27.66 N 95.36 E | BOLD:ACM3489 | MK392693 | Hap_26 |
| 61 | AA-797 | ZSI_AA-797 | Araneidae | *Neoscona theisi* | 27.66 N 95.36 E | BOLD:ACM3489 | MK392694 | Hap_27 |
| 62 | AA-795 | ZSI_AA-795 | Araneidae | *Cyclosa mulmeinensis* | 27.66 N 95.36 E | BOLD:AAE2899 | MK392695 | Hap_28 |
| 63 | AA-876 | ZSI_AA-876 | Araneidae | *Cyclosa mulmeinensis* | 27.57 N 95.33 E | BOLD:AAE2899 | MK392696 | Hap_29 |
| 64 | AA-794 | ZSI_AA-794 | Araneidae | *Cyclosa mulmeinensis* | 27.66 N 95.36 E | BOLD:AAE2899 | MK392697 | Hap_30 |
| 65 | AA-789 | ZSI_AA-789 | Araneidae | *Cyclosa mulmeinensis* | 27.66 N 95.36 E | BOLD:AAE2899 | MK392698 | Hap_30 |
| 66 | AA-793 | ZSI_AA-793 | Araneidae | *Cyclosa mulmeinensis* | 27.66 N 95.36 E | BOLD:AAE2899 | MK392699 | Hap_30 |
| 67 | AA-873 | ZSI_AA-873 | Araneidae | *Cyclosa mulmeinensis* | 27.57 N 95.33 E | BOLD:AAE2899 | MK392700 | Hap_30 |
| 68 | AA-796 | ZSI_AA-796 | Araneidae | *Cyclosa mulmeinensis* | 27.66 N 95.36 E | BOLD:AAE2899 | MK392701 | Hap_30 |
| 69 | AA-865 | ZSI_AA-865 | Araneidae | *Cyclosa mulmeinensis* | 27.57 N 95.33 E | BOLD:AAE2899 | MK392702 | Hap_30 |
| 70 | AA-760 | ZSI_AA-760 | Araneidae | *Cyclosa mulmeinensis* | 27.57 N 95.39 E | BOLD:AAE2899 | MK392703 | Hap_30 |
| 71 | AA-877 | ZSI_AA-877 | Araneidae | *Cyclosa mulmeinensis* | 27.57 N 95.33 E | BOLD:AAE2899 | MK392704 | Hap_30 |
| 72 | AA-801 | ZSI_AA-801 | Araneidae | *Cyclosa mulmeinensis* | 27.66 N 95.36 E | BOLD:AAE2899 | MK392705 | Hap_31 |
| 73 | AA-971 | ZSI_AA-971 | Araneidae | *Nephila pilipes* | 27.52 N 95.36 E | - | MK392706 | Hap_32 |
| 74 | AA-743 | ZSI_AA-743 | Araneidae | *Nephila pilipes* | 27.29 N 95.51 E | - | MK392707 | Hap_33 |
| 75 | AA-938 | ZSI_AA-938 | Araneidae | *Nephila pilipes* | 27.52 N 95.36 E | - | MK392708 | Hap_33 |
| 76 | AA-132 | ZSI_AA-132 | Araneidae | *Nephila pilipes* | 27.47 N 94.91 E | BOLD:AAW4519 | MK392709 | Hap_34 |
| 77 | AA-149 | ZSI_AA-149 | Araneidae | *Nephila pilipes* | 27.47 N 94.91 E | BOLD:AAW4519 | MK392710 | Hap_33 |
| 78 | AA-194 | ZSI_AA-194 | Araneidae | *Nephila pilipes* | 27.63 N 95.29 E | BOLD:AAW4519 | MK392711 | Hap_33 |
| 79 | AA-135 | ZSI_AA-135 | Araneidae | *Nephila pilipes* | 27.63 N 95.28 E | BOLD:AAW4519 | MK392712 | Hap_34 |
| 80 | AA-180 | ZSI_AA-180 | Araneidae | *Nephila pilipes* | 27.63 N 95.28 E | BOLD:AAW4519 | MK392713 | Hap_35 |
| 81 | AA-1067 | ZSI_AA-1067 | Araneidae | *Nephila pilipes* | 27.57 N 95.39 E | BOLD:AAW4519 | MK392714 | Hap_33 |
| 82 | AA-151 | ZSI_AA-151 | Araneidae | *Nephila pilipes* | 27.63 N 95.28 E | BOLD:AAW4519 | MK392715 | Hap_35 |
| 83 | AA-145 | ZSI_AA-145 | Araneidae | *Nephila pilipes* | 27.63 N 95.28 E | BOLD:AAW4519 | MK392716 | Hap_33 |
| 84 | AA-688 | ZSI_AA-688 | Araneidae | *Nephila pilipes* | 27.29 N 95.51 E | BOLD:AAW4519 | MK392717 | Hap_36 |
| 85 | AA-367 | ZSI_AA-367 | Araneidae | *Nephila pilipes* | 27.47 N 94.91 E | BOLD:AAW4519 | MK392718 | Hap_35 |
| 86 | AA-741 | ZSI_AA-741 | Araneidae | *Nephila pilipes* | 27.29 N 95.51 E | BOLD:AAW4519 | MK392719 | Hap_35 |
| 87 | AA-148 | ZSI_AA-148 | Araneidae | *Nephila pilipes* | 27.47 N 94.91 E | BOLD:AAW4519 | MK392720 | Hap_35 |
| 88 | AA-701 | ZSI_AA-701 | Araneidae | *Nephila pilipes* | 27.29 N 95.51 E | BOLD:AAW4519 | MK392721 | Hap_33 |
| 89 | AA-738 | ZSI_AA-738 | Araneidae | *Nephila pilipes* | 27.29 N 95.51 E | BOLD:AAW4519 | MK392722 | Hap_33 |
| 90 | AA-730 | ZSI_AA-730 | Araneidae | *Nephila pilipes* | 27.29 N 95.51 E | BOLD:AAW4519 | MK392723 | Hap_35 |
| 91 | AA-714 | ZSI_AA-714 | Araneidae | *Nephila pilipes* | 27.29 N 95.51 E | BOLD:AAW4519 | MK392724 | Hap_33 |
| 92 | AA-718 | ZSI_AA-718 | Araneidae | *Nephila pilipes* | 27.29 N 95.51 E | BOLD:AAW4519 | MK392725 | Hap_33 |
| 93 | AA-754 | ZSI_AA-754 | Araneidae | *Nephila pilipes* | 27.29 N 95.51 E | BOLD:AAW4519 | MK392726 | Hap_35 |
| 94 | AA-890 | ZSI_AA-890 | Araneidae | *Nephila pilipes* | 27.57 N 95.39 E | BOLD:AAW4519 | MK392727 | Hap_35 |
| 95 | AA-166 | ZSI_AA-166 | Araneidae | *Nephila pilipes* | 27.63 N 95.28 E | BOLD:AAW4519 | MK392728 | Hap_33 |
| 96 | AA-206 | ZSI_AA-206 | Araneidae | *Nephila pilipes* | 27.47 N 94.91 E | BOLD:AAW4519 | MK392729 | Hap_37 |
| 97 | AA-1192 | ZSI_AA-1192 | Araneidae | *Nephila pilipes* | 27.52 N 95.36 E | BOLD:AAW4519 | MK392730 | Hap_33 |
| 98 | AA-359 | ZSI_AA-359 | Araneidae | *Nephila pilipes* | 27.47 N 94.91 E | BOLD:AAW4519 | MK392731 | Hap_32 |
| 99 | AA-30 | ZSI_AA-30 | Araneidae | *Cyrtophora cicatrosa* | 23.41 N 87.11 E | BOLD:ACQ6199 | MK392732 | Hap_38 |
| 100 | AA-29 | ZSI_AA-29 | Araneidae | *Cyrtophora cicatrosa* | 23.41 N 87.11 E | BOLD:ACQ6199 | MK392733 | Hap_39 |
| 101 | AA-26 | ZSI_AA-26 | Araneidae | *Cyrtophora cicatrosa* | 23.41 N 87.11 E | BOLD:ACQ6199 | MK392734 | Hap_40 |
| 102 | AA-28 | ZSI_AA-28 | Araneidae | *Cyrtophora cicatrosa* | 23.41 N 87.11 E | BOLD:ACQ6199 | MK392735 | Hap_41 |
| 103 | AA-201 | ZSI_AA-201 | Araneidae | *Cyrtophora cicatrosa* | 22.81 N 88.53 E | BOLD:ACQ6199 | MK392736 | Hap_42 |
| 104 | AA-365 | ZSI_AA-365 | Araneidae | *Cyrtophora cicatrosa* | 27.47 N 94.91 E | BOLD:ACQ6199 | MK392737 | Hap_43 |
| 105 | AA-364 | ZSI_AA-364 | Araneidae | *Cyrtophora cicatrosa* | 27.47 N 94.91 E | BOLD:ACQ6199 | MK392738 | Hap_43 |
| 106 | AA-31 | ZSI_AA-31 | Araneidae | *Cyrtophora cicatrosa* | 23.41 N 87.11 E | BOLD:ACQ6199 | MK392739 | Hap_44 |
| 107 | AA-172 | ZSI_AA-172 | Araneidae | *Cyrtophora cicatrosa* | 24.86 N 92.56 E | BOLD:ACQ6199 | MK392740 | Hap_45 |
| 108 | AA-1042 | ZSI_AA-1042 | Araneidae | *Cyrtophora cicatrosa* | 24.86 N 92.56 E | BOLD:ACQ6199 | MK392741 | Hap_46 |
| 109 | AA-25 | ZSI_AA-25 | Araneidae | *Cyrtophora cicatrosa* | 22.81 N 88.53 E | BOLD:ACQ6199 | MK392742 | Hap_47 |
| 110 | AA-1051 | ZSI_AA-1051 | Araneidae | *Cyrtophora cicatrosa* | 24.86 N 92.56 E | BOLD:ACQ6199 | MK392743 | Hap_48 |
| 111 | AA-59 | ZSI_AA-59 | Araneidae | *Cyrtophora cicatrosa* | 23.03 N 88.70 E | BOLD:ACQ6199 | MK392744 | Hap_49 |
| 112 | AA-27 | ZSI_AA-27 | Araneidae | *Cyrtophora cicatrosa* | 23.41 N 87.11 E | BOLD:ACQ6199 | MK392745 | Hap_50 |
| 113 | AA-167 | ZSI_AA-167 | Araneidae | *Cyrtophora cicatrosa* | 24.86 N 92.56 E | BOLD:ACQ6199 | MK392746 | Hap_51 |
| 114 | AA-199 | ZSI_AA-199 | Araneidae | *Cyrtophora cicatrosa* | 22.39 N 86.94 E | BOLD:ACQ6199 | MK392747 | Hap_51 |
| 115 | AA-153 | ZSI_AA-153 | Araneidae | *Gasteracantha diadesmia* | 20.77 N 73.67 E | BOLD:ADA2059 | MK392748 | Hap_52 |
| 116 | AA-782 | ZSI_AA-782 | Araneidae | *Gasteracantha diadesmia* | 27.66 N 95.36 E | BOLD:ADA2059 | MK392749 | Hap_52 |
| 117 | AA-785 | ZSI_AA-785 | Araneidae | *Gasteracantha kuhli* | 27.66 N 95.36 E | BOLD:ACQ5948 | MK392750 | Hap_53 |
| 118 | AA-657 | ZSI_AA-657 | Araneidae | *Gasteracantha kuhli* | 27.52 N 95.36 E | BOLD:ACQ5948 | MK392751 | Hap_53 |
| 119 | AA-1006 | ZSI_AA-1006 | Araneidae | *Gasteracantha kuhli* | 27.57 N 95.39 E | BOLD:ACQ5948 | MK392752 | Hap_53 |
| 120 | AA-382 | ZSI_AA-382 | Araneidae | *Gasteracantha kuhli* | 26.67 N 92.85 E | BOLD:ACQ5948 | MK392753 | Hap_53 |
| 121 | AA-398 | ZSI_AA-398 | Araneidae | *Gasteracantha kuhli* | 26.67 N 92.85 E | BOLD:ACQ5948 | MK392754 | Hap_53 |
| 122 | AA-790 | ZSI_AA-790 | Araneidae | *Gasteracantha kuhli* | 27.66 N 95.36 E | BOLD:ACQ5948 | MK392755 | Hap_53 |
| 123 | AA-791 | ZSI_AA-791 | Araneidae | *Gasteracantha kuhli* | 27.66 N 95.36 E | BOLD:ACQ5948 | MK392756 | Hap_53 |
| 124 | AA-891 | ZSI_AA-891 | Araneidae | *Gasteracantha kuhli* | 27.57 N 95.39 E | BOLD:ACQ5948 | MK392757 | Hap_53 |
| 125 | AA-1005 | ZSI_AA-1005 | Araneidae | *Gasteracantha kuhli* | 27.57 N 95.39 E | BOLD:ACQ5948 | MK392758 | Hap_53 |
| 126 | AA-784 | ZSI_AA-784 | Araneidae | *Gasteracantha kuhli* | 27.66 N 95.36 E | BOLD:ACQ5948 | MK392759 | Hap_53 |
| 127 | AA-1130 | ZSI_AA-1130 | Araneidae | *Gasteracantha kuhli* | 26.40 N 74.81 E | BOLD:ACQ5948 | MK392760 | Hap_54 |
| 128 | AA-154 | ZSI_AA-154 | Araneidae | *Gasteracantha kuhli* | 20.77 N 73.67 E | BOLD:ACQ5948 | MK392761 | Hap_53 |
| 129 | AA-786 | ZSI_AA-786 | Araneidae | *Gasteracantha kuhli* | 27.66 N 95.36 E | BOLD:ACQ5948 | MK392762 | Hap_53 |
| 130 | AA-892 | ZSI_AA-892 | Araneidae | *Gasteracantha kuhli* | 27.57 N 95.39 E | BOLD:ACQ5948 | MK392763 | Hap_53 |
| 131 | AA-894 | ZSI_AA-894 | Araneidae | *Gasteracantha kuhli* | 27.57 N 95.39 E | BOLD:ACQ5948 | MK392764 | Hap_53 |
| 132 | AA-155 | ZSI_AA-155 | Araneidae | *Gasteracantha kuhli* | 26.40 N 74.81 E | BOLD:ACQ5948 | MK392765 | Hap_53 |
| 133 | AA-878 | ZSI_AA-878 | Araneidae | *Gasteracantha kuhli* | 27.57 N 95.33 E | BOLD:ACQ5948 | MK392766 | Hap_53 |
| 134 | AA-735 | ZSI_AA-735 | Araneidae | *Gasteracantha hasselti* | 27.29 N 95.51 E | BOLD:ADB4780 | MK392767 | Hap_55 |
| 135 | AA-736 | ZSI_AA-736 | Araneidae | *Gasteracantha hasselti* | 27.29 N 95.51 E | BOLD:ADB4780 | MK392768 | Hap_55 |
| 136 | AA-708 | ZSI_AA-708 | Araneidae | *Gasteracantha hasselti* | 27.29 N 95.51 E | BOLD:ADB4780 | MK392769 | Hap_55 |
| 137 | AA-695 | ZSI_AA-695 | Araneidae | *Gasteracantha hasselti* | 27.29 N 95.51 E | BOLD:ADB4780 | MK392770 | Hap_55 |
| 138 | AA-704 | ZSI_AA-704 | Araneidae | *Gasteracantha hasselti* | 27.29 N 95.51 E | BOLD:ADB4780 | MK392771 | Hap_55 |
| 139 | AA-751 | ZSI_AA-751 | Araneidae | *Gasteracantha hasselti* | 27.29 N 95.51 E | BOLD:ADB4780 | MK392772 | Hap_55 |
| 140 | AA-1127 | ZSI_AA-1127 | Araneidae | *Gasteracantha hasselti* | 20.77 N 73.67 E | BOLD:ADB4780 | MK392773 | Hap_55 |
| 141 | AA-1158 | ZSI_AA-1158 | Araneidae | *Gasteracantha hasselti* | 21.00 N 72.85 E | BOLD:ADB4780 | MK392774 | Hap_56 |
| 142 | AA-527 | ZSI_AA-527 | Araneidae | *Eriovixia poonaensis* | 26.20 N 92.93 E | BOLD:ADG4485 | MK392775 | Hap_57 |
| 143 | AA-965 | ZSI_AA-965 | Araneidae | *Eriovixia poonaensis* | 27.52 N 95.36 E | BOLD:ADG4485 | MK392776 | Hap_58 |
| 144 | AA-703 | ZSI_AA-703 | Araneidae | *Eriovixia poonaensis* | 27.29 N 95.51 E | BOLD:ADG4485 | MK392777 | Hap_59 |
| 145 | AA-711 | ZSI_AA-711 | Theridiidae | *Thwaitesia* sp. | 27.29 N 95.51 E | BOLD:ADI1723 | MK392778 | Hap_60 |
| 146 | AA-816 | ZSI_AA-816 | Theridiidae | *Thwaitesia* sp. | 27.66 N 95.36 E | BOLD:ADI1723 | MK392779 | Hap_61 |
| 147 | AA-177 | ZSI_AA-177 | Theridiidae | *Nesticodes rufipes* | 20.76 N 73.37 E | BOLD:AAG4814 | MK392780 | Hap_62 |
| 148 | AA-558 | ZSI_AA-558 | Theridiidae | *Nesticodes rufipes* | 27.02 N 74.21 E | BOLD:AAG4814 | MK392781 | Hap_62 |
| 149 | AA-03 | ZSI_AA-03 | Theridiidae | *Nesticodes rufipes* | 22.81 N 88.53 E | BOLD:AAG4814 | MK392782 | Hap_62 |
| 150 | AA-66 | ZSI_AA-66 | Theridiidae | *Nesticodes rufipes* | 22.85 N 88.53 E | BOLD:AAG4814 | MK392783 | Hap_62 |
| 151 | AA-780 | ZSI_AA-780 | Theridiidae | *Nihonhimea mundula* | 27.52 N 95.36 E | BOLD:ADI3786 | MK392784 | Hap_63 |
| 152 | AA-885 | ZSI_AA-885 | Theridiidae | *Nihonhimea mundula* | 27.57 N 95.33 E | BOLD:ADI3786 | MK392785 | Hap_64 |
| 153 | AA-871 | ZSI_AA-871 | Araneidae | *Cyclosa quinqueguttata* | 27.57 N 95.33 E | - | MK392786 | Hap_65 |
| 154 | AA-841 | ZSI_AA-841 | Araneidae | *Chorizopes quadrituberculata* | 27.66 N 95.36 E | BOLD:ADI1914 | MK392787 | Hap_66 |
| 155 | AA-1234 | ZSI_AA-1234 | Deinopidae | *Deinopis* sp. | 23.22 N 72.67 E | BOLD:ADM1540 | MK392788 | Hap_67 |
| 156 | AA-485 | ZSI_AA-485 | Eutichuridae | *Cheiracanthium triviale* | 26.20 N 92.93 E | BOLD:ADG5083 | MK392789 | Hap_68 |
| 157 | AA-1239 | ZSI_AA-1239 | Eutichuridae | *Cheiracanthium triviale* | 23.22 N 72.67 E | BOLD:ADL9949 | MK392790 | Hap_69 |
| 158 | AA-1245 | ZSI_AA-1245 | Eutichuridae | *Cheiracanthium triviale* | 23.22 N 72.67 E | BOLD:ADL9949 | MK392791 | Hap_70 |
| 159 | AA-1235 | ZSI_AA-1235 | Eutichuridae | *Cheiracanthium triviale* | 23.22 N 72.67 E | BOLD:ADL9949 | MK392792 | Hap_70 |
| ­­­­­-160 | AA-1238 | ZSI_AA-1238 | Eutichuridae | *Cheiracanthium triviale* | 23.22 N 72.67 E | BOLD:ADL9949 | MK392793 | Hap_69 |
| 161 | AA-1050 | ZSI_AA-1050 | Eutichuridae | *Cheiracanthium triviale* | 26.20 N 92.93 E | BOLD:ADG5083 | MK392794 | Hap_68 |
| 162 | AA-391 | ZSI_AA-391 | Salticidae | *Myrmarachne kiboschensis* | 26.67 N 92.85 E | BOLD:ADF4057 | MK392795 | Hap_71 |
| 163 | AA-530 | ZSI_AA-530 | Salticidae | *Plexippus petersi* | 22.80 N 88.64 E | BOLD:AAH6923 | MK392796 | Hap_72 |
| 164 | AA-453 | ZSI_AA-453 | Salticidae | *Plexippus petersi* | 22.81 N 83.53 E | BOLD:AAH6923 | MK392797 | Hap_72 |
| 165 | AA-52 | ZSI_AA-52 | Salticidae | *Plexippus petersi* | 23.10 N 86.96 E | BOLD:AAH6923 | MK392798 | Hap_72 |
| 166 | AA-55 | ZSI_AA-55 | Salticidae | *Plexippus petersi* | 23.33 N 86.02 E | BOLD:AAH6923 | MK392799 | Hap_72 |
| 167 | AA-458 | ZSI_AA-458 | Salticidae | *Plexippus petersi* | 22.81 N 83.53 E | BOLD:AAH6923 | MK392800 | Hap_72 |
| 168 | AA-409 | ZSI_AA-409 | Salticidae | *Plexippus petersi* | 22.81 N 88.53 E | BOLD:AAH6923 | MK392801 | Hap_72 |
| 169 | AA-408 | ZSI_AA-408 | Salticidae | *Plexippus petersi* | 22.81 N 88.53 E | BOLD:AAH6923 | MK392802 | Hap_72 |
| 170 | AA-50 | ZSI_AA-50 | Salticidae | *Plexippus petersi* | 22.81 N 88.53 E | - | MK392803 | Hap_72 |
| 171 | AA-457 | ZSI_AA-457 | Salticidae | *Plexippus petersi* | 22.81 N 83.53 E | BOLD:AAH6923 | MK392804 | Hap_72 |
| 172 | AA-412 | ZSI_AA-412 | Salticidae | *Plexippus petersi* | 22.81 N 88.53 E | BOLD:AAH6923 | MK392805 | Hap_72 |
| 173 | AA-57 | ZSI_AA-57 | Salticidae | *Plexippus petersi* | 27.73 N 95.79 E | BOLD:AAH6923 | MK392806 | Hap_72 |
| 174 | AA-411 | ZSI_AA-411 | Salticidae | *Plexippus petersi* | 22.81 N 88.53 E | BOLD:AAH6923 | MK392807 | Hap_72 |
| 175 | AA-452 | ZSI_AA-452 | Salticidae | *Plexippus petersi* | 22.81 N 83.53 E | BOLD:AAH6923 | MK392808 | Hap_72 |
| 176 | AA-404 | ZSI_AA-404 | Salticidae | *Plexippus petersi* | 27.44 N 94.61 E | BOLD:AAH6923 | MK392809 | Hap_72 |
| 177 | AA-524 | ZSI_AA-524 | Salticidae | *Plexippus paykulli* | 26.20 N 92.93 E | BOLD:ACU8433 | MK392810 | Hap_73 |
| 178 | AA-1066 | ZSI_AA-1066 | Salticidae | *Plexippus paykulli* | 27.57 N 95.39 E | BOLD:ACU8433 | MK392811 | Hap_74 |
| 179 | AA-528 | ZSI_AA-528 | Salticidae | *Plexippus paykulli* | 22.80 N 88.64 E | BOLD:AAO2152 | MK392812 | Hap_75 |
| 180 | AA-339 | ZSI_AA-339 | Salticidae | *Telamonia dimidiata* | 22.68 N 88.65 E | BOLD:ADG6800 | MK392813 | Hap_76 |
| 181 | AA-779 | ZSI_AA-779 | Salticidae | *Yaginumaella incognita* | 27.52 N 95.36 E | BOLD:ADI2821 | MK392814 | Hap_77 |
| 182 | AA-772 | ZSI_AA-772 | Salticidae | *Yaginumaella incognita* | 27.52 N 95.36 E | BOLD:ADI2821 | MK392815 | Hap_77 |
| 183 | AA-843 | ZSI_AA-843 | Salticidae | *Yaginumaella incognita* | 27.66 N 95.36 E | BOLD:ADI2821 | MK392816 | Hap_78 |
| 184 | AA-820 | ZSI_AA-820 | Salticidae | *Yaginumaella incognita* | 27.66 N 95.36 E | BOLD:ADI2821 | MK392817 | Hap_79 |
| 185 | AA-656 | ZSI_AA-656 | Salticidae | *Yaginumaella incognita* | 27.52 N 95.36 E | BOLD:ADI2821 | MK392818 | Hap_78 |
| 186 | AA-861 | ZSI_AA-861 | Salticidae | *Yaginumaella incognita* | 27.66 N 95.36 E | BOLD:ADI2821 | MK392819 | Hap_77 |
| 187 | AA-499 | ZSI_AA-499 | Salticidae | *Phintella accentifera* | 26.20 N 92.93 E | BOLD:ADG7647 | MK392820 | Hap_80 |
| 188 | AA-667 | ZSI_AA-667 | Salticidae | *Phintella accentifera* | 27.52 N 95.36 E | BOLD:ADG7647 | MK392821 | Hap_81 |
| 189 | AA-1055 | ZSI_AA-1055 | Salticidae | *Phintella vittata* | 27.52 N 95.36 E | BOLD:ADI5207 | MK392822 | Hap_82 |
| 190 | AA-986 | ZSI_AA-986 | Salticidae | *Phintella vittata* | 23.01 N 72.44 E | BOLD:ADI5208 | MK392823 | Hap_83 |
| 191 | AA-1246 | ZSI_AA-1246 | Salticidae | *Phintella vittata* | 23.22 N 72.67 E | BOLD:ADI5208 | MK392824 | Hap_84 |
| 192 | AA-990 | ZSI_AA-990 | Salticidae | *Phintella vittata* | 27.74 N 95.52 E | BOLD:ADI5207 | MK392825 | Hap_85 |
| 193 | AA-378 | ZSI_AA-378 | Salticidae | *Thiania bhamoensis* | 26.68 N 92.81 E | BOLD:ADF4226 | MK392826 | Hap_86 |
| 194 | AA-384 | ZSI_AA-384 | Salticidae | *Thiania bhamoensis* | 26.68 N 92.81 E | BOLD:ADF4226 | MK392827 | Hap_86 |
| 195 | AA-670 | ZSI_AA-670 | Salticidae | *Thiania bhamoensis* | 26.68 N 92.81 E | BOLD:ADF4226 | MK392828 | Hap_86 |
| 196 | AA-928 | ZSI_AA-928 | Salticidae | *Thiania bhamoensis* | 26.68 N 92.81 E | BOLD:ADF4226 | MK392829 | Hap_86 |
| 197 | AA-669 | ZSI_AA-669 | Salticidae | *Thiania bhamoensis* | 27.59 N 95.68 E | BOLD:ADI5004 | MK392830 | Hap_87 |
| 198 | AA-955 | ZSI_AA-955 | Salticidae | *Thiania bhamoensis* | 27.59 N 95.68 E | BOLD:ADI5004 | MK392831 | Hap_88 |
| 199 | AA-925 | ZSI_AA-925 | Salticidae | *Thiania bhamoensis* | 27.59 N 95.68 E | BOLD:ADI5004 | MK392832 | Hap_89 |
| 200 | AA-672 | ZSI_AA-672 | Salticidae | *Thiania bhamoensis* | 26.70 N 93.14 E | BOLD:ADI1916 | MK392833 | Hap_90 |
| 201 | AA-1040 | ZSI_AA-1040 | Salticidae | *Thiania bhamoensis* | 26.70 N 93.14 E | BOLD:ADI1916 | MK392834 | Hap_91 |
| 202 | AA-559 | ZSI_AA-559 | Salticidae | *Epocilla sirohi* | 24.57 N 72.16 E | BOLD:ADH2742 | MK392835 | Hap_92 |
| 203 | AA-858 | ZSI_AA-858 | Salticidae | *Siler semiglaucus* | 27.66 N 95.36 E | BOLD:ADI4920 | MK392836 | Hap_93 |
| 204 | AA-998 | ZSI_AA-998 | Salticidae | *Siler semiglaucus* | 27.52 N 95.36 E | BOLD:ADI4920 | MK392837 | Hap_94 |
| 205 | AA-363 | ZSI_AA-363 | Salticidae | *Phintella versicolor* | 27.47 N 94.91 E | BOLD:ADF2927 | MK392838 | Hap_95 |
| 206 | AA-537 | ZSI_AA-537 | Salticidae | *Menemerus bivittatus* | 22.81 N 88.53 E | BOLD:AAL7843 | MK392839 | Hap_96 |
| 207 | AA-1044 | ZSI_AA-1044 | Salticidae | *Menemerus bivittatus* | 24.86 N 92.56 E | BOLD:AAL7843 | MK392840 | Hap_97 |
| 208 | AA-451 | ZSI_AA-451 | Salticidae | *Menemerus nigli* | 22.81 N 83.53 E | BOLD:AAQ0156 | MK392841 | Hap_98 |
| 209 | AA-560 | ZSI_AA-560 | Salticidae | *Mogrus rajasthanensis* | 24.59 N 72.72 E | BOLD:ACZ1977 | MK392842 | Hap_99 |
| 210 | AA-354 | ZSI_AA-354 | Salticidae | *Hasarius adansoni* | 22.81 N 88.53 E | BOLD:AAW0165 | MK392843 | Hap_100 |
| 211 | AA-461 | ZSI_AA-461 | Salticidae | *Hasarius adansoni* | 22.81 N 83.53 E | BOLD:AAW0165 | MK392844 | Hap_100 |
| 212 | AA-407 | ZSI_AA-407 | Salticidae | *Hasarius adansoni* | 22.81 N 88.53 E | BOLD:AAW0165 | MK392845 | Hap_100 |
| 213 | AA-752 | ZSI_AA-752 | Salticidae | *Pancorius magnus* | 27.29 N 95.51 E | BOLD:ADI3938 | MK392846 | Hap_101 |
| 214 | AA-814 | ZSI_AA-814 | Salticidae | *Pancorius magnus* | 27.66 N 95.36 E | BOLD:ADI3938 | MK392847 | Hap_101 |
| 215 | AA-1058 | ZSI_AA-1058 | Salticidae | *Pancorius magnus* | 27.52 N 95.36 E | BOLD:ADI3938 | MK392848 | Hap_101 |
| 216 | AA-1060 | ZSI_AA-1060 | Salticidae | *Pancorius magnus* | 27.52 N 95.36 E | BOLD:ADI3938 | MK392849 | Hap_101 |
| 217 | AA-383 | ZSI_AA-383 | Salticidae | *Rhene flavicomans* | 26.67 N 92.85 E | BOLD:ADF4276 | MK392850 | Hap_102 |
| 218 | AA-500 | ZSI_AA-500 | Uloboridae | *Uloborus* sp. | 26.20 N 92.93 E | BOLD:ADG6648 | MK392851 | Hap_103 |
| 219 | AA-721 | ZSI_AA-721 | Linyphiidae | *Linyphia sikkimensis* | 27.29 N 95.51 E | BOLD:ADI2332 | MK392852 | Hap_104 |
| 220 | AA-700 | ZSI_AA-700 | Linyphiidae | *Linyphia sikkimensis* | 27.29 N 95.51 E | BOLD:ADI2332 | MK392853 | Hap_105 |
| 221 | AA-732 | ZSI_AA-732 | Linyphiidae | *Linyphia sikkimensis* | 27.29 N 95.51 E | BOLD:ADI2332 | MK392854 | Hap_105 |
| 222 | AA-722 | ZSI_AA-722 | Linyphiidae | *Linyphia sikkimensis* | 27.29 N 95.51 E | BOLD:ADI2332 | MK392855 | Hap_105 |
| 223 | AA-706 | ZSI_AA-706 | Linyphiidae | *Linyphia sikkimensis* | 27.29 N 95.51 E | BOLD:ADI2332 | MK392856 | Hap_106 |
| 224 | AA-544 | ZSI_AA-544 | Tetragnathidae | *Leucauge celebesiana* | 27.20 N 88.70 E | BOLD:ACH2718 | MK392857 | Hap_107 |
| 225 | AA-1788 | ZSI_AA-721 | Tetragnathidae | *Leucauge celebesiana* | 23.22 N 72.67 E | BOLD:ACH2718 | MK392858 | Hap_107 |
| 226 | AA-1801 | ZSI_AA-739 | Tetragnathidae | *Leucauge celebesiana* | 23.22 N 72.67 E | BOLD:ACH2718 | MK392859 | Hap_107 |
| 227 | AA-1806 | ZSI_AA-776 | Tetragnathidae | *Leucauge celebesiana* | 23.22 N 72.67 E | BOLD:ACH2718 | MK392860 | Hap_107 |
| 228 | AA-1785 | ZSI_AA-716 | Tetragnathidae | *Leucauge celebesiana* | 23.22 N 72.67 E | BOLD:ACH2718 | MK392861 | Hap_107 |
| 229 | AA-1800 | ZSI_AA-735 | Tetragnathidae | *Leucauge celebesiana* | 27.29 N 95.51 E | BOLD:ACH2718 | MK392862 | Hap_107 |
| 230 | AA-1793 | ZSI_AA-726 | Tetragnathidae | *Leucauge celebesiana* | 27.29 N 95.51 E | BOLD:ACH2718 | MK392863 | Hap_107 |
| 231 | AA-1802 | ZSI_AA-740 | Tetragnathidae | *Leucauge celebesiana* | 27.29 N 95.51 E | BOLD:ACH2718 | MK392864 | Hap_107 |
| 232 | AA-716 | ZSI_AA-716 | Tetragnathidae | *Leucauge celebesiana* | 27.29 N 95.51 E | BOLD:ACH2718 | MK392865 | Hap_107 |
| 233 | AA-1783 | ZSI_AA-713 | Tetragnathidae | *Leucauge celebesiana* | 23.22 N 72.67 E | BOLD:ACH2718 | MK392866 | Hap_107 |
| 234 | AA-1790 | ZSI_AA-723 | Tetragnathidae | *Leucauge celebesiana* | 23.22 N 72.67 E | BOLD:ACH2718 | MK392867 | Hap_107 |
| 235 | AA-1804 | ZSI_AA-771 | Tetragnathidae | *Leucauge celebesiana* | 23.22 N 72.67 E | BOLD:ADI4857 | MK392868 | Hap_107 |
| 236 | AA-1789 | ZSI_AA-722 | Tetragnathidae | *Leucauge celebesiana* | 23.22 N 72.67 E | - | MK392869 | Hap_107 |
| 237 | AA-1803 | ZSI_AA-741 | Tetragnathidae | *Leucauge celebesiana* | 23.22 N 72.67 E | BOLD:AAW4519 | MK392870 | Hap_107 |
| 238 | AA-162 | ZSI_AA-162 | Tetragnathidae | *Leucauge tessellata* | 27.29 N 95.51 E | BOLD:AAX0513 | MK392871 | Hap_108 |
| 239 | AA-638 | ZSI_AA-638 | Tetragnathidae | *Leucauge tessellata* | 27.52 N 95.36 E | BOLD:AAX0513 | MK392872 | Hap_109 |
| 240 | AA-526 | ZSI_AA-526 | Tetragnathidae | *Leucauge tessellata* | 26.20 N 92.93 E | BOLD:AAX0513 | MK392873 | Hap_109 |
| 241 | AA-511 | ZSI_AA-511 | Tetragnathidae | *Leucauge tessellata* | 26.20 N 92.93 E | BOLD:AAX0513 | MK392874 | Hsp_110 |
| 242 | AA-1019 | ZSI_AA-1019 | Tetragnathidae | *Leucauge tessellata* | 27.57 N 95.39 E | BOLD:AAX0513 | MK392875 | Hap_109 |
| 243 | AA-1029 | ZSI_AA-1029 | Tetragnathidae | *Leucauge tessellata* | 27.57 N 95.39 E | BOLD:AAX0513 | MK392876 | Hap_109 |
| 244 | AA-1008 | ZSI_AA-1008 | Tetragnathidae | *Leucauge tessellata* | 27.57 N 95.39 E | BOLD:AAX0513 | MK392877 | Hap_109 |
| 245 | AA-889 | ZSI_AA-889 | Tetragnathidae | *Leucauge tessellata* | 27.57 N 95.39 E | BOLD:AAX0513 | MK392878 | Hap_111 |
| 246 | AA-1030 | ZSI_AA-1030 | Tetragnathidae | *Leucauge tessellata* | 27.57 N 95.39 E | BOLD:AAX0513 | MK392879 | Hap_112 |
| 247 | AA-895 | ZSI_AA-895 | Tetragnathidae | *Leucauge tessellata* | 27.57 N 95.39 E | BOLD:AAX0513 | MK392880 | Hap_109 |
| 248 | AA-169 | ZSI_AA-169 | Tetragnathidae | *Opadometa fastigata* | 26.79 N 92.69 E | BOLD:ABY1733 | MK392881 | Hap_113 |
| 249 | AA-188 | ZSI_AA-188 | Tetragnathidae | *Opadometa fastigata* | 26.79 N 92.69 E | BOLD:ABY1733 | MK392882 | Hap_113 |
| 250 | AA-371 | ZSI_AA-371 | Tetragnathidae | *Opadometa fastigata* | 26.81 N 93.64 E | BOLD:ABY1733 | MK392883 | Hap_113 |
| 251 | AA-757 | ZSI_AA-757 | Tetragnathidae | *Opadometa fastigata* | 26.81 N 93.31 E | BOLD:ABY1733 | MK392884 | Hap_113 |
| 252 | AA-1002 | ZSI_AA-1002 | Tetragnathidae | *Opadometa fastigata* | 26.68 N 92.91 E | BOLD:ABY1733 | MK392885 | Hap_113 |
| 253 | AA-799 | ZSI_AA-799 | Tetragnathidae | *Opadometa fastigata* | 26.81 N 93.64 E | BOLD:ABY1733 | MK392886 | Hap_113 |
| 254 | AA-758 | ZSI_AA-758 | Tetragnathidae | *Opadometa fastigata* | 26.81 N 93.31 E | BOLD:ABY1733 | MK392887 | Hap_113 |
| 255 | AA-756 | ZSI_AA-756 | Tetragnathidae | *Opadometa fastigata* | 26.79 N 92.69 E | BOLD:ABY1733 | MK392888 | Hap_113 |
| 256 | AA-897 | ZSI_AA-897 | Tetragnathidae | *Opadometa fastigata* | 26.81 N 93.31 E | BOLD:ABY1733 | MK392889 | Hap_113 |
| 257 | AA-802 | ZSI_AA-802 | Tetragnathidae | *Opadometa fastigata* | 26.79 N 92.69 E | BOLD:ABY1733 | MK392890 | Hap_113 |
| 258 | AA-1368 | ZSI_AA-1368 | Tetragnathidae | *Leucauge xiaoen* | 13.01 N 77.56 E | BOLD:AAG8516 | MK392891 | Hap_114 |
| 259 | AA-1367 | ZSI_AA-1367 | Tetragnathidae | *Leucauge xiaoen* | 13.01 N 77.56 E | BOLD:AAG8516 | MK392892 | Hap_115 |
| 260 | AA-1370 | ZSI_AA-1370 | Tetragnathidae | *Leucauge xiaoen* | 13.01 N 77.56 E | BOLD:AAG8516 | MK392893 | Hap_114 |
| 261 | AA-1436 | ZSI_AA-1436 | Tetragnathidae | *Leucauge xiaoen* | 13.01 N 77.56 E | BOLD:AAG8516 | MK392894 | Hap_116 |
| 262 | AA-1369 | ZSI_AA-1369 | Tetragnathidae | *Leucauge xiaoen* | 13.01 N 77.56 E | BOLD:AAG8516 | MK392895 | Hap_117 |
| 263 | AA-168 | ZSI_AA-168 | Tetragnathidae | *Leucauge xiaoen* | 26.79 N 92.69 E | BOLD:AAG8516 | MK392896 | Hap_118 |
| 264 | AA-372 | ZSI_AA-372 | Tetragnathidae | *Leucauge xiaoen* | 26.67 N 92.85 E | BOLD:AAG8516 | MK392897 | Hap_118 |
| 265 | AA-392 | ZSI_AA-392 | Tetragnathidae | *Leucauge xiaoen* | 26.67 N 92.85 E | BOLD:AAG8516 | MK392898 | Hap_119 |
| 266 | AA-390 | ZSI_AA-390 | Tetragnathidae | *Leucauge xiaoen* | 26.67 N 92.85 E | BOLD:AAG8516 | MK392899 | Hap_120 |
| 267 | AA-635 | ZSI_AA-635 | Tetragnathidae | *Leucauge xiaoen* | 27.52 N 95.36 E | BOLD:AAG8516 | MK392900 | Hap_121 |
| 268 | AA-633 | ZSI_AA-633 | Tetragnathidae | *Leucauge xiaoen* | 27.52 N 95.36 E | BOLD:AAG8516 | MK392901 | Hap_118 |
| 269 | AA-640 | ZSI_AA-640 | Tetragnathidae | *Leucauge xiaoen* | 27.52 N 95.36 E | BOLD:AAG8516 | MK392902 | Hap_118 |
| 270 | AA-881 | ZSI_AA-881 | Tetragnathidae | *Leucauge xiaoen* | 27.57 N 95.33 E | BOLD:AAG8516 | MK392903 | Hap_115 |
| 271 | AA-872 | ZSI_AA-872 | Tetragnathidae | *Leucauge xiaoen* | 27.57 N 95.33 E | BOLD:AAG8516 | MK392904 | Hap_118 |
| 272 | AA-637 | ZSI_AA-637 | Tetragnathidae | *Leucauge xiaoen* | 27.52 N 95.36 E | BOLD:AAG8516 | MK392905 | Hap_118 |
| 273 | AA-639 | ZSI_AA-639 | Tetragnathidae | *Leucauge xiaoen* | 27.52 N 95.36 E | BOLD:AAG8516 | MK392906 | Hap_118 |
| 274 | AA-641 | ZSI_AA-641 | Tetragnathidae | *Leucauge xiaoen* | 27.52 N 95.36 E | BOLD:AAG8516 | MK392907 | Hap_118 |
| 275 | AA-636 | ZSI_AA-636 | Tetragnathidae | *Leucauge xiaoen* | 27.52 N 95.36 E | BOLD:AAG8516 | MK392908 | Hap_118 |
| 276 | AA-659 | ZSI_AA-659 | Tetragnathidae | *Leucauge xiaoen* | 27.52 N 95.36 E | BOLD:AAG8516 | MK392909 | Hap_118 |
| 277 | AA-683 | ZSI_AA-683 | Tetragnathidae | *Leucauge xiaoen* | 27.52 N 95.36 E | BOLD:AAG8516 | MK392910 | Hap_118 |
| 278 | AA-681 | ZSI_AA-681 | Tetragnathidae | *Leucauge xiaoen* | 27.52 N 95.36 E | BOLD:AAG8516 | MK392911 | Hap_115 |
| 279 | AA-1011 | ZSI_AA-1011 | Tetragnathidae | *Leucauge xiaoen* | 27.57 N 95.39 E | BOLD:AAG8516 | MK392912 | Hap_122 |
| 280 | AA-874 | ZSI_AA-874 | Tetragnathidae | *Leucauge xiaoen* | 27.57 N 95.33 E | BOLD:AAG8516 | MK392913 | Hap_118 |
| 281 | AA-653 | ZSI_AA-653 | Tetragnathidae | *Leucauge xiaoen* | 27.52 N 95.36 E | BOLD:AAG8516 | MK392914 | Hap_115 |
| 282 | AA-682 | ZSI_AA-682 | Tetragnathidae | *Leucauge xiaoen* | 27.52 N 95.36 E | BOLD:AAG8516 | MK392915 | Hap_115 |
| 283 | AA-804 | ZSI_AA-804 | Tetragnathidae | *Leucauge xiaoen* | 27.66 N 95.36 E | BOLD:AAG8516 | MK392916 | Hap_115 |
| 284 | AA-800 | ZSI_AA-800 | Tetragnathidae | *Leucauge xiaoen* | 27.66 N 95.36 E | BOLD:AAG8516 | MK392917 | Hap_123 |
| 285 | AA-202 | ZSI_AA-202 | Tetragnathidae | *Leucauge xiaoen* | 27.57 N 95.39 E | BOLD:AAG8516 | MK392918 | Hap_118 |
| 286 | AA-203 | ZSI_AA-203 | Tetragnathidae | *Leucauge xiaoen* | 27.57 N 95.39 E | BOLD:AAG8516 | MK392919 | Hap_118 |
| 287 | AA-400 | ZSI_AA-400 | Tetragnathidae | *Leucauge xiaoen* | 26.67 N 92.85 E | BOLD:AAG8516 | MK392920 | Hap_118 |
| 288 | AA-164 | ZSI_AA-164 | Theridiidae | *Argyrodes flavescens* | 27.47 N 94.91 E | BOLD:ADA1229 | MK392921 | Hap_124 |
| 289 | AA-185 | ZSI_AA-185 | Theridiidae | *Argyrodes flavescens* | 27.29 N 95.51 E | BOLD:ADA1229 | MK392922 | Hap_125 |
| 290 | AA-997 | ZSI_AA-997 | Theridiidae | *Argyrodes flavescens* | 27.52 N 95.36 E | BOLD:ADA1229 | MK392923 | Hap_124 |
| 291 | AA-1001 | ZSI_AA-1001 | Theridiidae | *Argyrodes flavescens* | 27.52 N 95.36 E | BOLD:ADA1229 | MK392924 | Hap_125 |
| 292 | AA-698 | ZSI_AA-698 | Theridiidae | *Argyrodes flavescens* | 27.29 N 95.51 E | BOLD:ADA1229 | MK392925 | Hap_125 |
| 293 | AA-272 | ZSI_AA-272 | Theridiidae | *Ariamnes simulans* | 22.45 N 88.30 E | BOLD:ADF4020 | MK392926 | Hap_126 |
| 294 | AA-961 | ZSI_AA-961 | Theridiidae | *Argyrodes projeles* | 27.52 N 95.36 E | BOLD:ADI2090 | MK392927 | Hap_127 |
| 295 | AA-289 | ZSI_AA-289 | Sparassidae | *Heteropoda venatoria* | 21.73 N 88.83 E | BOLD:AAL8190 | MK392928 | Hap_128 |
| 296 | AA-303 | ZSI_AA-303 | Sparassidae | *Heteropoda venatoria* | 21.73 N 88.83 E | BOLD:AAL8190 | MK392929 | Hap_128 |
| 297 | AA-521 | ZSI_AA-521 | Sparassidae | *Heteropoda venatoria* | 27.52 N 95.36 E | BOLD:ACN9567 | MK392930 | Hap_129 |
| 298 | AA-01 | ZSI_AA-01 | Sparassidae | *Heteropoda venatoria* | 21.73 N 88.83 E | BOLD:ACN9567 | MK392931 | Hap_130 |
| 299 | AA-522 | ZSI_AA-522 | Sparassidae | *Heteropoda venatoria* | 26.20 N 92.93 E | BOLD:ACN9567 | MK392932 | Hap_131 |
| 300 | AA-478 | ZSI_AA-478 | Sparassidae | *Heteropoda venatoria* | 26.20 N 92.93 E | BOLD:ACN9567 | MK392933 | Hap_132 |
| 301 | AA-1195 | ZSI_AA-1195 | Sparassidae | *Heteropoda venatoria* | 26.20 N 92.93 E | BOLD:ACN9567 | MK392934 | Hap_131 |
| 302 | AA-496 | ZSI_AA-496 | Sparassidae | *Heteropoda venatoria* | 27.52 N 95.36 E | BOLD:ACN9567 | MK392935 | Hap_129 |
| 303 | AA-311 | ZSI_AA-311 | Sparassidae | *Heteropoda venatoria* | 27.52 N 95.36 E | BOLD:ACN9567 | MK392936 | Hap_129 |
| 304 | AA-460 | ZSI_AA-460 | Sparassidae | *Heteropoda venatoria* | 26.20 N 92.93 E | BOLD:ACN9567 | MK392937 | Hap_133 |
| 305 | AA-1064 | ZSI_AA-1064 | Sparassidae | *Heteropoda venatoria* | 27.52 N 95.36 E | BOLD:ACN9567 | MK392938 | Hap_132 |
| 306 | AA-590 | ZSI_AA-590 | Sparassidae | *Sinopoda* sp. | 28.60 N 95.49 E | BOLD:ADG9620 | MK392939 | Hap_134 |
| 307 | AA-607 | ZSI_AA-607 | Sparassidae | *Sinopoda* sp. | 28.60 N 95.49 E | BOLD:ADG9620 | MK392940 | Hap_135 |
| 308 | AA-609 | ZSI_AA-609 | Sparassidae | *Pseudopoda cheppe* | 28.60 N 95.49 E | BOLD:ADI4536 | MK392941 | Hap_136 |
| 309 | AA-191 | ZSI_AA-191 | Tetragnathidae | *Orsinome vethi* | 27.66 N 95.86 E | BOLD:AAW8969 | MK392942 | Hap_137 |
| 310 | AA-204 | ZSI_AA-204 | Tetragnathidae | *Orsinome vethi* | 27.66 N 95.86 E | BOLD:AAW8969 | MK392943 | Hap_137 |
| 311 | AA-494 | ZSI_AA-494 | Tetragnathidae | *Tylorida striata* | 26.20 N 92.93 E | BOLD:ADG8135 | MK392944 | Hap_138 |
| 312 | AA-375 | ZSI_AA-375 | Tetragnathidae | *Tylorida ventralis* | 26.67 N 92.85 E | BOLD:ADF3786 | MK392945 | Hap_139 |
| 313 | AA-393 | ZSI_AA-393 | Tetragnathidae | *Tylorida ventralis* | 26.67 N 92.85 E | BOLD:ADF3786 | MK392946 | Hap_140 |
| 314 | AA-1012 | ZSI_AA-1012 | Tetragnathidae | *Tylorida ventralis* | 27.57 N 95.39 E | BOLD:ADF3786 | MK392947 | Hap_141 |
| 315 | AA-1025 | ZSI_AA-1025 | Tetragnathidae | *Tylorida ventralis* | 27.57 N 95.39 E | BOLD:ADF3786 | MK392948 | Hap_139 |
| 316 | AA-755 | ZSI_AA-755 | Tetragnathidae | *Tylorida ventralis* | 26.79 N 92.69 E | BOLD:ADF3786 | MK392949 | Hap_142 |
| 317 | AA-1007 | ZSI_AA-1007 | Tetragnathidae | *Tylorida ventralis* | 27.57 N 95.39 E | BOLD:ADF3786 | MK392950 | Hap_143 |
| 318 | AA-1014 | ZSI_AA-1014 | Tetragnathidae | *Tylorida ventralis* | 27.57 N 95.39 E | - | MK392951 | Hap_144 |
| 319 | AA-896 | ZSI_AA-896 | Tetragnathidae | *Tylorida ventralis* | 27.57 N 95.39 E | BOLD:ADF3786 | MK392952 | Hap_139 |
| 320 | AA-36 | ZSI_AA-36 | Uloboridae | *Zosis geniculata* | 23.41 N 87.11 E | BOLD:AAG8512 | MK392953 | Hap_145 |
| 321 | AA-35 | ZSI_AA-35 | Uloboridae | *Zosis geniculata* | 23.41 N 87.11 E | BOLD:AAG8512 | MK392954 | Hap_146 |
| 322 | AA-331 | ZSI_AA-331 | Uloboridae | *Zosis geniculata* | 22.12 N 88.73 E | BOLD:AAG8512 | MK392955 | Hap_146 |
| 323 | AA-157 | ZSI_AA-157 | Tetragnathidae | *Tetragnatha vermiformis* | 26.20 N 92.93 E | BOLD:ADA3000 | MK392956 | Hap_147 |
| 324 | AA-82 | ZSI_AA-82 | Tetragnathidae | *Tetragnatha mandibulata* | 23.12 N 83.20 E | BOLD:AAK2567 | MK392957 | Hap_148 |
| 325 | AA-370 | ZSI_AA-370 | Tetragnathidae | *Tetragnatha hasselti* | 27.47 N 94.91 E | BOLD:ADF3105 | MK392958 | Hap_149 |
| 326 | AA-888 | ZSI_AA-888 | Tetragnathidae | *Tetragnatha hasselti* | 27.57 N 95.33 E | BOLD:ADF3105 | MK392959 | Hap_150 |
| 327 | AA-133 | ZSI_AA-133 | Araneidae | *Nephilengys malabarensis* | 24.80 N 92.76 E | BOLD:AAX6709 | MK392960 | Hap_151 |
| 328 | AA-143 | ZSI_AA-143 | Araneidae | *Nephilengys malabarensis* | 24.80 N 92.76 E | BOLD:AAX6709 | MK392961 | Hap_151 |
| 329 | AA-134 | ZSI_AA-134 | Araneidae | *Nephilengys malabarensis* | 24.80 N 92.76 E | BOLD:AAX6709 | MK392962 | Hap_151 |
| 330 | AA-138 | ZSI_AA-138 | Araneidae | *Nephilengys malabarensis* | 24.80 N 92.76 E | BOLD:AAX6709 | MK392963 | Hap_151 |
| 331 | AA-1247 | ZSI_AA-1247 | Tetragnathidae | *Guizygiella* sp. | 23.22 N 72.67 E | BOLD:ABX7345 | MK392964 | Hap_152 |
| 332 | AA-587 | ZSI_AA-587 | Araneidae | *Parawixia dehaani* | 28.60 N 95.49 E | BOLD:ADC8499 | MK392965 | Hap_153 |
| 333 | AA-338 | ZSI_AA-338 | Araneidae | *Parawixia dehaani* | 22.16 N 88.82 E | BOLD:ADC8499 | MK392966 | Hap_154 |
| 334 | AA-550 | ZSI_AA-550 | Oecobiidae | *Oecobius putus* | 27.02 N 74.21 E | BOLD:ADH0037 | MK392967 | Hap_155 |
| 335 | AA-549 | ZSI_AA-549 | Oecobiidae | *Oecobius putus* | 27.02 N 74.21 E | BOLD:ADH0037 | MK392968 | Hap_155 |
| 336 | AA-691 | ZSI_AA-691 | Clubionidae | *Clubiona* sp. | 27.29 N 95.51 E | BOLD:ADI2347 | MK392969 | Hap_156 |
| 337 | AA-884 | ZSI_AA-884 | Clubionidae | *Clubiona* sp. | 27.57 N 95.33 E | BOLD:ADI2346 | MK392970 | Hap_157 |
| 338 | AA-821 | ZSI_AA-821 | Lycosidae | *Draposa* sp. | 27.66 N 95.36 E | BOLD:ADI2986 | MK392971 | Hap_158 |
| 339 | AA-488 | ZSI_AA-488 | Lycosidae | *Pardosa pusiola* | 26.20 N 92.93 E | BOLD:ACY2498 | MK392972 | Hap_159 |
| 340 | AA-482 | ZSI_AA-482 | Lycosidae | *Pardosa pusiola* | 26.20 N 92.93 E | BOLD:ACY2498 | MK392973 | Hap_159 |
| 341 | AA-495 | ZSI_AA-495 | Lycosidae | *Pardosa pusiola* | 26.20 N 92.93 E | BOLD:ACY2498 | MK392974 | Hap_159 |
| 342 | AA-512 | ZSI_AA-512 | Lycosidae | *Pardosa pusiola* | 26.74 N 94.28 E | BOLD:ACY2498 | MK392975 | Hap_159 |
| 343 | AA-523 | ZSI_AA-523 | Lycosidae | *Pardosa pusiola* | 26.20 N 92.93 E | BOLD:ACY2498 | MK392976 | Hap_159 |
| 344 | AA-481 | ZSI_AA-481 | Lycosidae | *Pardosa pusiola* | 26.20 N 92.93 E | BOLD:ACY2498 | MK392977 | Hap_159 |
| 345 | AA-503 | ZSI_AA-503 | Lycosidae | *Pardosa pusiola* | 26.20 N 92.93 E | BOLD:ACY2498 | MK392978 | Hap_159 |
| 346 | AA-483 | ZSI_AA-483 | Lycosidae | *Pardosa pusiola* | 26.20 N 92.93 E | BOLD:ACY2498 | MK392979 | Hap_159 |
| 347 | AA-487 | ZSI_AA-487 | Lycosidae | *Pardosa pusiola* | 27.72 N 95.65 E | BOLD:ACY2498 | MK392980 | Hap_160 |
| 348 | AA-492 | ZSI_AA-492 | Lycosidae | *Pardosa pusiola* | 27.72 N 95.65 E | BOLD:ACY2498 | MK392981 | Hap_160 |
| 349 | AA-472 | ZSI_AA-472 | Lycosidae | *Pardosa pusiola* | 26.20 N 92.93 E | BOLD:ACY2498 | MK392982 | Hap_159 |
| 350 | AA-489 | ZSI_AA-489 | Lycosidae | *Pardosa pusiola* | 26.20 N 92.93 E | BOLD:ACY2498 | MK392983 | Hap_159 |
| 351 | AA-516 | ZSI_AA-516 | Lycosidae | *Pardosa pusiola* | 22.90 N 72.46 E | BOLD:ADF3532 | MK392984 | Hap_161 |
| 352 | AA-1148 | ZSI_AA-1148 | Lycosidae | *Pardosa pusiola* | 20.77 N 73.67 E | BOLD:ADF3532 | MK392985 | Hap_162 |
| 353 | AA-1796 | ZSI_AA-729 | Lycosidae | *Pardosa pusiola* | 23.22 N 72.34 E | BOLD:ADF3532 | MK392986 | Hap_163 |
| 354 | AA-205 | ZSI_AA-205 | Lycosidae | *Pardosa pusiola* | 21.45 N 72.82 E | BOLD:ADF3532 | MK392987 | Hap_162 |
| 355 | AA-174 | ZSI_AA-174 | Lycosidae | *Pardosa sumatrana* | 26.68 N 92.81 E | BOLD:ADF4185 | MK392988 | Hap_164 |
| 356 | AA-476 | ZSI_AA-476 | Lycosidae | *Pardosa sumatrana* | 27.74 N 95.55 E | BOLD:ADI1399 | MK392989 | Hap_165 |
| 357 | AA-491 | ZSI_AA-491 | Lycosidae | *Pardosa sumatrana* | 27.74 N 95.55 E | BOLD:ADG5519 | MK392990 | Hap_166 |
| 358 | AA-486 | ZSI_AA-486 | Lycosidae | *Pardosa sumatrana* | 27.74 N 95.55 E | BOLD:ADG5519 | MK392991 | Hap_166 |
| 359 | AA-643 | ZSI_AA-643 | Lycosidae | *Pardosa sumatrana* | 26.68 N 92.81 E | BOLD:ADI2476 | MK392992 | Hap_167 |
| 360 | AA-518 | ZSI_AA-518 | Lycosidae | *Pardosa birmanica* | 26.20 N 92.93 E | BOLD:AAG7456 | MK392993 | Hap_168 |
| 361 | AA-519 | ZSI_AA-519 | Lycosidae | *Pardosa birmanica* | 26.20 N 92.93 E | BOLD:AAG7456 | MK392994 | Hap_169 |
| 362 | AA-853 | ZSI_AA-853 | Lycosidae | *Hogna himalayensis* | 27.66 N 95.36 E | BOLD:ADA1978 | MK392995 | Hap_170 |
| 363 | AA-152 | ZSI_AA-152 | Lycosidae | *Hogna himalayensis* | 27.66 N 95.36 E | BOLD:ADA1978 | MK392996 | Hap_170 |
| 364 | AA-1159 | ZSI_AA-1159 | Lycosidae | *Lycosa nigrotibialis* | 21.00 N 72.85 E | BOLD:ADG5677 | MK392997 | Hap_171 |
| 365 | AA-515 | ZSI_AA-515 | Lycosidae | *Lycosa nigrotibialis* | 22.90 N 72.46 E | BOLD:ADG5677 | MK392998 | Hap_172 |
| 366 | AA-776 | ZSI_AA-776 | Oxyopidae | *Hamataliwa* sp. | 27.52 N 95.36 E | BOLD:ADI2630 | MK392999 | Hap_173 |
| 367 | AA-845 | ZSI_AA-845 | Oxyopidae | *Hamataliwa* sp. | 27.66 N 95.36 E | BOLD:ADI2630 | MK393000 | Hap_174 |
| 368 | AA-1252 | ZSI_AA-1252 | Oxyopidae | *Hamataliwa* sp. | 22.40 N 72.99 E | BOLD:ADJ7430 | MK393001 | Hap_175 |
| 369 | AA-379 | ZSI_AA-379 | Oxyopidae | *Oxyopes birmanicus* | 26.67 N 92.85 E | BOLD:ACW8679 | MK393002 | Hap_176 |
| 370 | AA-430 | ZSI_AA-430 | Oxyopidae | *Oxyopes birmanicus* | 22.55 N 88.29 E | BOLD:ACW8679 | MK393003 | Hap_176 |
| 371 | AA-930 | ZSI_AA-930 | Oxyopidae | *Oxyopes birmanicus* | 27.52 N 95.36 E | BOLD:ACW8679 | MK393004 | Hap_176 |
| 372 | AA-931 | ZSI_AA-931 | Oxyopidae | *Oxyopes birmanicus* | 27.52 N 95.36 E | BOLD:ACW8679 | MK393005 | Hap_176 |
| 373 | AA-440 | ZSI_AA-440 | Oxyopidae | *Oxyopes birmanicus* | 23.17 N 85.98 E | BOLD:ACW8679 | MK393006 | Hap_176 |
| 374 | AA-420 | ZSI_AA-420 | Oxyopidae | *Oxyopes birmanicus* | 27.80 N 95.24 E | BOLD:ACW8679 | MK393007 | Hap_177 |
| 375 | AA-1039 | ZSI_AA-1039 | Oxyopidae | *Oxyopes birmanicus* | 27.52 N 95.36 E | BOLD:ACW8679 | MK393008 | Hap_178 |
| 376 | AA-415 | ZSI_AA-415 | Oxyopidae | *Oxyopes birmanicus* | 22.55 N 88.29 E | BOLD:ACW8679 | MK393009 | Hap_179 |
| 377 | AA-620 | ZSI_AA-620 | Oxyopidae | *Oxyopes birmanicus* | 26.77 N 92.96 E | BOLD:ADI4857 | MK393010 | Hap_176 |
| 378 | AA-618 | ZSI_AA-618 | Oxyopidae | *Oxyopes birmanicus* | 27.52 N 95.36 E | BOLD:ACW8679 | MK393011 | Hap_176 |
| 379 | AA-615 | ZSI_AA-615 | Oxyopidae | *Oxyopes birmanicus* | 27.52 N 95.36 E | BOLD:ACW8679 | MK393012 | Hap_176 |
| 380 | AA-625 | ZSI_AA-625 | Oxyopidae | *Oxyopes birmanicus* | 27.52 N 95.36 E | BOLD:ACW8679 | MK393013 | Hap_176 |
| 381 | AA-614 | ZSI_AA-614 | Oxyopidae | *Oxyopes birmanicus* | 27.52 N 95.36 E | BOLD:ACW8679 | MK393014 | Hap_176 |
| 382 | AA-611 | ZSI_AA-611 | Oxyopidae | *Oxyopes birmanicus* | 27.52 N 95.36 E | BOLD:ACW8679 | MK393015 | Hap_176 |
| 383 | AA-624 | ZSI_AA-624 | Oxyopidae | *Oxyopes birmanicus* | 27.52 N 95.36 E | BOLD:ACW8679 | MK393016 | Hap_176 |
| 384 | AA-613 | ZSI_AA-613 | Oxyopidae | *Oxyopes birmanicus* | 27.52 N 95.36 E | BOLD:ACW8679 | MK393017 | Hap_180 |
| 385 | AA-610 | ZSI_AA-610 | Oxyopidae | *Oxyopes birmanicus* | 27.52 N 95.36 E | BOLD:ACW8679 | MK393018 | Hap_176 |
| 386 | AA-915 | ZSI_AA-915 | Oxyopidae | *Oxyopes birmanicus* | 27.52 N 95.36 E | BOLD:ACW8679 | MK393019 | Hap_176 |
| 387 | AA-433 | ZSI_AA-433 | Oxyopidae | *Oxyopes birmanicus* | 22.55 N 88.29 E | BOLD:ACW8679 | MK393020 | Hap_176 |
| 388 | AA-429 | ZSI_AA-429 | Oxyopidae | *Oxyopes birmanicus* | 22.55 N 88.29 E | BOLD:ACW8679 | MK393021 | Hap_176 |
| 389 | AA-538 | ZSI_AA-538 | Oxyopidae | *Oxyopes birmanicus* | 22.21 N 88.32 E | BOLD:ACW8679 | MK393022 | Hap_176 |
| 390 | AA-699 | ZSI_AA-699 | Oxyopidae | *Oxyopes birmanicus* | 26.74 N 94.28 E | BOLD:ACW8679 | MK393023 | Hap_181 |
| 391 | AA-416 | ZSI_AA-416 | Oxyopidae | *Oxyopes birmanicus* | 22.55 N 88.29 E | BOLD:ACW8679 | MK393024 | Hap_176 |
| 392 | AA-442 | ZSI_AA-442 | Oxyopidae | *Oxyopes birmanicus* | 22.55 N 88.29 E | BOLD:ACW8679 | MK393025 | Hap_176 |
| 393 | AA-684 | ZSI_AA-684 | Oxyopidae | *Oxyopes birmanicus* | 27.52 N 95.36 E | BOLD:ACW8679 | MK393026 | Hap_176 |
| 394 | AA-926 | ZSI_AA-926 | Oxyopidae | *Oxyopes birmanicus* | 27.52 N 95.36 E | BOLD:ACW8679 | MK393027 | Hap_182 |
| 395 | AA-1799 | ZSI_AA-732 | Oxyopidae | *Oxyopes birmanicus* | 22.54 N 88.49 E | BOLD:ACW8679 | MK393028 | Hap_178 |
| 396 | AA-446 | ZSI_AA-446 | Oxyopidae | *Oxyopes birmanicus* | 22.55 N 88.29 E | BOLD:ACW8679 | MK393029 | Hap_176 |
| 397 | AA-685 | ZSI_AA-685 | Oxyopidae | *Oxyopes birmanicus* | 27.52 N 95.36 E | BOLD:ACW8679 | MK393030 | Hap_176 |
| 398 | AA-678 | ZSI_AA-678 | Oxyopidae | *Oxyopes birmanicus* | 27.52 N 95.36 E | BOLD:ACW8679 | MK393031 | Hap_183 |
| 399 | AA-941 | ZSI_AA-941 | Oxyopidae | *Oxyopes birmanicus* | 27.52 N 95.36 E | BOLD:ACW8679 | MK393032 | Hap_176 |
| 400 | AA-612 | ZSI_AA-612 | Oxyopidae | *Oxyopes birmanicus* | 27.52 N 95.36 E | BOLD:ADI4857 | MK393033 | Hap_184 |
| 401 | AA-880 | ZSI_AA-880 | Oxyopidae | *Oxyopes birmanicus* | 22.95 N 88.00 E | BOLD:ADI4857 | MK393034 | Hap_184 |
| 402 | AA-622 | ZSI_AA-622 | Oxyopidae | *Oxyopes birmanicus* | 26.74 N 94.28 E | BOLD:ADI4857 | MK393035 | Hap_184 |
| 403 | AA-675 | ZSI_AA-675 | Oxyopidae | *Oxyopes birmanicus* | 27.52 N 95.36 E | BOLD:ADI4857 | MK393036 | Hap_185 |
| 404 | AA-940 | ZSI_AA-940 | Oxyopidae | *Oxyopes birmanicus* | 26.77 N 92.96 E | BOLD:ADI4857 | MK393037 | Hap_185 |
| 405 | AA-933 | ZSI_AA-933 | Oxyopidae | *Oxyopes birmanicus* | 26.77 N 92.96 E | BOLD:ADI4857 | MK393038 | Hap_186 |
| 406 | AA-771 | ZSI_AA-771 | Oxyopidae | *Oxyopes birmanicus* | 27.52 N 95.36 E | BOLD:ADI4857 | MK393039 | Hap_184 |
| 407 | AA-616 | ZSI_AA-616 | Oxyopidae | *Oxyopes birmanicus* | 27.52 N 95.36 E | BOLD:ADI4857 | MK393040 | Hap_187 |
| 408 | AA-953 | ZSI_AA-953 | Oxyopidae | *Oxyopes birmanicus* | 26.77 N 92.96 E | BOLD:ADI4857 | MK393041 | Hap_188 |
| 409 | AA-676 | ZSI_AA-676 | Oxyopidae | *Oxyopes birmanicus* | 26.77 N 92.96 E | BOLD:ADI4857 | MK393042 | Hap_189 |
| 410 | AA-1035 | ZSI_AA-1035 | Oxyopidae | *Oxyopes birmanicus* | 26.77 N 92.96 E | BOLD:ADI4857 | MK393043 | Hap_190 |
| 411 | AA-932 | ZSI_AA-932 | Oxyopidae | *Oxyopes birmanicus* | 27.65 N 95.47 E | BOLD:ADI4090 | MK393044 | Hap_191 |
| 412 | AA-1049 | ZSI_AA-1049 | Oxyopidae | *Oxyopes birmanicus* | 27.65 N 95.47 E | BOLD:ADI4090 | MK393045 | Hap_191 |
| 413 | AA-621 | ZSI_AA-621 | Oxyopidae | *Oxyopes birmanicus* | 27.52 N 95.36 E | BOLD:ADI4857 | MK393046 | Hap_192 |
| 414 | AA-920 | ZSI_AA-920 | Oxyopidae | *Oxyopes birmanicus* | 26.77 N 92.96 E | BOLD:ADI4857 | MK393047 | Hap_185 |
| 415 | AA-1022 | ZSI_AA-1022 | Oxyopidae | *Oxyopes birmanicus* | 26.77 N 92.96 E | BOLD:ADI4857 | MK393048 | Hap_185 |
| 416 | AA-425 | ZSI_AA-425 | Oxyopidae | *Oxyopes hindostanicus* | 22.55 N 88.29 E | BOLD:AAO1990 | MK393049 | Hap_193 |
| 417 | AA-385 | ZSI_AA-385 | Oxyopidae | *Oxyopes javanus* | 26.67 N 92.85 E | BOLD:ACW9232 | MK393050 | Hap_194 |
| 418 | AA-380 | ZSI_AA-380 | Oxyopidae | *Oxyopes javanus* | 26.67 N 92.85 E | BOLD:ACW9232 | MK393051 | Hap_195 |
| 419 | AA-358 | ZSI_AA-358 | Oxyopidae | *Oxyopes javanus* | 27.47 N 94.91 E | BOLD:ACW9232 | MK393052 | Hap_196 |
| 420 | AA-619 | ZSI_AA-619 | Oxyopidae | *Oxyopes javanus* | 27.52 N 95.36 E | BOLD:ACW9232 | MK393053 | Hap_196 |
| 421 | AA-964 | ZSI_AA-964 | Oxyopidae | *Oxyopes javanus* | 27.52 N 95.36 E | BOLD:ACW9232 | MK393054 | Hap_197 |
| 422 | AA-766 | ZSI_AA-766 | Oxyopidae | *Oxyopes javanus* | 27.52 N 95.36 E | BOLD:ACW9232 | MK393055 | Hap_196 |
| 423 | AA-763 | ZSI_AA-763 | Oxyopidae | *Oxyopes javanus* | 27.52 N 95.36 E | BOLD:ACW9232 | MK393056 | Hap_196 |
| 424 | AA-946 | ZSI_AA-946 | Oxyopidae | *Oxyopes javanus* | 27.52 N 95.36 E | BOLD:ACW9232 | MK393057 | Hap_196 |
| 425 | AA-830 | ZSI_AA-830 | Oxyopidae | *Oxyopes javanus* | 27.66 N 95.36 E | BOLD:ACW9232 | MK393058 | Hap_198 |
| 426 | AA-617 | ZSI_AA-617 | Oxyopidae | *Oxyopes javanus* | 27.52 N 95.36 E | BOLD:ACW9232 | MK393059 | Hap_199 |
| 427 | AA-623 | ZSI_AA-623 | Oxyopidae | *Oxyopes javanus* | 27.52 N 95.36 E | BOLD:ACW9232 | MK393060 | Hap_200 |
| 428 | AA-848 | ZSI_AA-848 | Oxyopidae | *Oxyopes javanus* | 27.66 N 95.36 E | BOLD:ACW9232 | MK393061 | Hap_196 |
| 429 | AA-1807 | ZSI_AA-778 | Oxyopidae | *Oxyopes javanus* | 27.52 N 95.36 E | BOLD:ACW9232 | MK393062 | Hap_201 |
| 430 | AA-1136 | ZSI_AA-1136 | Oxyopidae | *Oxyopes javanus* | 21.00 N 72.85 E | BOLD:ACW9232 | MK393063 | Hap_202 |
| 431 | AA-935 | ZSI_AA-935 | Oxyopidae | *Oxyopes javanus* | 27.52 N 95.36 E | BOLD:ACW9232 | MK393064 | Hap_203 |
| 432 | AA-844 | ZSI_AA-844 | Oxyopidae | *Oxyopes javanus* | 27.66 N 95.36 E | BOLD:ACW9232 | MK393065 | Hap_204 |
| 433 | AA-836 | ZSI_AA-836 | Oxyopidae | *Oxyopes javanus* | 27.66 N 95.36 E | BOLD:ACW9232 | MK393066 | Hap_196 |
| 434 | AA-832 | ZSI_AA-832 | Oxyopidae | *Oxyopes javanus* | 27.66 N 95.36 E | BOLD:ACW9232 | MK393067 | Hap_205 |
| 435 | AA-373 | ZSI_AA-373 | Oxyopidae | *Oxyopes shweta* | 26.67 N 92.85 E | BOLD:AAF9665 | MK393068 | Hap_206 |
| 436 | AA-886 | ZSI_AA-886 | Oxyopidae | *Oxyopes shweta* | 27.57 N 95.33 E | BOLD:AAF9665 | MK393069 | Hap_207 |
| 437 | AA-677 | ZSI_AA-677 | Oxyopidae | *Oxyopes shweta* | 27.52 N 95.36 E | BOLD:AAF9665 | MK393070 | Hap_208 |
| 438 | AA-867 | ZSI_AA-867 | Oxyopidae | *Oxyopes shweta* | 27.57 N 95.33 E | BOLD:AAF9665 | MK393071 | Hap_209 |
| 439 | AA-426 | ZSI_AA-426 | Thomisidae | *Camaricus formosus* | 22.55 N 88.29 E | BOLD:ADG6782 | MK393072 | Hap_210 |
| 440 | AA-423 | ZSI_AA-423 | Thomisidae | *Camaricus formosus* | 22.55 N 88.29 E | BOLD:ADG6782 | MK393073 | Hap_210 |
| 441 | AA-768 | ZSI_AA-768 | Thomisidae | *Camaricus formosus* | 27.52 N 95.36 E | BOLD:ADG6782 | MK393074 | Hap_211 |
| 442 | AA-963 | ZSI_AA-963 | Thomisidae | *Camaricus formosus* | 27.52 N 95.36 E | BOLD:ADG6782 | MK393075 | Hap_211 |
| 443 | AA-569 | ZSI_AA-569 | Thomisidae | *Thomisus unidentatus* | 24.59 N 72.72 E | BOLD:AAP4819 | MK393076 | Hap_212 |
| 444 | AA-566 | ZSI_AA-566 | Thomisidae | *Thomisus unidentatus* | 24.59 N 72.72 E | BOLD:AAP4819 | MK393077 | Hap_213 |
| 445 | AA-567 | ZSI_AA-567 | Thomisidae | *Thomisus unidentatus* | 24.59 N 72.72 E | BOLD:AAP4819 | MK393078 | Hap_214 |
| 446 | AA-1251 | ZSI_AA-1251 | Thomisidae | *Thomisus unidentatus* | 22.40 N 72.99 E | BOLD:AAP4819 | MK393079 | Hap_214 |
| 447 | AA-564 | ZSI_AA-564 | Thomisidae | *Thomisus unidentatus* | 24.59 N 72.72 E | BOLD:AAP4819 | MK393080 | Hap_215 |
| 448 | AA-565 | ZSI_AA-565 | Thomisidae | *Thomisus unidentatus* | 24.59 N 72.72 E | BOLD:AAP4819 | MK393081 | Hap_215 |
| 449 | AA-586 | ZSI_AA-586 | Psechridae | *Psechrus inflatus* | 24.87 N 92.86 E | BOLD:ADH1543 | MK393082 | Hap_216 |
| 450 | AA-605 | ZSI_AA-605 | Psechridae | *Psechrus inflatus* | 28.60 N 95.49 E | BOLD:ADH1543 | MK393083 | Hap_216 |
| 451 | AA-705 | ZSI_AA-705 | Thomisidae | *Synema* sp. | 27.29 N 95.51 E | BOLD:ADI2068 | MK393084 | Hap_217 |
| 452 | AA-727 | ZSI_AA-727 | Thomisidae | *Synema* sp. | 27.29 N 95.51 E | BOLD:ADI2068 | MK393085 | Hap_217 |
| 453 | AA-901 | ZSI_AA-901 | Salticidae | *Bavia* sp. | 27.52 N 95.36 E | BOLD:ADI2712 | MK393086 | Hap_218 |
| 454 | AA-435 | ZSI_AA-435 | Salticidae | *Evarcha flavocincta* | 22.55 N 88.29 E | BOLD:ADG5335 | MK393087 | Hap_219 |
| 455 | AA-439 | ZSI_AA-439 | Salticidae | *Evarcha flavocincta* | 22.55 N 88.29 E | BOLD:ADG5335 | MK393088 | Hap_220 |
| 456 | AA-342 | ZSI_AA-342 | Hersiliidae | *Hersilia savignyi* | 22.12 N 88.73 E | BOLD:ACZ0025 | MK393089 | Hap_221 |
| 457 | AA-343 | ZSI_AA-343 | Hersiliidae | *Hersilia savignyi* | 27.46 N 95.19 E | BOLD:ACZ0025 | MK393090 | Hap_222 |
| 458 | AA-347 | ZSI_AA-347 | Hersiliidae | *Hersilia savignyi* | 27.46 N 95.19 E | BOLD:ACZ0025 | MK393091 | Hap_222 |
| 459 | AA-346 | ZSI_AA-346 | Hersiliidae | *Hersilia savignyi* | 27.46 N 95.19 E | BOLD:ACZ0025 | MK393092 | Hap_222 |
| 460 | AA-340 | ZSI_AA-340 | Hersiliidae | *Hersilia savignyi* | 27.46 N 95.19 E | BOLD:ACZ0025 | MK393093 | Hap_222 |
| 461 | AA-329 | ZSI_AA-329 | Hersiliidae | *Hersilia savignyi* | 27.46 N 95.19 E | BOLD:ACZ0025 | MK393094 | Hap_222 |
| 462 | AA-327 | ZSI_AA-327 | Hersiliidae | *Hersilia savignyi* | 27.46 N 95.19 E | BOLD:ACZ0025 | MK393095 | Hap_223 |
| 463 | AA-41 | ZSI_AA-41 | Hersiliidae | *Hersilia savignyi* | 27.46 N 95.19 E | BOLD:ACZ0025 | MK393096 | Hap_224 |
| 464 | AA-563 | ZSI_AA-563 | Araneidae | *Eriovixia excelsa* | 24.59 N 72.72 E | BOLD:AAQ0105 | MK393097 | Hap_225 |
| 465 | AA-1156 | ZSI_AA-1156 | Nemesiidae | *Damarchus* sp. | 20.77 N 73.67 E | BOLD:ADM0082 | MK393098 | Hap_226 |
| 466 | AA-14 | ZSI_AA-14 | Pholcidae | *Artema atlanta* | 22.85N 88.53 E | BOLD:AAJ9862 | MK393099 | Hap_227 |
| 467 | AA-39 | ZSI_AA-39 | Pholcidae | *Artema atlanta* | 23.41 N 87.11 E | BOLD:AAJ9862 | MK393100 | Hap_228 |
| 468 | AA-1795 | ZSI_AA-728 | Pholcidae | *Crossopriza lyoni* | 27.29 N 95.51 E | BOLD:AAG2795 | MK393101 | Hap_229 |
| 469 | AA-328 | ZSI_AA-328 | Pholcidae | *Crossopriza lyoni* | 22.18 N 88.73 E | BOLD:AAG2795 | MK393102 | Hap_230 |
| 470 | AA-73 | ZSI_AA-73 | Pholcidae | *Micropholcus fauroti* | 22.85 N 88.53 E | BOLD:AAL8196 | MK393103 | Hap_231 |
| 471 | AA-47 | ZSI_AA-47 | Pholcidae | *Micropholcus fauroti* | 22.81 N 88.53 E | BOLD:AAL8196 | MK393104 | Hap_231 |
| 472 | AA-509 | ZSI_AA-509 | Theraphosidae | *Lyrognathus crotalus* | 26.20 N 92.93 E | BOLD:ADI3945 | MK393105 | Hap_232 |
| 473 | AA-419 | ZSI_AA-419 | Scytodidae | *Scytodes thoracica* | 27.43 N 95.50 E | BOLD:ADG4510 | MK393106 | Hap_233 |
| 474 | AA-427 | ZSI_AA-427 | Scytodidae | *Scytodes thoracica* | 27.43 N 95.50 E | BOLD:ADG4510 | MK393107 | Hap_234 |
| 475 | AA-432 | ZSI_AA-432 | Scytodidae | *Scytodes thoracica* | 22.55 N 88.29 E | BOLD:ADG4510 | MK393108 | Hap_234 |
| 476 | AA-642 | ZSI_AA-642 | Lycosidae | *Pardosa pseudoannulata* | 27.87 N 95.67 E | BOLD:AAO2149 | MK393109 | Hap_235 |
| 477 | AA-1145 | ZSI_AA-1145 | Lycosidae | *Pardosa pseudoannulata* | 20.77 N 73.67 E | - | MK393110 | Hap_236 |
| 478 | AA-662 | ZSI_AA-662 | Salticidae | *Carrhotus* sp. | 27.52 N 95.36 E | BOLD:ADI4507 | MK393111 | Hap_237 |
| 479 | AA-663 | ZSI_AA-663 | Salticidae | *Carrhotus* sp. | 27.52 N 95.36 E | BOLD:ADI4507 | MK393112 | Hap_238 |
| 480 | AA-689 | ZSI_AA-689 | Zodariidae | *Mallinella* sp. | 27.29 N 95.51 E | BOLD:ADI3053 | MK393113 | Hap_239 |
| 481 | AA-532 | ZSI_AA-532 | Scytodidae | *Scytodes fusca* | 22.81 N 88.53 E | BOLD:AAG5665 | MK393114 | Hap_240 |
| 482 | AA-719 | ZSI_AA-719 | Psechridae | *Fecenia protensa* | 27.29 N 95.51 E | BOLD:ADI3576 | MK393115 | Hap_241 |
| 483 | AA-927 | ZSI_AA-927 | Thomisidae | *Indoxysticus* sp. | 27.52 N 95.36 E | BOLD:ADI1602 | MK393116 | Hap_242 |
| 484 | AA-1794 | ZSI_AA-1794 | Oxyopidae | *Oxyopes sakuntalae* | 27.29 N 95.51 E | - | MK393117 | Hap_243 |
| 485 | AA-1375 | ZSI_AA-1375 | Thomisidae | *Epidius parvati* | 11.27 N 75.79 E | BOLD:ADN9722 | MK393118 | Hap_244 |
| 486 | AA-1429 | ZSI_AA-1429 | Thomisidae | *Epidius parvati* | 10.53 N 76.36 E | BOLD:ADN9722 | MK393119 | Hap_244 |
| 487 | AA-1365 | ZSI_AA-1365 | Lycosidae | *Pardosa procurva* | 13.01 N 77.56 E | BOLD:ADN1098 | MK393120 | Hap_245 |
| 488 | AA-1424 | ZSI_AA-1424 | Salticidae | *Chalcotropis pennata* | 10.53 N 76.36 E | BOLD:ADO5716 | MK393121 | Hap_246 |
| 489 | AA-1366 | ZSI_AA-1366 | Theridiidae | *Meotipa sahyadri* | 13.01 N 77.56 E | BOLD:ADN2342 | MK393122 | Hap_247 |

**Table S2:** Morphological characters and bibliography for accurate identifications of the studied spider species.

| **Sl. No.** | **Species** | **Characters** | **Citation** |
| --- | --- | --- | --- |
| 1 | *Araneus mitificus* | Cephalothorax greenish-yellow; abdomen globular, silvery, with a pair of wing shaped median black patch and two lateral black patches; epigyne with very short, thick, unwrinkled scape. | Tikader 1982 |
| 2 | *Argiope versicolor* | Female with longer and narrower internal duct system than *Argiope pulchella* and broken off embolus part is longer in this species. | Levi 1983; Jäger & Praxaysombath 2009 |
| 3 | *Argiope pulchella* | Abdomen yellowish white, pentagonal with 3 pairs of conspicuous sigilla, ventral side of the abdomen dark brown with a pair of chalk white longitudinal patches extending from epigastric furrow to the spinnerets; epigyne with a small anterior bulge with thicker U-shaped rim | Tikader 1982; Levi 1983 |
| 4 | *Cyclosa bianchoria* | Epigynal scape folded, u-shaped in lateral view, wrinkled in the proximal portion; the distal end of the scape is not wrinkled. | Yin et al. 1990 |
| 5 | *Cyclosa mulmeinensis* | Abdomen globular with one pair of distinct tubercles; epigyne with rounded scape, spermathecae L-shaped posterolaterally. | Tikader 1982 |
| 6 | *Cyclosa bifida* | Abdomen elongated with a blunt caudal hump with a pair of silvery patches longitudinally and three pairs of distinct sigilla mid- longitudinally; epigynal scape long, tapering posteriorly; spermathecae bean shaped. | Tikader 1982 |
| 7 | *Cyclosa spirifera* | Abdomen with two median humps, presence of one pair of lateral humps at the base of the caudal hump; epigyne with wrinkled scape and a bent tip, scape with a deep constriction anteriorly. | Tikader 1982 |
| 8 | *Cyrtophora cicatrosa* | Abdomen with two pairs of small black tubercles anteriorly and posteriorly with a blunt caudal tubercle; epigyne inconspicuous, spermathecae pear shaped. | Tikader 1982 |
| 9 | *Chorizopes quadrituberculata* | Abdomen sub-rectangular, dorsum with two pairs of lateral tubercles, posterior end projected outwards; a creamy white mid-longitudinal patch; epigyne semilunar, spermatheca kidney shaped. | Roy, Sen, Saha & Raychaudhuri 2014 |
| 10 | *Eriovixia poonaensis* | Abdomen pointed posteriorly without a hump-like tail unlike *E. laglaizei*; epigyne having broad scape, copulatory openings placed on posterior surface, copulatory ducts twisted near the openings; spermathecae spherical. | Tikader 1982;  Mi & Wang, 2016 |
| 11 | *Eriovixia excelsa* | Posterior end of abdomen with a small tubercle. Epigyne with a short triangular scape; short curved copulatory ducts and elliptical spermathecae. | Tikader 1982  Mi, Peng & Yin 2010 |
| 12 | *Gasteracantha diadesmia* | Abdomen wide, consists of three transverse yellowish chalk-white bands and three pairs of spines, of which median spine is largest; epigyne bicleft, with a short scape. | Tikader 1982; Barrion & Litsinger 1995 |
| 13 | *Gasteracantha hasselti* | Abdomen chalk white with blackish brown outer sides, median spines reduced and not exceeding half the width of the abdomen; spermatheca of this species is smaller and spherical | Tikader 1982; Tan *et al.* 2019 |
| 14 | *Gasteracantha kuhli* | Abdomen with yellowish white patches and a few pairs of large sigilla, median and posterior spines subequal in size and larger than the anterior spine; epigyne with a projecting, short, and triangularly pointed scape. | Tikader 1982; Barrion & Litsinger 1995 |
| 15 | *Neoscona nautica* | Abdomen triangular with six pairs of sigilla arranged mid-longitudinally; epigynal scape triangular, consisting of a pair of lateral lobes and without constriction. | Tikader 1982 |
| 16 | *Neoscona theisi* | Abdomen suboval, elongate, with mid-longitudinal chevron-shaped markings; epigynal scape with prominent rim and constriction at the middle and consists of a pair of lateral lobes. | Tikader 1982; Barrion & Litsinger 1995 |
| 17 | *Nephila pilipes* | Abdomen cylindrical, elongate, blackish with a pair of yellow stripes; epigyne sclerotized with a transverse concave groove; copulatory ducts short; spermatheca ovoid. | Tikader 1982; Harvey, Austin & Adams 2007 |
| 18 | *Nephilengys malabarensis* | Abdomen greyish-yellow with brown patches laterally; epigyne heavily sclerotized with a pair of large depressions separated by a distinct median septum. | Tikader 1982 |
| 19 | *Parawixia dehaani* | Abdomen dark brown, triangular with two pointed spine on shoulder humps and one pointed tail hump at the posterior end; epigyne with a stout beak like scape, bent at right angle. | Tikader 1982; Barrion & Litsinger 1995 |
| 20 | *Clubiona* sp. 1 | Yellow, brown species, thoracic groove present; posterior eye row wider than anterior row, posterior median eyes closer to posterior lateral eyes than to each other; leg IV longer than leg I. | Majumder & Tikader 1991; Barrion & Litsinger 1995; Deeleman-Reinhold 2001 |
| 21 | *Deinopis* sp. | Cephalothorax elongate with narrow cephalic region; anterior lateral eyes on tubercles, posterior median eyes largest, extremely enlarged, placed less than a diameter apart; abdomen cylindrical and elongate. | Coddington, Kuntner & Opell 2012 |
| 22 | *Cheiracanthium triviale* | Female vulva with strongly convoluted insemination ducts which form 10 entwined loops, and the atrium is represented by clearly defined elongate-oval aperture. | Dankittipakul & Beccaloni 2012 |
| 23 | *Hersilia savignyi* | Male palps with short cymbium, tegular apophysis with a strongly projecting basal process, upper surface with pilose field on the latero-apical process; females with well sclerotized medially situated copulatory openings; narrow and elongate vulva, circular spermathecae. | Baehr and Baehr 1993, Caleb *et al*. 2017b |
| 24 | *Linyphia sikkimensis* | Cephalothorax brownish-yellow; abdomen chalk white, anterior part of with two longitudinal black lines and posteriorly with two transverse black patches; epigyne with large opening; atria with spirally coiled groove in the rigid wall. | Tikader 1970 |
| 25 | *Draposa* sp. | Carapace brownish with light brown to yellowish median and lateral bands; anterior medinals slightly larger than anterior laterals; labium slightly wider than long. | Kronestedt 2010 |
| 26 | *Hogna himalayensis* | Epigyne with deep atrium, median septum broad, inverted ‘T’ shaped, devoid of guide pocket, copulatory openings anteriorly placed to the transverse margin of median septum, spermatheca small and globular. | Tikader & Malhotra 1980;  Buchar 1997 |
| 27 | *Lycosa nigrotibialis* | Both the extremity of femur IV with a dark brown patch; the arch of epigyne is pointed posteriorly at the middle; septum inverted T-shaped. | Tikader & Malhotra 1980 |
| 28 | *Pardosa birmanica* | Abdomen dark with white spots and dorsal transverse patches. Epigyne concave and truncated posteromedially; having two semiround posterolateral lobes; median septum narrow. | Buchar 1976;  Barrion & Litsinger 1995 |
| 29 | *Pardosa pusiola* | Male palp with bifid terminal apophysis and prolaterally located subtegulum; female epigyne with a much wide base of septum; rounded spermathecae. | Wang & Zhang 2014 |
| 30 | *Pardosa sumatrana* | Epigyne with an inverted T-shaped median septum, transverse section almost twice as long as the vertical section; male palps have median apophysis with a short process. | Buchar 1980; Barrion & Litsinger 1995 |
| 31 | *Damarchus* sp. | Male tibia I with stout apophysis and metatarsus I with basoventral process composed of numerous denticles | Gravely 1921; Zonstein & Marusik 2014 |
| 32 | *Oecobius putus* | Epigyne with a sclerotized wedge-shaped plate, the lower sclerite lamella is long. Male palp with long terminal apophysis, the subterminal apophyses I is long and II is broad blade shaped. | Tikader 1962;  Kullmann & Zimmermann 1976 |
| 33 | *Hamataliwa* sp. 1 | Carapace rectangular in lateral view; clypeus and femora devoid of any black stripes; abdomen without geometric pattern. | Deeleman-Reinhold 2009 |
| 34 | *Oxyopes birmanicus* | Male palp with a thin and bent process in the tegular apophysis and two retrolateral pockets in the palpal tibia; Epigyne with a broad U-shaped posterior chitinous wall and two spherical structure of the internal duct system. | Jäger & Praxaysombath 2009 |
| 35 | *Oxyopes shweta* | Cephalothorax reddish-brown; abdomen with a lance-shaped brown patch, Ventral side of the abdomen chalk-white and the middle provided with a longitudinal brown line extending from epigastric fold to spinnerets. Epigyne as illustrated in Tikader (1970). | Tikader 1970 |
| 36 | *Oxyopes javanus* | Females with V-shaped epigyne, with bluntly rounded posterior margin, and anteriorly placed spermathecae, Male palps with thick, long RTA projecting retrolaterally. | Sherriffs 1951 |
| 37 | *Oxyopes hindostanicus* | Male palpal patella bearing two apophyses, the distal long and sharp and the basal blunt. | Pocock 1901; Sherriffs 1951 |
| 38 | *Artema atlanta* | Epigynal plate with two sclerotized lateral areas with distinctive pair of anterior epigynal projections. The posterior margin strongly indented. | Aharon, Huber & Gavish-Regev 2017 |
| 39 | *Crossopriza lyoni* | Females with a pair of protuberances on posterior side of prosoma and a pair of sclerotized plates on opisthosoma; Epigyne with a tooth like projection in the middle; posterior border of epigyne curved. | Tikader & Biswas 1981, Deeleman-Reinhold & van Harten 2001 |
| 40 | *Micropholcus fauroti* | Females with protruding epigyne; atrium large, anteriorly with a saddle shaped sclerite and posteriorly with a pair of oval plates. | Deeleman-Reinhold & Prinsen 1987 |
| 41 | *Fecenia protensa* | Male palp with long and narrow embolus; conductor arising at 12’o clock position and its tip directed at 1’ o clock position; median apophysis large and placed more or less centrally; retrolateral tibial apophysis stout and more or less club-shaped. | Malamel, Pradeep & Sebastian 2013 |
| 42 | *Psechrus inflatus* | Male palp with complex, meandering sperm duct; presence of macrosetae in the ventro-proximal region of palpal femur. | Bayer 2012 |
| 43 | *Bavia* sp. | Carapace low and broad, relatively flat dorsally; chelicerae with 4 teeth on promargin and 7 small teeth on retormargin; leg I robust with thick and long spines; abdomen long and narrowing posteriorly. | Berry, Beatty & Prószyński 1997; Prószyński & Deeleman- Reinhold 2013 |
| 44 | *Carrhotus* sp. | Epigyne with silt-shaped copulatory openings, pair of pockets along the epigastric furrow, copulatory ducts short and broad, spermathecae globular | Cao, Li & Zabka 2016 |
| 45 | *Evarcha flavocincta* | Epigyne with two depressions, and divided by a wide ridge; two small pockets present on either sides of the epigastric furrow; internal structures are complex with sclerotized canal forming numerous loops. | Żabka 1985 |
| 46 | *Epocilla sirohi* | Embolus long, broad at base, slender and tapering toward the tip, arising from three o’clock position with tip at 11 o’clock position, touching retrolateral margin of cymbium; palpal tibia enlarged retrolaterally; RTA short, curving inward; VTA broad. | Caleb *et al*. 2017a |
| 47 | *Hasarius adansoni* | Abdomen anteriorly fringed with light  grey hairs, with a longitudinal median belt formed by orange spots; males with short embolus extending obliquely over the bulbus; epigyne simple, sclerotized, appearing as two darker spots with translucent spermathecae. | Żabka 1985 |
| 48 | *Menemerus nigli* | Embolus relatively long, thin, with large membranous conductor; bulbus elongated ovoid, elongated; palpal tibia with porlateral enlargement; retrolateral apophysis straight. | Wesołowska & Freudenschuss 2012 |
| 49 | *Menemerus bivittatus* | Abdomen generally light, with brown margin; epigyne big with a pair of large depressions, partially divided by a medina septum; copulatory openings semicircular; copulatory ducts broad and very short; spermathecae oval. | Jastrzębski 1997a, Wesolowska 1999 |
| 50 | *Mogrus rajasthanensis* | Epigyne with longitudinal grooves on the lateral margins leading to the postero-lateral copulatory openings; copulatory ducts make V-shaped bend and rise inwards leading to the spermathecae. | Caleb *et al.* 2017a |
| 51 | *Myrmarachne kiboschensis* | Male chelicerae with 6 teeth externally and 5 teeth internally; embolus encircle the whole bulbus; tibial apophysis conical and robust. | Prószyński 1992 |
| 52 | *Pancorius magnus* | Abdomen dark brown with yellowish spots forming herring-bone pattern; males having oval bulbus with a translucent seminal reservoir, embolus short; tibial apophysis about one third of the bulbus; females with double chambered spermathecae. | Żabka 1985 |
| 53 | *Phintella accentifera* | Abdomen covered with grey and grey-brown setae having a metallic lustre; epigyne with large copulatory openings, copulatory ducts almost parallel, placed close to each other along the median axis, spermathecae oval, large. | Żabka 1985 |
| 54 | *Phintella vittata* | Abdomen light grey with two dark grey transverse streaks; epigyne strongly sclerotized with wide w-shaped posterior protrusion, copulatory canal diverging in a v-shaped manner; copulatory openings sclerotized. | Żabka 1985; Prószyński 1992 |
| 55 | *Phintella versicolor* | Cephalothorax grey-brown, having a blue metallic lusture; palp dark grey, with a double lateral apophysis; embolus short and thin. | Żabka 1985 |
| 56 | *Plexippus petersi* | Presence of wider and contrasting thoracic and abdominal median stripes; embolus and tibial apophysis distinctly longer; prolateral tegular margin distinctly serrated; epigyne long and narrow, copulatory openings crevices v-shaped, central pocket present anteriorly. | Żabka & Gardzińska 2017 |
| 57 | *Plexippus paykulli* | Cephalothorax brown with yellow longitudinal median thoracic stripe; male palp with embolus sabre-like set at antero-prolateral tegular corner, tibial apophysis short reaching half of tegulum height; epigyne large, copulatory openings creviced, central pocket present posteriorly. | Żabka & Gardzińska 2017 |
| 58 | *Rhene flavicomans* | Cephalothorax dark brown; abdomen brown with dense hairs; epigyne with horizontal, semicircular crevices; copulatory ducts pipe like with accessory glands; spermathecae elongate. | Jastrzębski 1997b |
| 59 | *Siler semiglaucus* | Carapace with dorsal red and lateral blue stripe; abdomen covered with two blue spots; female epigyne with anterior hood; transverse oval copulatory openings; copulatory duct broadening as it reaches the spermathecae. | Kulkarni & Joseph 2015 |
| 60 | *Thiania bhamoensis* | Abdomen slender blackish-grey with iridescent scales, usually blue or violet in colour; embolus dagger shaped, apophysis smaller and serrated on the internal edge; epigyne with two light brown depressions divided by a ridge; copulatory canals short; spermathecae pear-shaped. | Żabka 1985 |
| 61 | *Telamonia dimidiata* | Abdomen elongate and tapering with a pair of longitudinal deep brown bands; palp with a flap-shaped extension, tibial apophysis long and flattened, slight serrated apically, embolus long and thin. | Próchniewicz 1990 |
| 62 | *Yaginumaella incognita* | Abdomen brown with mosaic of whitish yellow spots; epigyne with a pair of pockets placed below the copulatory openings, copulatory canals long and coiled. | Zabka 1981 |
| 63 | *Heteropoda venatoria* | Male carapace with a black V-shaped band; tibial apophysis with two teeth like projection; course of the spermophor with a large bend at a tight angle in distal half of tegulum; epigyne with lateral lobes touching each other medially. | Jäger 2014 |
| 64 | *Pseudopoda cheppe* | Distal part of prosoma light yellow, opisthosoma reddish-brown with triangular creamy patch in posterior half and lateral black patches; embolus arising from 10-11 o’clock position on tegulum, sickle shaped; RTA single, curving uniformly in retrolateral view. | Caleb, Mondal & Kumar 2018 |
| 65 | *Sinopoda* sp. | Epigyne with modified rims, copulatory openings covered by laterally running ledges from medio-anterior to latero-posterior position, copulatory ducts uncoiled and touching each other. | Jäger 1999 |
| 66 | *Scytodes fusca* | Females with peculiar vulva in which the tubular proximal part of the receptacular stalk has thick, heavily sclerotized and perforated walls and receptacular head is membranous. | Dankittipakul & Singtripop 2010 |
| 67 | *Guizygiella* sp. | Anterior eyes row equal spaced, distance between posterior median eyes smaller than between median and lateral eyes, chelicerae without lateral condyles; abdomen oval, with black and white patches. | Zhu 1997 |
| 68 | *Leucauge xiaoen* | Carapace yellow, abdomen silver white, the longitudinal stripe in the middle of the ventral side of the abdomen is similar to *L. decorata*, but the dorsal side is different from the latter, a pair of light spot is present at the end of the abdomen. Epigynal median septum (inverted T-shaped), narrow anteriorly and wide posteriorly. | Zhu, Song & Zhang 2003 |
| 69 | *Leucauge tessellata* | Cephalothorax light brown; abdomen elongated with two blunt rounded  prominences and a blunt caudal hump at the posterior end; tibia IV with bottle-brush shaped hairs; epigynal plate having a thin semilunar rim. | Tikader 1982; Zhu, Song & Zhang 2003 |
| 70 | *Tylorida ventralis* | Chelicerae retromargin basally with a large rounded tubercle, basal promarginal tooth with swollen base and pro margin basally with a tiny tubercle. | Tikader 1982; Sankaran *et al*. 2017 |
| 71 | *Opadometa fastigata* | Cephalothorax yellowish brown; abdomen silvery white and orange-red with black markings, ventral surface with three pairs of silvery-white spots; epigyne with a recurved anterior margin anterior, lateral edge of septum is nearly parallel in posterior half. | Tikader 1982; Zhu, Song & Zhang 2003 |
| 72 | *Orsinome vethi* | Female epigyne with medially placed copulatory openings; coiled and well sclerotized copulatory ducts; embolus long with distal part resting on the subtegulum and the presence of dorsomedian process on the cymbium. | Zhu, Song & Zhang 2003, Caleb, Ghosh & Kumar 2018 |
| 73 | *Tetragnatha mandibulata* | Male chelicerae with a simple apophysis; without prominence and promarginal first largest tooth; promarginal guide tooth present; embolus and apical process of conductor blunt to rounded. Female fang with an outgrowth on the outer edge, near its base. | Okuma 1988; Barrion & Litsinger 1995; Zhu, Song & Zhang 2003 |
| 74 | *Tetragnatha hasselti* | Chelicerae long and slender, basal segment is shorter than carapace; cheliceral fang without cusps. The seminal vesicle is more or less kidney-shaped. | Okuma 1988 |
| 75 | *Tetragnatha vermiformis* | Abdomen yellowish-brown to silvery white; female cheliceral retromargin with seven teeth, apical tooth isolated; epigynal fold with a moderately long process | Okuma 1988; Barrion & Litsinger 1995 |
| 76 | *Leucauge celebesiana* | Cephalothorax brownish yellow; abdomen silvery white with a pair of longitudinal black stripes near the posterior end; epigyne with a transverse C-shaped orifice, posterior epigynal margin straight and anterior margin bulged. | Tikader 1982; Zhu, Song & Zhang 2003 |
| 77 | *Tylorida striata* | Female abdomen triangular with paired shoulder humps and without caudal tubercle; covered with silvery patches similar to broken mirror pieces; flat epigynal plate; copulatory ducts with sharp median twists and numerous wrinkles. Male cheliceral retromargin without tubercles | Sankaran *et al*. 2017 |
| 78 | *Lyrognathus crotalus* | Embolus long, lanceolate with a spatulate tip. | West & Nunn 2010 |
| 79 | *Argyrodes flavescens* | Carapace reddish-brown; abdomen is pale yellow with several pairs of silver spots on the dorsal and lateral surface. | Tanikawa, Chida & Kumada 1996; Javed, Srinivasulu & Tampal 2010 |
| 80 | *Argyrodes projeles* | Cephalothorax dark brown; abdomen silver white, protrudes posteriorly; lateral side of abdomen covered with black patches; epigyne oval. | Tikader 1970 |
| 81 | *Ariamnes simulans* | Cephalothorax yellowish with lateral yellow-brown markings; abdomen cylindrical, posterior extremity tapers into a long caudal appendage, extremity of tail pointed and covered with dark hairs; epigyne oval and divided by a longitudinal septum. | Pickard-Cambridge 1892 |
| 82 | *Thwaitesia* sp. | Posterior median eyes less than their diameter apart; abdomen covered by silver plates, similar to broken mirror pieces | Levi & Levi 1962 |
| 83 | *Pardosa procurva* | Carapace and abdomen dark brown; epigyne with a pair of anterior conical pockets placed side by side making an M-shaped outline, median septum narrow, spermathecae long with bulbous head | Yu & Song 1988; Yin *et al*. 2012 |
| 84 | *Nesticodes rufipes* | Abdomen oval and without any distinct cardiac pattern; epigyne with a spherical sclerotized plate, large spermathecae; the conductor of male palpus large, and embolus forming a stout tube. | Levy 1998; Yoshida 2001 |
| 85 | *Nihonhimea mundula* | Abdomen with leaf shaped pattern dorsally; epigyne with simple openings in the posterio medial portion, copulatory ducts short and not twisted. | Chrysanthus 1963 |
| 86 | *Thomisus unidentatus* | Female epigyne with two sclerotized semicircular orifices, spermathecae with large curved bursa. Male palps with single basal tibial apophysis. | Kiany *et al.* 2017 |
| 87 | *Indoxysticus* sp. | Carapace with numerous long setae, dark red patches laterally, abdomen with black and white markings. | Benjamin & Jaleel 2010 |
| 88 | *Synema* sp. | Carapace with long setae, lateral eyes on tubercles; abdomen globular with brown pataches | Barrion & Litsinger 1995 |
| 89 | *Camaricus formosus* | Abdomen subglobular, brown, longitudinally and laterally by white bands. Epigyne simple with a transverse W-shaped band and a semitriangular sclerotized plate. | Tikader 1980  Barrion & Litsinger 1995 |
| 90 | *Uloborus* sp. | Posterior eye row recurved; cephalic region at anterior lateral eyes about half the carapace width; calamistrum half as long as metatarsus IV; abdomen elongate, wider anteriorly and narrowing posteriorly. | Opell 1979 |
| 91 | *Zosis geniculata* | Abdomen ovoid, laterally with an anterodorsal hump; epigyne basin-like with lateral flanges. | Opell 1979 |
| 92 | *Mallinella* sp. | Carapace pear-shaped, anterior eye row slightly procurved, posterior eye row strongly procurved in dorsal view; abdomen ovoid with numerous pale patches, ventral region with posterior spines in a single row on weakly sclerotized area in front of spinnerets. | Dankittipakul, Jocqué & Singtripop 2012 |
| 93 | *Cyclosa quinqueguttata* | Abdomen with posterior end bifurcated, presence of a conical protuberance posteriorly and a pair of small tubercles ventrally, between the epigyne and spinnerets. | Tikader 1982; Tanikawa 1992 |
| 94 | *Chalcotropis pennata* | Cephalothorax reddish-yellow, abdomen long, brown to bright yellow; epigyne with two oval windows, copulatory ducts short, spermatheca globular. | Prószyński 1984 |
| 95 | *Hamataliwa* sp. 2 | Carapace rectangular in lateral view; clypeus and femora devoid of any black stripes; abdomen without geometric pattern. | Deeleman-Reinhold 2009 |
| 96 | *Clubiona* sp. 2 | Yellow, brown species, thoracic groove present; posterior eye row wider than anterior row, posterior median eyes closer to posterior lateral eyes than to each other; leg IV longer than leg I. | Majumder & Tikader 1991; Barrion & Litsinger 1995; Deeleman-Reinhold 2001 |
| 97 | *Oxyopes sakuntalae* | Abdomen broad, ventral side of the abdomen pale with a conspicuous longitudinal broad black line at the middle, extending from epigastric fold to base of spinnerets. Epigyne identical to that illustrated in Tikader (1970) | Tikader 1970 |
| 98 | *Meotipa sahyadri* | Small epigyne with copulatory ducts longer than diameter of spermatheca; epigynal projection emerging out medially from atrium, head trifid. | Kulkarni *et al*. 2017 |
| 99 | *Scytodes thoracica* | Cephalothorax yellow with numerous black markings; abdomen short, ovoid and with similar markings as in cephalothorax; epigyne with only a pair of oblique sclerotized plates; copulatory ducts coiled. | Özkütük *et al.* 2013 |
| 100 | *Pardosa pseudoannulata* | Epigyne with a pair of anterior hood; median septum narrower anteriorly and wider posteriorly; copulatory ducts thick; spermathecae small and globular with tubular structures. | Tikader & Malhotra 1980 (sub. *Pardosa annandalei*) |
| 101 | *Epidius parvati* | Carapace and abdomen light yellow; abdomen with green yellow dorsal folium; embolus bifurcate, bulbus ovoid, conductor with sharp end pointing ectally. | Benjamin 2000 |

**Table S3: Results of the Automatic Barcode Gap Discovery (ABGD) analyses. X, relative gap width; Simple, p-distance; Jukes-Cantor substitution model (JC69); Kimura 2-parameter substitution model (K2P).**

| Prior intra-specific distance (P) | | | | | | | | | |
| --- | --- | --- | --- | --- | --- | --- | --- | --- | --- |
| Model | X | Partition | 0.021544 | 0.012915 | 0.007743 | 0.004642 | 0.00278 | 0.001668 | 0.001000 |
| Simple | 1.5 | Initial | 103 | 102 | 102 | 102 | 113 | 145 | 145 |
|  |  | Recursive | - | 103 | 106 | 108 | 117 | - | - |
| JC | 1.5 | Initial | 102 | 92 | 112 | 102 | 112 | 112 | 112 |
|  |  | Recursive | 103 | 102 | - | 119 | 132 | 205 | 205 |
| K2P | 1.5 | Initial | 102 | 102 | 102 | 102 | 102 | 242 | 242 |
|  |  | Recursive | 103 | 104 | 106 | 122 | 131 | 296 | 296 |

**Table S4: Results of the General Mixed Yule-coalescent (GMYC) analyses.** Clusters, MOTUs delineated by the GMYC model with multiple specimens; Entities, singleton MOTU delineated by GMYC; CI, confidence interval; Likelihood null, likelihood of the null model; Likelihood GMYC, likelihood of the GMYC model; Threshold, the threshold between the speciation and coalescence processes; Single, single-threshold model; Multiple, multiple-threshold model.

| **Analysis** | **Clusters**  **(CI)** | **Entities**  **(CI)** | **Likelihood**  **null** | **Likelihood**  **GMYC** | **Likelihood**  **ratio** | **Threshold** |
| --- | --- | --- | --- | --- | --- | --- |
| Single | 78(74-79) | 115(109-120) | 4233.655 | 4388.633 | 309.956 | -0.009813138 |
| Multiple | 74(73-79) | 153(152-162) | 4233.655 | 4391.515 | 315.7218 | -0.01097289  -0.006848784  -0.004902723  -0.002716638 |

**Table S5: Sequences acquired from GenBank for constructing NJ tree in Fig. 6 to depict the relation with *Linyphia*** ***sikkimensis* with other species.**

| **Sl. No.** | **Family** | **Species** | **Accession No.** |
| --- | --- | --- | --- |
| 1. | Theridiidae | *Chrysso octomaculata* | JN817099 |
| 2. | Theridiidae | *Theridion varians* | KY269874 |
| 3 | Theridiidae | *Chrysso nordica* | KP650244 |
| 4. | Theridiidae | *Chrysso nordica* | KP653328 |
| 5. | Linyphiidae | *Neriene montanav* | KY269836 |
| 6. | Linyphiidae | *Neriene clathrata* | KY269027 |
| 7. | Linyphiidae | *Neriene clathrata* | KY270375 |
| 8. | Linyphiidae | *Neriene emphana* | KY270192 |
| 9. | Linyphiidae | *Neriene peltata* | KY270380 |
| 10. | Linyphiidae | *Neriene peltata* | KY268862 |
| 11. | Linyphiidae | *Linyphia triangularis* | KY269839 |
| 12. | Linyphiidae | *Linyphia triangularis* | KY270305 |
| 13. | Linyphiidae | *Linyphia triangularis* | KY270457 |
| 14. | Linyphiidae | *Linyphia hortensis* | KY270405 |
| 15. | Linyphiidae | *Linyphia hortensis* | KY270434 |
| 16. | Linyphiidae | *Neriene radiata* | KY270121 |
| 17. | Linyphiidae | *Neriene radiata* | KY268685 |

**Cross references for species identification and morphological examination.**

Aharon, S., Huber, B. A. & Gavish-Regev, E. Daddy-long-leg giants: revision of the spider genus *Artema* Walckenaer, 1837 (Araneae, Pholcidae). *Eur. J. Taxon.* **376**, 1-57 (2017).

Baehr, M. & Baehr, B. The Hersiliidae of the Oriental Region including New Guinea. Taxonomy, phylogeny, zoogeography (Arachnida, Araneae). *Spixiana* **19**, 1-96 (1993).

Barrion, A. T. & Litsinger, J. A.  Riceland spiders of South and Southeast Asia. CAB International Wallingford, UK, xix, 700 pp. (1995).

Bayer, S. The lace-sheet-weavers - a long story (Araneae: Psechridae: *Psechrus*). *Zootaxa* **3379**, 1-170 (2012).

Benjamin, S. P. *Epidius parvati* sp. n., a new species of the genus *Epidius* from Sri Lanka (Araneae: Thomisidae). *Bull. Br. Arachnol. Soc.***11**, 284-288 (2000).

Berry, J. W., Beatty, J. A. & Prószyński, J. Salticidae of the Pacific Islands. II. Distribution of nine genera, with descriptions of eleven new species. *J. Arachnol.* **25**, 109-136 (1997).

Buchar, J. Lycosidae aus dem Nepal-Himalaya. II. Die *Pardosa nebulosa*- und *P. venatrix*-Gruppe (Araneae: Lycosidae: Pardosinae). *Senckenb. Biol.* **61**, 77-91 (1980).

Buchar, J. Über einige Lycosiden (Araneae) aus Nepal. *Ergebnisse des Forschungsunternehmens Nepal Himalaya* **5**, 201-227 (1976).

Buchar, J.  Lycosidae aus Bhutan 1. Venoniinae und Lycosinae (Arachnida: Araneae). *Entomol. Basil.* **20**, 5-32 (1997).

Caleb, J. T. D., Chatterjee, S., Tyagi, K., Kundu, S. & Kumar, V.  Two new jumping spiders of the genera *Epocilla* Thorell, 1887 and *Mogrus* Simon, 1882 from India (Araneae: Salticidae). *Arthropoda Sel.* **26**, 329-334 (2017a).

Caleb, J. T. D., Ghosh, D. & Kumar, V.  On two new synonyms of the orb-weaving spider *Orsinome vethi* (Hasselt, 1882) (Araneae, Tetragnathidae). *Zootaxa* **4444**, 342-346 (2018).

Caleb, J. T. D., Mondal, K. & Kumar, V.  A new species of the huntsman spider genus *Pseudopoda* Jäger (Araneae: Sparassidae) from the Eastern Himalayas, India. *Halteres* **9**, 170-175 (2018).

Caleb, J. T. D., Pravalikha, G. B., Johnson, B. E., Manyu, M., Mungkung, S. & Mathai, M. T. *Hersilia aadi* Pravalikha, Srinivasulu & Srinivasulu, 2014 a junior synonym of *Hersilia savignyi* Lucas, 1836 (Araneae: Hersiliidae). *Zootaxa* **4254**, 396-400 (2017b).

Cao, Q., Li, S. Q. & Żabka, M. The jumping spiders from Xishuangbanna, Yunnan, China (Araneae, Salticidae). *ZooKeys* **630**, 43-104 (2016).

Chrysanthus, P. Spiders from south New Guinea V. *Nova Guinea, Zool.* **24**, 727-750 (1963).

Coddington, J. A., Kuntner, M. & Opell, B. D.  Systematics of the spider family Deinopidae with a revision of the genus *Menneus*. *Smithson. contrib. Zool.* **636**, 1-61 (2012).

Dankittipakul, P. & Beccaloni, J. Validation and new synonymies proposed for *Cheiracanthium* species from South and Southeast Asia (Araneae, Clubionidae). *Zootaxa* **3510**, 77-86 (2012).

Dankittipakul, P. & Singtripop, T. (2010). The spitting spider family Scytodidae in Thailand, with descriptions of three new *Dictis* species (Araneae). *Revue Suisse de Zool.* **117**, 121-141.

Dankittipakul, P., Jocqué, R. & Singtripop, T.  Systematics and biogeography of the spider genus *Mallinella* Strand, 1906, with descriptions of new species and new genera from Southeast Asia (Araneae, Zodariidae). *Zootaxa* **3369**, 1-327 (2012).

Deeleman-Reinhold, C. L. & Prinsen, J. D. *Micropholcus fauroti* (Simon) n. comb., a pantropical, synanthropic spider (Araneae: Pholcidae). *Entomol. Ber. (Amst.)* **47**, 73-77 (1987).

Deeleman-Reinhold, C. L.  Forest spiders of South East Asia: with a revision of the sac and ground spiders (Araneae: Clubionidae, Corinnidae, Liocranidae, Gnaphosidae, Prodidomidae and Trochanterriidae [sic]). Brill Leiden, 591 pp (2001).

Deeleman-Reinhold, C.L.  Description of the lynx spiders of a canopy fogging project in northern Borneo (Araneae: Oxyopidae), with description of a new genus and six new species of *Hamataliwa*. *Zool. Meded.* **83**, 673-700 (2009).

Gravely, F. H. The spiders and scorpions of Barkuda Island. *Rec. Ind. Mus. Calc.* **22**, 399-421 (1921).

Harvey, M. S., Austin, A. D. & Adams, M. The systematics and biology of the spider genus *Nephila* (Araneae: Nephilidae) in the Australasian region. *Invertebr. Syst.* **21**, 407-451 (2007).

Huber, B. A., Deeleman-Reinhold, C. L. & Pérez, G. A. The spider genus *Crossopriza* (Araneae, Pholcidae) in the New World. *Am. Mus. Novit.* **3262**, 1-10 (1999).

Jäger, P. & Praxaysombath, B. Spiders from Laos: new species and new records (Arachnida: Araneae). *Acta Arachnol.* **58**, 27-51 (2009).

Jäger, P. *Sinopoda*, a new genus of Heteropodinae (Araneae, Sparassidae) from Asia. *J. Arachnol.* **27**, 19-24 (1999).

Jäger, P. *Heteropoda* Latreille, 1804: new species, synonymies, transfers and records (Araneae: Sparassidae: Heteropodinae). *Arthropoda Sel.* **23**, 145-188 (2014).

Jastrzębski, P. Salticidae from the Himalayas. Genus *Menemerus* Simon, 1868 (Araneae: Salticidae). *Entomol. Basil.* **20**, 33-44 (1997a).

Jastrzębski, P. Salticidae from the Himalayas. Genus *Rhene* Thorell, 1869 (Araneae: Salticidae). *Entomol. Basil.* **20**, 45-56 (1997b).

Javed, S. M. M., Srinivasulu, C. & Tampal, F. Addition to araneofauna of Andhra Pradesh, India: occurrence of three species of *Argyrodes* Simon, 1864 (Araneae: Theridiidae). *J. Threat. Taxa* **2**, 980-985 (2010).

Kiany, N., Sadeghi, S., Kiany, M., Zamani, A. & Ostovani, S. Additions to the crab spider fauna of Iran (Araneae: Thomisidae). *Arachnol. Mitt.* **53**, 1-8 (2017).

Kulkarni, S. & Joseph, S. First record of genus *Siler* Simon, 1889 (Araneae: Salticidae) from India. *J. Threat. Taxa* **7**, 7701-7703 (2015).

Kulkarni, S., Vartak, A., Deshpande, V. & Halali, D. The spiny theridiid genus *Meotipa* Simon, 1895 in India, with description of a strange new species with translucent abdomen and a phylogenetic analysis about the genus placement (Araneae, Theridiidae). *Zootaxa* **4291**, 504-520 (2017).

Kullmann, E. & Zimmermann, W. Beschreibung der neuen Spinnenart *Oecobius afghanicus* mit ergänzenden Angaben zu *Oecobius putus* und *Oecobius annulipes* (Arachnida: Araneae: Oecobiidae). *Entomol. Ger.* **3**, 41-50 (1976).

Levi, H. W. & Levi, L. R. The genera of the spider family Theridiidae. *Bull. Mus. Comp. Zool.* **127**, 1-71 (1962).

Levi, H. W. The orb-weaver genera *Argiope*, *Gea*, and *Neogea* from the western Pacific region (Araneae: Araneidae, Argiopinae). *Bull. Mus. Comp. Zool.* **150**, 247-338 (1983).

Levy, G. Araneae: Theridiidae. In: Fauna Palaestina, Arachnida III. Israel Academy of Sciences and Humanities, Jerusalem, 228 pp (1998).

Majumder, S. C. & Tikader, B. K.  Studies on some spiders of the family Clubionidae from India. *Rec. Zool. Surv. Ind.* **102**, 1-175 (1991).

Malamel, J. J., Pradeep, M. S. & Sebastian, P. A. *Fecenia travancoria* Pocock is recognised as a junior synonym of *Fecenia protensa* Thorell (Araneae: Psechridae): a case of intraspecific variation. *Zootaxa* **3741**, 359-368 (2013).

Mi, X. Q. & Wang, C.  First description on the female of *Eriovixia huwena* and the male of *E. poonaensis* (Araneae, Araneidae). *Sichuan J. Zool.* **35**, 728-733 (2016).

Mi, X. Q., Peng, X. J. & Yin, C. M. The orb-weaving spider genus *Eriovixia* (Araneae: Araneidae) in the Gaoligong mountains, China. *Zootaxa* **2488**, 39-51 (2010).

Okuma, C. A revision of the genus *Tetragnatha* Latreille (Araneae, Tetragnathidae) of Asia, Part II. *J. Fac Agr. Kyushu U.* **32**, 183-213 (1988).

Opell, B. D. Revision of the genera and tropical American species of the spider family Uloboridae. *Bull. Mus. Comp. Zool.* **148**, 443-549 (1979).

Özkütük, R. S., Marusik, Y. M., Danişman, T., Kunt, K. B., Yağmur, E. A. & Elverici, M. Genus *Scytodes* Latreille, 1804 in Turkey (Araneae, Scytodidae). *Hacettepe J. Biol. Chem.* **41**, 9-20 (2013).

Pickard-Cambridge, O. On a new spider from Calcutta. *Ann. Mag. Nat. Hist.* **10**, 417-419 (1892).

Pocock, R.I. Descriptions of some new species of spiders from British India. *J. Bombay Nat. Hist. Soc.* **13**, 478-498 (1901).

Próchniewicz, M. Salticidae aus Nepal und Bhutan. Genera *Telamonia* Thorell 1887 und *Plexippoides* Prószyński 1976 (Arachnida: Araneae). *Senckenb. Biol.* **70**, 151-160 (1990).

Prószyński, J. & Deeleman-Reinhold, C. L. Description of some Salticidae (Araneae) from the Malay Archipelago. III. Salticidae of Borneo, with comments on adjacent territories. *Arthropoda Sel.* **22**, 113-144 (2013).

Prószyński, J. Atlas rysunków diagnostycznych mniej znanych Salticidae (Araneae). *Wyższa Szkola Rolniczo-Pedagogiczna, Siedlcach* **2**, 1-177 (1984).

Prószyński, J. Salticidae (Araneae) of India in the collection of the Hungarian National Natural History Museum in Budapest. *Ann. Zool.* **44**, 165-277 (1992).

Roy, T. K., Sen, S., Saha, S. & Raychaudhuri, D. A new *Chorizopes* O.P.-Cambridge, 1870 (Araneae: Araneidae) from West Bengal, India. *Rom. J. Biol. -Zool.* **59**, 3-9 (2014).

Sankaran, P. M., Malamel, J. J., Joseph, M. M. & Sebastian, P. A. On the genus *Tylorida* Simon, 1894 with the first record of the genus *Atelidea* Simon, 1895 from India (Araneae: Tetragnathidae, Leucauginae). *Zootaxa* **4353**, 294-326 (2017).

Sherriffs, W. R. Some oriental spiders of the genus *Oxyopes*. *Proc. Zool. Soc. London.* **120**, 651-677 (1951).

Tan, J., Chan, Z.J., Ong, C.A. & Yong, H.S. Phylogenetic relationships of *Actinacantha* Simon, *Gasteracantha* Sundevall, *Macracantha* Hasselt and *Thelacantha* Simon spiny orbweavers (Araneae: Araneidae) in Peninsular Malaysia. *Raf. Bull. Zool.* **67**, 32-55 (2019).

Tanikawa, A. A revisional study of the Japanese spiders of the genus *Cyclosa* (Araneae: Araneidae). *Acta Arachnol.* **41**, 11-85 (1992).

Tanikawa, A., Chida, T. & Kumada, K. I. New records of *Argyrodes flavescens* (Araneae: Theridiidae) from Japan. *Acta Arachnol.* **45**, 47-52 (1996).

Tikader, B. K. & Biswas, B. Spider fauna of Calcutta and vicinity: Part-I. *Rec. Zool. Surv. Ind.* **30**, 1-149 (1981).

Tikader, B. K. & Malhotra, M. S. Lycosidae (Wolf-spiders). Fauna India (Araneae) **1**, 248-447 (1980).

Tikader, B. K. Family Araneidae (=Argiopidae), typical orbweavers. Fauna India (Araneae) **2**, 1-293 (1982).

Tikader, B. K. Spider fauna of Sikkim. *Rec. Zool. Surv. Ind.* **64**, 1-83 (1970).

Tikader, B. K. Thomisidae (Crab-spiders). Fauna India (Araneae) **1**, 1-247 (1980).

Tikader, B. K.  Studies on some spiders of the genus *Oecobius* (family Oecobiidae) from India. *J. Bombay Nat. Hist. Soc.* **59**, 682-685 (1962).

Wang, D. & Zhang, Z. S. Two new species and a new synonym in the *Pardosa nebulosa*-group (Lycosidae: *Pardosa*) from China. *Zootaxa* **3856**, 227-240 (2014).

Wesolowska, W & Freudenschuss, M. A new species of *Menemerus* from Pakistan (Araneae: Salticidae). *Genus* **23**, 449-453 (2010).

Wesolowska, W. A revision of the spider genus *Menemerus* in Africa (Araneae: Salticidae). *Genus* **10**, 251-353 (1999).

West, R. C. & Nunn, S. C. A taxonomic revision of the tarantula spider genus *Lyrognathus* Pocock 1895 (Araneae, Theraphosidae), with notes on the Selenocosmiinae. *Zootaxa* **2362**, 1-43 (2010).

Yin, C. M., Wang, J. F., Xie, L. P. & Peng, X. J. New and newly recorded species of the spiders of family Araneidae from China (Arachnida, Araneae). In: Spiders in China: One Hundred New and Newly Recorded Species of the Families Araneidae and Agelenidae. Hunan Normal University Press, 1-171 pp. (1990).

Yoshida, H. A revision of the Japanese genera and species of the subfamily Theridiinae (Araneae: Theridiidae). *Acta Arachnol.* **50**, 157-181 (2001).

Yu, L. M. & Song, D. X. On new species of the genus *Pardosa* from China (Araneae: Lycosidae). *Acta Zootaxon. Sin.* **13**, 27-41 (1988).

Żabka, M. & Gardzińska, J. Salticidae of Thailand. Part 1, genera *Plexippus* C. L. Koch, 1846 and *Burmattus* Prószyński, 1992. *Ann. Zool.* **67**, 229-242 (2017).

Żabka, M. New species of *Yaginumaella* Prószyński 1976 and *Helicius* Prószyński 1976 (Araneae, Salticidae) from Bhutan and Burma. *Entomol. Basil.* **6**, 5-41 (1981).

Żabka, M. Systematic and zoogeographic study on the family Salticidae (Araneae) from Viet-Nam. *Ann. Zool.* **39**, 197-485 (1985).

Zhu, M. S., Song, D. X. & Zhang, J. X. Fauna Sinica: Invertebrata Vol. 35: Arachnida: Araneae: Tetragnathidae. Science Press, Beijing, vii, 418 pp (2003).

Zonstein, S. L. & Marusik, Y. M. A redescription of *Damarchus cavernicola* Abraham, 1924, with notes on *Damarchus* Thorell, 1891 and *Atmetochilus* Simon, 1887 (Aranei: Nemesiidae). *Arthropoda Sel.* **23**, 273-278 (2014).
